# Supplementary material for: 1,2σ3λ3-Oxaphosphetanes and Their P-Chalcogenides—A Combined Experimental and Theoretical Study
Source: Molecules. 2022 May 23;27(10):3345. doi: 10.3390/molecules27103345 (PMC9147792; doi:10.3390/molecules27103345)
Supplement: Supplementary file 1 [file molecules-27-03345-s001.zip › molecules-1729439-supplementary.pdf]

Supporting Information

1,2σ³λ³-Oxaphosphetanes and their *P*-chalcogenides – a combined experimental and theoretical study

Florian Gleim, Antonio García Alcaraz, Gregor Schnakenburg, Arturo Espinosa Ferao * and Rainer Streubel *

Content

EXPERIMENTAL PART **S3**

General experimental details **S3**

Synthetic procedures, analytical data, and NMR spectra **S3**

Figure S1: ^{1}H-NMR spectrum of **3b,b′** S4

Figure S2: ^{31}P{^{1}H}-NMR spectrum of **3b,b′** S5

Figure S3: ^{13}C{^{1}H}-NMR spectrum of **3b,b′** S5

Figure S4: ^{1}H-NMR spectrum of **3c,c′** S6

Figure S5: ^{31}P{^{1}H}-NMR spectrum of **3c,c′** S7

Figure S6: ^{13}C{^{1}H}-NMR spectrum of **3c,c′** S7

Figure S7: ^{1}H-NMR spectrum of **3d,d′** S8

Figure S8: ^{1}H-NMR spectrum of **3d,d′** S9

Figure S9: ^{13}C{^{1}H}-NMR spectrum of **3d,d′** S9

Figure S10: ^{31}P{^{1}H}-NMR spectrum of **3e,e′** and **3e*,e*′** S10

Figure S11: ^{1}H-NMR spectrum of **4b,b′** S11

Figure S12: ^{31}P{^{1}H}-NMR spectrum of **4b,b′** S12

Figure S13: ^{13}C{^{1}H}-NMR spectrum of **4b,b′** S12

Figure S14: ^{1}H-NMR spectrum of **4c,c′** S13

Figure S15: ^{31}P{^{1}H}-NMR spectrum of **4c,c′** S14

Figure S16: ^{13}C{^{1}H}-NMR spectrum of **4c,c′** S14

Figure S17: ^{1}H-NMR spectrum of **4d,d′** S15

Figure S18: ^{31}P{^{1}H}-NMR spectrum of **4d,d′** S16

Figure S19: ^{13}C{^{1}H}-NMR spectrum of **4d,d′** S16

Figure S20: ^{1}H-NMR spectrum of **14a,a′** S17

Figure S21: ^{31}P{^{1}H}-NMR spectrum of **14a,a′** S18

Figure S22: ^{13}C{^{1}H}-NMR spectrum of **14a,a′** S18

Figure S23: ^{1}H-NMR spectrum of **15a,a′** S19

Figure S24: ^{31}P{^{1}H}-NMR spectrum of **15a,a′** S20

Figure S25: ^{13}C{^{1}H}-NMR spectrum of **15a,a′** S20

Figure S26: ^{1}H-NMR spectrum of **16a,a′** S21

Figure S27: ^{31}P{^{1}H}-NMR spectrum of **16a,a′** S22

Figure S28: ^{13}C{^{1}H}-NMR spectrum of **16a,a′** S22

Figure S29: ^{77}Se{^{1}H}-NMR spectrum of **16a,a′** S23

Crystal structure data of **3b,b′**, **3c,c′**, **3d,d′**, **4b,b′**, **and 4c,c′** **S24**

COMPUTATIONAL PART **S26**

Table S1: P-O bond properties of complexes **9** and **9^{OMe2}** **S26**

Table S2: C-O bond properties of **2e**, **2e-Li(DEGDME)** and **9** **S26**

Scheme S1: Mechanistic proposal for the formation of 1,2-oxaphosphetanes **12′** and **12b*′** from **7** **S26**

Figure S30: Calculated ($CPCM_{THF}$/CCSD(T)/def2-TZVPP(ecp)) minimum energy profile for the conversion of **7** into **12′** and **12*′** **S27**

Scheme S2: Nucleophilic attack of the P atom of **5^{tBu}** to C atoms of styrene oxide **2e** giving rise to **13^{tBu}** and **13^{tBu*}** **S28**

Figure S31: Calculated ($CPCM_{THF}$/CCSD(T)/def2-TZVPP(ecp)) minimum energy profile for the conversion of **5^{tBu}+2e** into **13^{tBu}** and **13^{tBu*}** **S28**

Scheme S3: Homodesmotic reactions for RSE evaluation on derivatives **VIa-e** **S29**

Calculated structures..**S30**

Refer-
ences……...………
…………………………………………………….……………………………..**S45**

EXPERIMENTAL PART

GENERAL EXPERIMENTAL DETAILS

The syntheses of all compounds were performed under an argon atmosphere, using common Schlenk techniques and dry solvents. Tetrahydrofuran, diethyl ether, and *n*-pentane were dried over sodium wire/benzophenone, CH_2Cl_2 over CaH_2, and further purified by subsequent distillation. All NMR spectra were recorded on a Bruker AVI-300 or a Bruker AV III HD Prodigy 500 spectrometer at 25°C. The ^{1}H and ^{13}C NMR spectra were referenced to the residual proton resonances and the ^{13}C NMR signals of the deuterated solvents and ^{31}P to 85% H_3PO_4 as external standards, respectively. Melting points were determined in one-side melted off capillaries using a Büchi Type S apparatus; the values are uncorrected. Mass spectrometric data were collected on a MAT 90 Finnigan sector mass spectrometer using LIFDI or on a Bruker Dalton micrOTOF-Q using ESI(+/-) or APCI. IR spectra of all compounds were recorded on a Thermo Nicolet 380 FT-IR spectrometer with an attenuated total reflection (ATR) attachment or a Bruker Alpha Diamond ATR FTIR spectrometer.

SYNTHETIC PROCEDURES, ANALYTICAL DATA, AND NMR SPECTRA

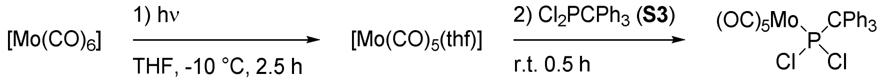

S1 **S2** **S4**

<u>Synthesis of complex **S4**</u>: 5.27 g (20.00 mmol, 1.33 eq) of $Mo(CO)_6$ (**S1**) was dissolved in 420 ml of THF in a 500 mL photolysis reactor. The solution was flushed with argon and cooled to -10°C. The solution was then irradiated using a Hg-lamp (200-280 nm, 150 W) for a period of 2.5 h and, after removal of the cooling, the solution was stirred for 15 min. The solution was then transferred into a flask containing 5.18 g (15 mmol, 01 eq) of Ph_3CPCl_2 (**S3**). After stirring for 30 min, the solvent was removed in vacuo (0.02 mbar). The remaining crude product was purified via column chromatography (h = 10 cm, d = 5 cm, r.t., Et_2O, Al_2O_3). After this, to regain unreacted **S1**, the crude product was sublimed (50°C, 0.05 mbar, 3.5 h). A second sublimation was carried out to remove $HCPh_3$ (100°C, 0.05 mbar, 2.0 h). The product was obtained as a beige to gray powder.

Yield: 7.0552 g, 12.12 mmol, 81% (25%). ^{1}H NMR (300.1 MHz, $CDCl_3$) δ (ppm) = 7.36 (m, 9H, *meta/para*-C*H*), 7.42 (m, 6H, *ortho*-C*H*). $^{31}P\{^{1}H\}$ NMR (121.5 MHz, $CDCl_3$) δ (ppm) = 201.5 (s).

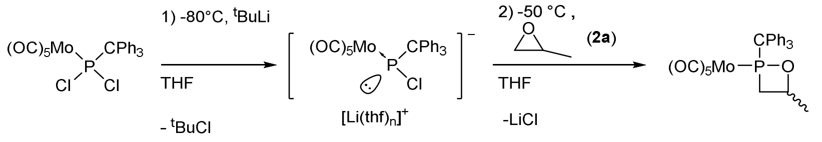

S4 **1** **3a,a'**

<u>Synthesis of complex **3a,a'**:</u> 1.944 g (3.42 mmol, 1 eq) of the Mo complex was dissolved in 40 ml of dried THF and cooled to -80°C. 2.39 mL (4.10 mmol, 1.2 eq) of a *tert*-butyllithium solution (1.7 M in *n*-pentane) was slowly added. The solution was kept stirring while slowly warming up. Upon reaching -50°C, 0.5 mL (10.26 mmol, 3 eq) 1,2-epoxypropane (**2a**) was added. The solution was further kept stirring while slowly warming up to ambient temperature. After reaching room temperature, all volatiles were removed in vacuo (0.02 mbar). The crude product was purified by filtration with 250 mL of Et_2O over Al_2O_3 (h = 10 cm, d = 3 cm, r.t.) and removal of the solvent in vacuo (0.02 mbar). The product was obtained as a white solid. The obtained analytical data are in accordance with the known literature [1].

Yield: 1.4492 g, 2.55 mmol, 75%. Ratio (Isomer 1: Isomer 2) = 51:49. **^{1}H NMR** (300.1 MHz, $CDCl_3$) δ (ppm) = **(Isomer 1)** 1.14 (d, 3H, $^{3}J_{H\text{-}H}$ = 6.2 Hz, -C*H*3), 2.95 (m, 2H, -C*H*2), 5.34 (m, 1H, -C*H*), 7.37 (m, 15H, -C*H*)1.60 (d, 3H, $^{3}J_{H\text{-}H}$ = 6.3 Hz, -C*H*3), 2.95 (m, 1H, -C*H*2), 3.17 (m, 1H, -C*H*2), 5.34 (m, 1H, -C*H*), 7.37 (m, 15H, -C*H*); **(Isomer 2)** 1.14 (d, 3H, $^{3}J_{H\text{-}H}$ = 6.2 Hz, -C*H*3), 2.95 (m, 2H, -C*H*2), 4.68 (m, 1H, -C*H*), 7.37 (m, 15H, -C*H*). **)** 1.60 (d, 3H, $^{3}J_{H\text{-}H}$ = 6.3 Hz, -C*H*3), 2.95 (m, 1H, -C*H*2), 3.17 (m, 1H, -C*H*2), 4.68 (m, 1H, -C*H*), 7.37 (m, 15H, -C*H*).**^{31}P{^{1}H} NMR** (121.5 MHz, $CDCl_3$) δ (ppm)= **(Isomer 1)** 185.6 (s); **(Isomer 2)** 207.2 (s).

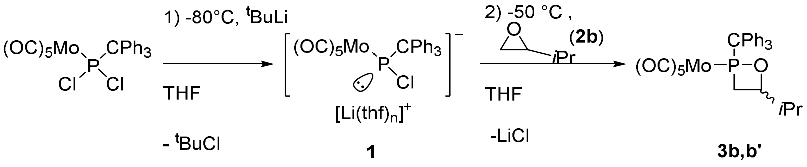

<u>Synthesis of complex **3b,b'**:</u> 0.047 g (0.80 mmol, 1 eq) of the Mo complex was dissolved in 20 ml of dried THF and cooled to -80°C. 0.56 mL (0.96 mmol, 1.2 eq) of a *tert*-butyllithium solution (1.7 M in *n*-pentane) was slowly added. The solution was kept stirring while slowly warming up. Upon reaching -50°C, 0.13 mL (1.2 mmol, 1.5 eq) 1,2-epoxy-3-methylbutane (**2b**) were added. The solution was further kept stirring while slowly warming up to ambient temperature. After reaching room temperature, all volatiles were removed in vacuo (0.02 mbar). The crude product was purified by filtration with 250 mL of Et_2O over Al_2O_3 (h = 10 cm, d = 3 cm, r.t.) and removal of the solvent in vacuo (0.02 mbar). To remove impurities of diethyl ether, the product was dissolved in *n*-pentane and dried in vacuo (0.02 mbar). The product was obtained as a white solid.

Yield: 0.41 g, 0.67 mmol, 86%. Ratio (Isomer 1: Isomer 2) = 50:50. Melting point: decomposition upon reaching 140°C. **^{1}H NMR** (300.1 MHz, $CDCl_3$) δ (ppm) = **(Isomer 1)** 0.54 (d, 3H, $^3J_{H-H}$ = 6.7 Hz, -C*H*$_3$), 0.93 (d, 3H, $^3J_{H-H}$ = 6.5 Hz, -C*H*$_3$), 1.33 (m, 1H, -C*H*), 2.90 (m, 2H, -C*H*$_2$), 4.54 (dddd, 1H, $^3J_{H-H}$ = 10.3 Hz, $^3J_{H-H}$ = 8.3 Hz, $^3J_{H-H}$ = 6.5 Hz, $^3J_{P-H}$ = 1.7 Hz, -C*H*), 7.26-7.55 (m, 15H, -C*H*); **(Isomer 2)** 0.68 (d, 3H, $^3J_{H-H}$ = 6.7 Hz, -C*H*$_3$), 0.98 (d, 3H, $^3J_{H-H}$ = 6.5 Hz, -C*H*$_3$), 2.35 (m, 1H, -C*H*), 2.99 (m, 2H, -C*H*$_2$), 4.97 (dddd, 1H, $^3J_{H-H}$ = 10.5 Hz, $^3J_{H-H}$ = 8.5 Hz, $^3J_{H-H}$ = 6.3 Hz, $^3J_{P-H}$ = 1.8 Hz, -C*H*), 7.26-7.55 (m, 15H, -C*H*). **^{13}C NMR** (75.5 MHz, $CDCl_3$) δ (ppm) = **(Isomer 1)** 15.9 (s, 1C, -CH_3), 18.3 (s, 1C, -CH_3), 35.0 (d, 1C, $^3J_{P-C}$ = 2.7 Hz, -CH), 37.5 (d, 1C, $^1J_{P-C}$ = 21.5 Hz, -CH_2), 67.1 (d, 1C, $^1J_{P-C}$ = 9.3 Hz, -CPh_3), 85.9 (d, 1C, $^2J_{P-C}$ = 11.3 Hz, -CH), 127.6 (s, 1C, *para*-CH), 128.4 (s, 2C, *meta*-CH), 130.9 (d, 2C, $^3J_{P-C}$ = 6.9 Hz, *ortho*-CH), 140.8 (s_{br}, 1C, *ipso*-C), 204.7 (d, 4C, $^2J_{P-C}$ = 5.4 Hz, *cis*-CO), 210.2 (d, 1C, $^2J_{P-C}$ = 15.1 Hz, *trans*-CO); **(Isomer 2)** 16.3 (s, 1C, -CH_3), 17.7 (s, 1C, -CH_2), 36.2 (d, 1C, $^3J_{P-C}$ = 2.8 Hz, -CH), 39.9 (d, 1C, $^1J_{P-C}$ = 18.5 Hz, -CH_2), 67.8 (d, 1C, $^1J_{P-C}$ = 10.7 Hz, -CPh_3), 90.1 (d, 1C, $^2J_{P-C}$ = 11.2 Hz, -CH), 127.7 (s, 1C, *para*-CH), 128.5 (s, 2C, *meta*-CH), 131.3 (d, 2C, $^3J_{P-C}$ = 7.1 Hz, *ortho*-CH), 141.9 (s_{br}, 1C, *ipso*-C), 204.8 (d, 4C, $^2J_{P-C}$ = 5.4 Hz, *cis*-CO), 210.6 (d, 1C, $^2J_{P-C}$ = 15.4 Hz, *trans*-CO). **^{31}P{^{1}H} NMR** (121.5 MHz, $CDCl_3$) δ (ppm)= **(Isomer 1)** 183.5 (s); **(Isomer 2)** 206.1 (s). **MS** (LIFDI) m/z (%) = 598.0 (100) $[M]^{•+}$, 243.1 (10) $[CPh_3]^+$. **IR ATR**: ṽ (cm^{-1}) = 2070 (s, ν(CO)), 1993 (m, ν(CO)), 1957 (s, ν(CO)), 1913 (s, ν(CO)).

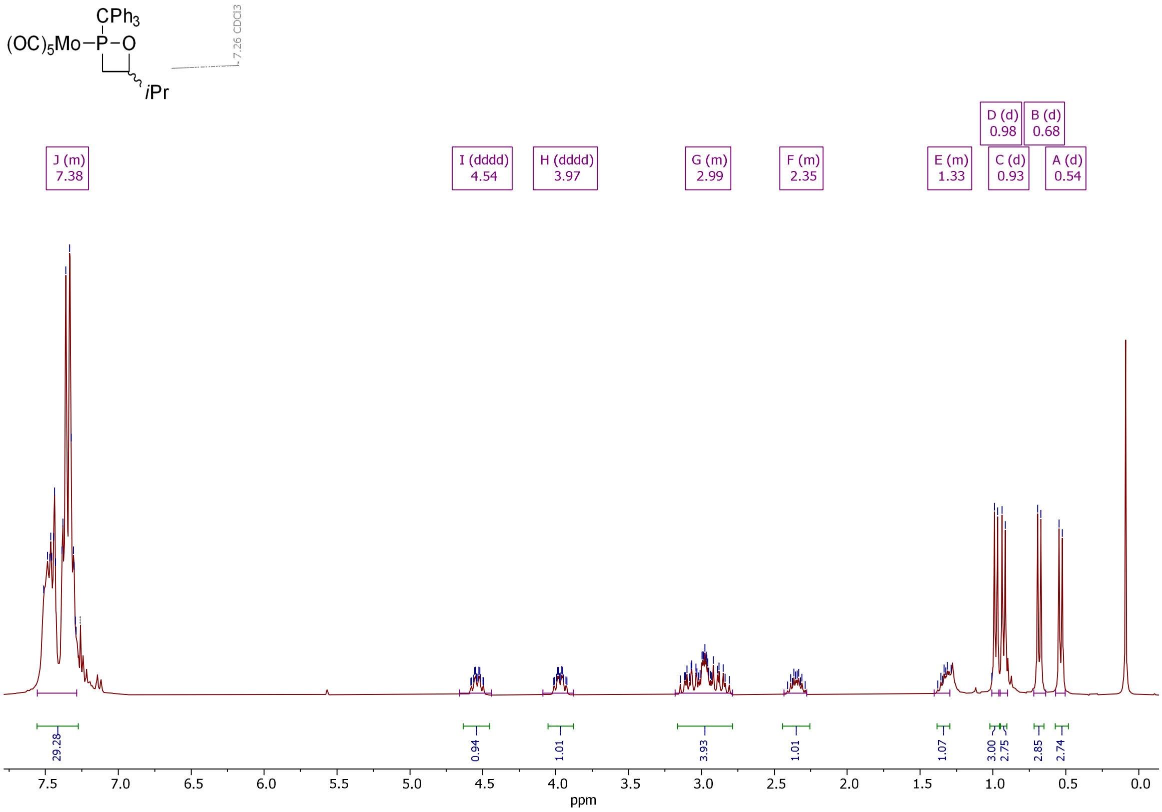

Figure S1: ^{1}H-NMR spectrum of **3b,b'** in $CDCl_3$.

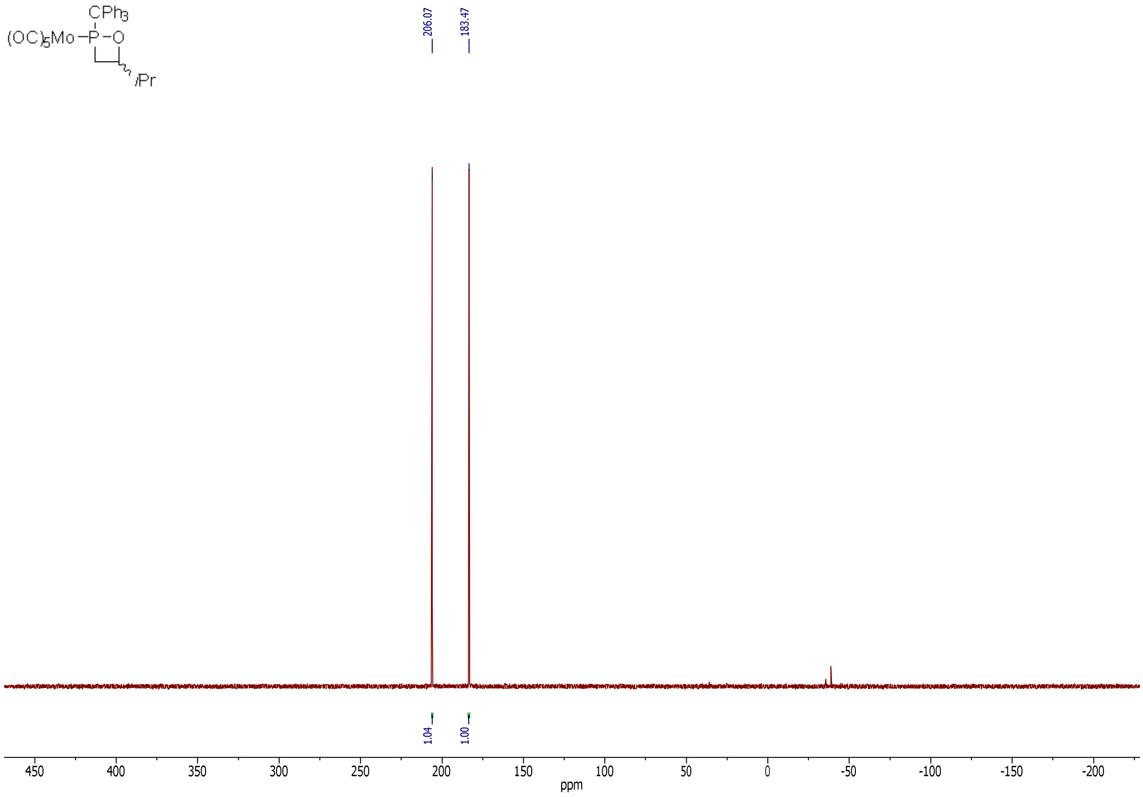

Figure S2: $^{31}P\{^{1}H\}$-NMR spectrum of **3b,b'** in $CDCl_3$.

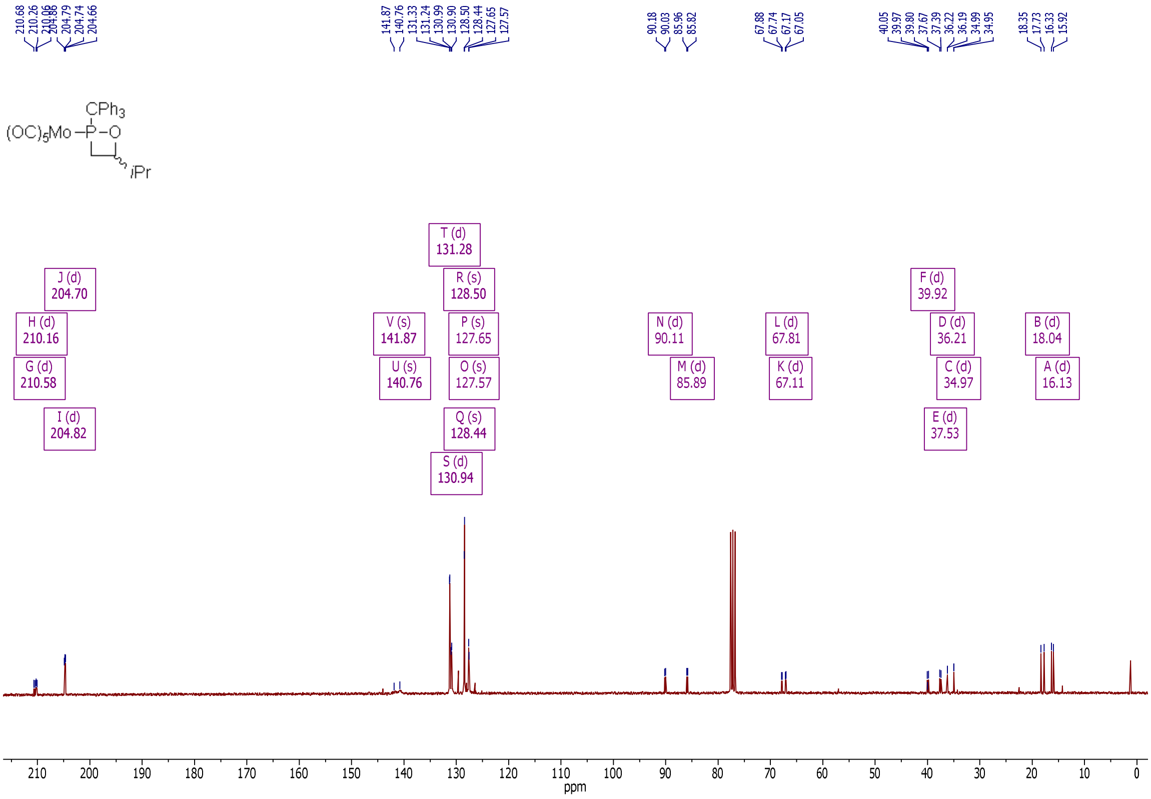

Figure S3: $^{13}C\{^{1}H\}$-NMR spectrum of **3b,b'** in $CDCl_3$.

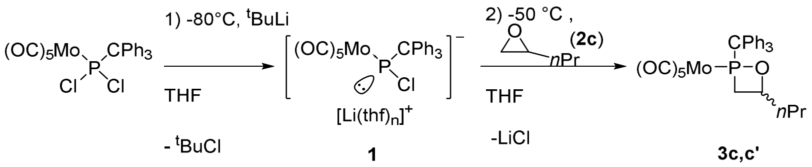

Synthesis of complex **3c,c'**: 1.16 g (2.00 mmol, 1 eq.) of the Mo complex was dissolved in 30 ml of dried THF and cooled to -80°C (ethanol cooling bath). 1.4 mL (2.4 mmol, 1.2 eq) of a *tert*-butyllithium solution (1.7 M in *n*-pentane) was slowly added. The solution was kept stirring while slowly warming up. Upon reaching -50°C, 0.3 mL (3 mmol, 1.5 eq) 1,2-epoxypentane (**2c**) were added. The solution was further kept stirring while slowly warming up to ambient temperature. After reaching room temperature, all volatiles were removed in vacuo (0.02 mbar). The crude product was purified by filtration with 150 mL of Et_2O over Al_2O_3 (h = 5 cm, d = 2 cm, r.t.) and removal of the solvent in vacuo (0.02 mbar). To remove traces of diethyl ether, the product was dissolved in *n*-pentane and dried in vacuo (0.02 mbar). The product was obtained as a light orange solid.

Yield: 1.17 g, 1.97 mmol, 98%. Ratio (Isomer 1: Isomer 2): 50:50. Melting point: decomposition above 100°C. **^{1}H NMR** (500.1 MHz, $CDCl_3$) δ (ppm) = **(Isomer 1)** 0.84 (t, 3H, $^3J_{H\text{-}H}$ = 7.3 Hz, -C*H*3), 1.17 (m, 2H, -C*H*2), 1.28 (m, H, -C*H*2), 1.45 (m, 1H, -C*H*2), 2.91 (m, 1H, -C*H*2), 3.00 (m, 1H, -C*H*2), 4.48 (m, 1H, -C*H*), 7.29-7.59 (m, 15H, -C*H*); **(Isomer 2)** 0.89 (t, 3H, $^3J_{P\text{-}H}$ = 7.4 Hz, -C*H*3), 1.28 (m, 2H, -C*H*2), 1.81 (m, H, -C*H*2), 2.10 (m, 1H, -C*H*2), 2.95 (m, 1H, -C*H*2), 3.12 (m, 1H, -C*H*2), 5.13 (m, 1H, -C*H*), 7.29-7.59 (m, 15H, -C*H*). **^{13}C NMR** (125.8 MHz, $CDCl_3$) δ (ppm) = **(Isomer 1)** 13.7 (s, 1C, $-CH_3$), 17.0 (s, 1C, $-CH_2$), 39.2 (d, 1C, $^3J_{P\text{-}C}$ = 2.6 Hz, $-CH_2$), 40.6 (d, 1C, $^1J_{P\text{-}C}$ = 18.3 Hz, $-CH_2$), 67.2 (d, 1C, $^1J_{P\text{-}C}$ = 9.3 Hz, $-CPh_3$), 80.9 (d, 1C, $^3J_{P\text{-}C}$ = 11.6 Hz, -CH), 127.6 (s, 1C, *para*-CH), 128.5 (s, 2C, *meta*-CH), 130.9 (s, 2C, *ortho*-CH), 140.8 (s, 1C, *ipso*-C), 204.7 (d, 4C, $^2J_{P\text{-}C}$ = 4.6 Hz, *cis*-CO), 210.2 (d, 1C, $^2J_{P\text{-}C}$ = 25.9 Hz, *trans*-CO); **(Isomer 2)** 13.7 (s, 1C, $-CH_3$), 17.9 (s, 1C, $-CH_2$), 38.5 (d, 1C, $^1J_{P\text{-}C}$ = 21.4 Hz, $-CH_2$), 40.6 (d, 1C, $^3J_{P\text{-}C}$ = 3.0 Hz, $-CH_2$), 67.9 (d, 1C, $^1J_{P\text{-}C}$ = 10.7 Hz, $-CPh_3$), 85.0 (d, 1C, $^3J_{P\text{-}C}$ = 11.4 Hz, -CH), 127.7 (s, 1C, *para*-CH), 128.5 (s, 2C, *meta*-CH), 131.3 (d, 2C, $^2J_{P\text{-}C}$ = 7.1 Hz, *ortho*-CH), 140.8 (s, 1C, *ipso*-C), 204.8 (d, 4C, $^2J_{P\text{-}C}$ = 4.5 Hz, *cis*-CO), 210.5 (d, 1C, $^2J_{P\text{-}C}$ = 26.1 Hz, *trans*-CO). **^{31}P NMR** (202.4 MHz, $CDCl_3$) δ (ppm) = **(Isomer 1)** 187.6 (d, $^2J_{P\text{-}H}$ = 11.6 Hz); **(Isomer 2)** 208.8 (d, $^2J_{P\text{-}H}$ = 10.4 Hz). **MS** (LIFDI) m/z (%) = 598.2 (100) $[M]^{\bullet+}$, 243.1 (10) $[CPh_3]^+$. **HRMS** (EI): theor./exp. 598.04428/598.04573 $[M]^+$. **IR ATR**: $\tilde{\nu}$ (cm^{-1}) = 2071 (s, ν(CO)), 1995 (m, ν(CO)), 1918 (s, ν(CO)).

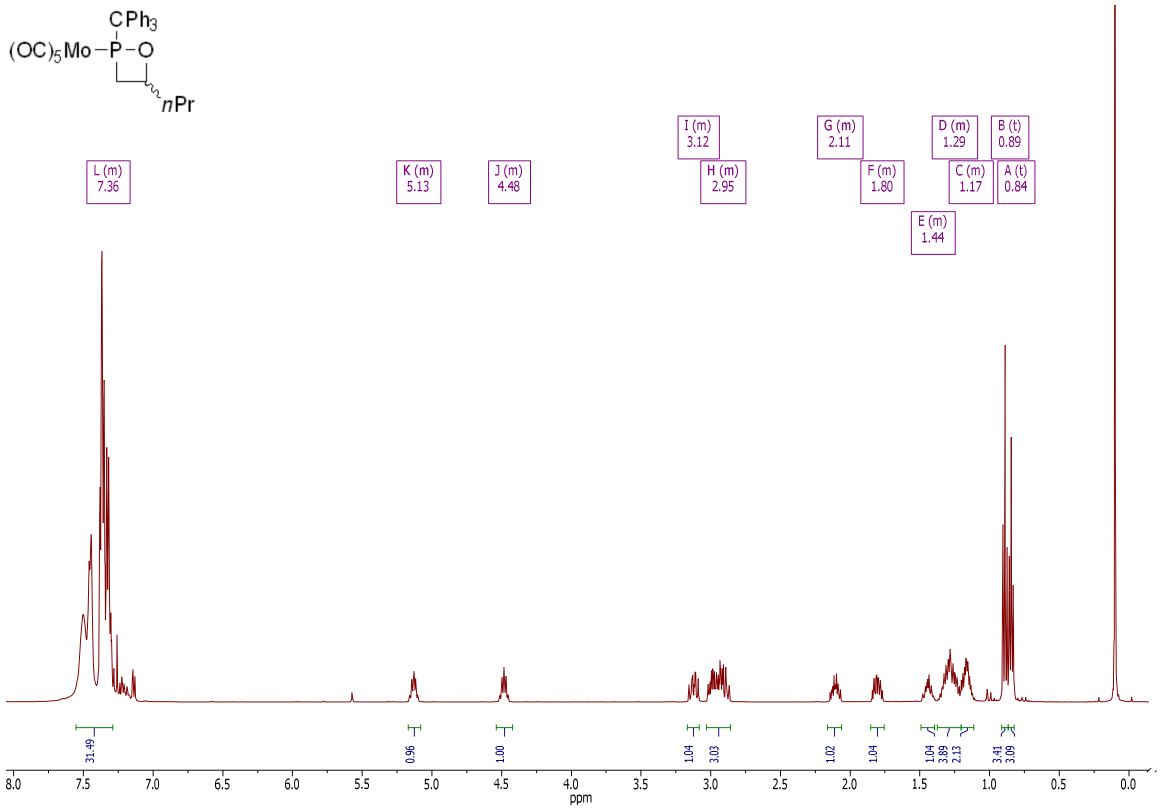

Figure S4: ^{1}H-NMR spectrum of **3c,c'** in $CDCl_3$.

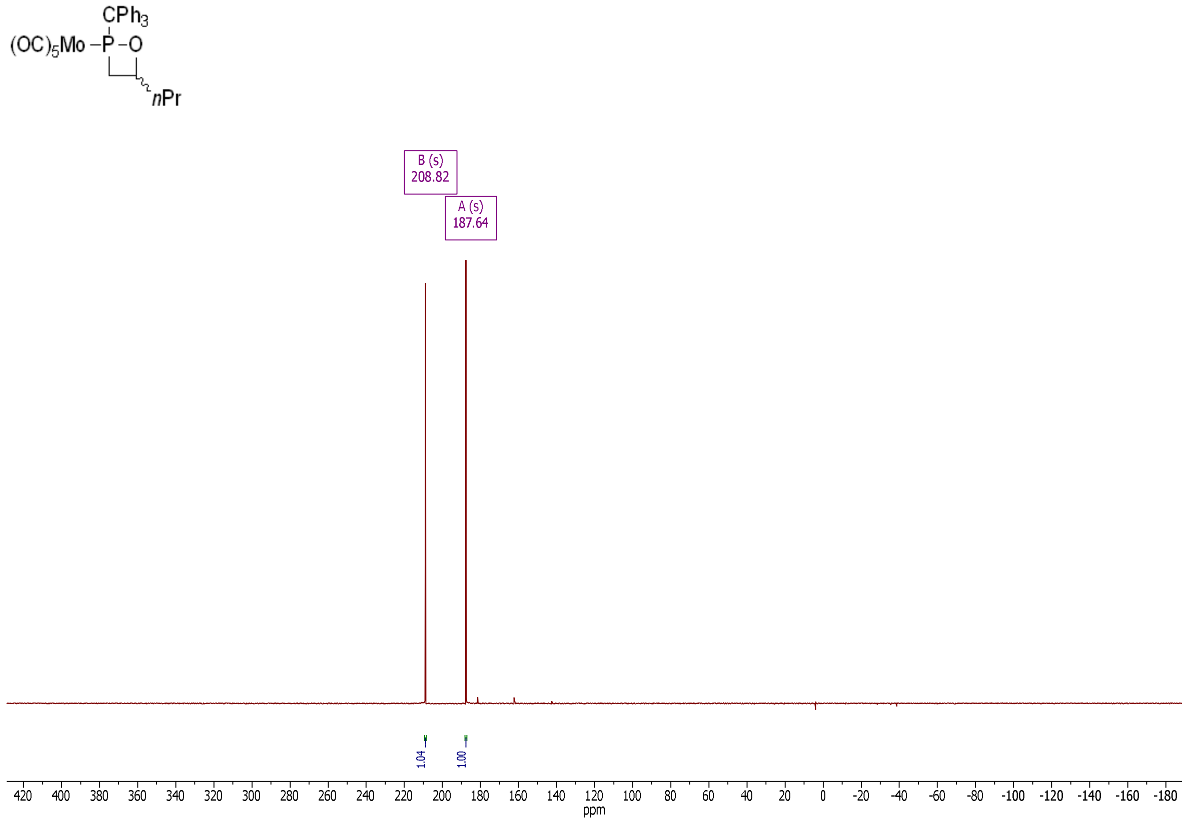

Figure S5: $^{31}P\{^{1}H\}$-NMR spectrum of **3c,c′** in $CDCl_3$.

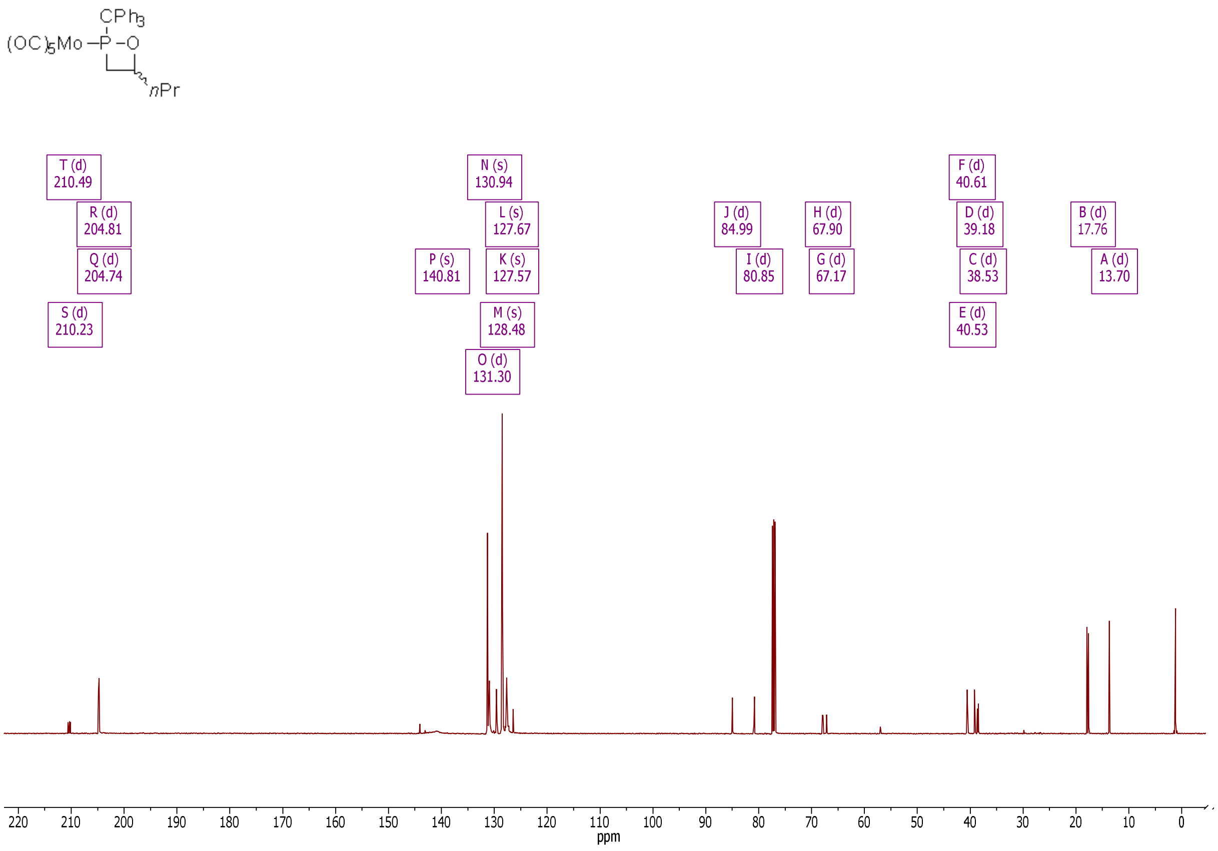

Figure S6: $^{13}C\{^1H\}$-NMR spectrum of **3c,c′** in $CDCl_3$.

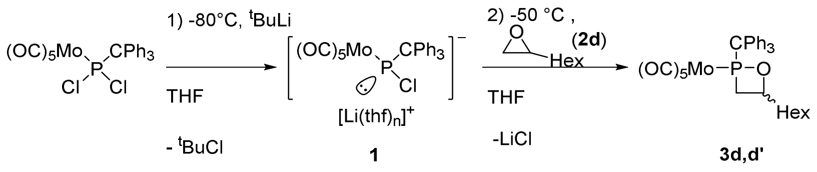

<u>Synthesis of complex **3d,d′**:</u> 0.58 g (1.00 mmol, 1 eq) of the Mo complex was dissolved in 20 ml of dried THF and cooled to -80°C. 0.7 mL (1.2 mmol, 1.2 eq) of a *tert*-butyllithium solution (1.7 M in *n*-pentane) was slowly added. The solution was kept stirring while slowly warming up. Upon reaching -50°C, 0.23 mL (1.5 mmol, 1.5 eq) 1,2-epoxyoctane (**2d**) were added. The solution was further kept stirring while slowly warming up to ambient temperature. After reaching room temperature, all volatiles were removed in vacuo (0.02 mbar). The crude product was purified by filtration with 150 mL of Et_2O over Al_2O_3 (h = 5 cm, d = 2 cm, r.t.) and removal of the solvent in vacuo (0.02 mbar). To remove impurities of diethyl ether, the product was dissolved in *n*-pentane and dried in vacuo (0.02 mbar). The product was obtained as a white solid.

Yield: 0.57 g, 0.90 mmol, 90%. Ratio (Isomer 1: Isomer 2) = 48:52. Melting point: decomposition above 120°C. **^{1}H NMR** (300.1 MHz, $CDCl_3$) δ (ppm) = **(Isomer 1)** 0.91 (m, 3H, -C*H*$_3$), 1.26 (m, 9H, -C*H*$_2$), 1.47 (m, 1H, -C*H*), 2.90 (m, 1H, -C*H*$_2$), 2.98 (m, 1H, -C*H*$_2$), 5.12 (m, 1H, -C*H*), 7.27-7.67 (m, 15H, -C*H*); **(Isomer 2)** 0.91 (m, 3H, -C*H*$_3$), 1.26 (m, 8H, -C*H*$_2$), 1.85 (m, 1H, -C*H*), 2.11 (m, 1H, -C*H*), 2.98 (m, 1H, -C*H*$_2$), 3.13 (m, 1H, -C*H*$_2$), 4.48 (m, 1H, -C*H*), 7.27-7.67 (m, 15H, -C*H*). **^{13}C NMR** (75.5 MHz, $CDCl_3$) δ (ppm) = **(Isomer 1)** 14.1 (s, 1C, -CH_3), 22.6 (s, 1C, -CH_2), 24.0 (s, 1C, -CH_2), 24.4 (s, 1C, -CH_2), 28.8 (d, 1C, $^4J_{P\text{-}C}$ = 1.6 Hz, -CH_2), 37.1 (d, 1C, $^3J_{P\text{-}C}$ = 2.6 Hz, -CH_2), 38.5 (d, 1C, $^1J_{P\text{-}C}$ = 21.5 Hz, -CH_2), 67.1 (d, 1C, $^1J_{P\text{-}C}$ = 9.4 Hz, -CPh_3), 81.1 (d, 1C, $^2J_{P\text{-}C}$ = 11.6 Hz, -CH), 127.5 (s, 1C, *para*-CH), 128.5 (s, 2C, *meta*-CH), 130.9 (d, 2C, $^3J_{P\text{-}C}$ = 5.9 Hz, *ortho*-CH), 140.7 (s_{br}, 1C, *ipso*-C), 204.7 (d, 4C, $^2J_{P\text{-}C}$ = 2.7 Hz, *cis*-CO), 210.1 (d, 1C, $^2J_{P\text{-}C}$ = 15.6 Hz, *trans*-CO); **(Isomer 2)** 14.1 (s, 1C, -CH_3), 22.6 (s, 1C, -CH_2), 24.0 (s, 1C, -CH_2), 24.4 (s, 1C, -CH_2), 31.7 (d, 1C, $^4J_{P\text{-}C}$ = 2.4 Hz, -CH_2), 38.5 (d, 1C, $^3J_{P\text{-}C}$ = 3.2 Hz, -CH_2), 40.5 (d, 1C, $^1J_{P\text{-}C}$ = 18.3 Hz, -CH_2), 67.9 (d, 1C, $^1J_{P\text{-}C}$ = 10.7 Hz, -CPh_3), 85.2 (d, 1C, $^2J_{P\text{-}C}$ = 11.5 Hz, -CH), 127.6 (s, 1C, *para*-CH), 128.5 (s, 2C, *meta*-CH), 131.3 (d, 2C, $^3J_{P\text{-}C}$ = 7.1 Hz, *ortho*-CH), 140.7 (s_{br}, 1C, *ipso*-C), 204.8 (d, 4C, $^2J_{P\text{-}C}$ = 2.8 Hz, *cis*-CO), 210.5 (d, 1C, $^2J_{P\text{-}C}$ = 15.9 Hz, *trans*-CO). **^{31}P NMR** (121.5 MHz, $CDCl_3$) δ (ppm) = **(Isomer 1)** 187.3 (d, $^3J_{P\text{-}H}$ = 11.1 Hz); **(Isomer 2)** 208.7 (d, $^3J_{P\text{-}H}$ = 10.0 Hz). **MS** (LIFDI) m/z (%) = 632.2 (50) $[M]^{\bullet+}$, 386.8 (10) $[M\text{-}CPh_3]^+$,243.2 (100) $[CPh_3]^+$. **HRMS** (EI) : theor./exp. 640.09123/640.09400 $[M]^+$. **IR ATR**: $\tilde{\nu}$ (cm^{-1}) = 2071 (s, ν(CO)), 1993 (m, ν(CO)), 1957 (s, ν(CO)), 1912 (s, ν(CO)).

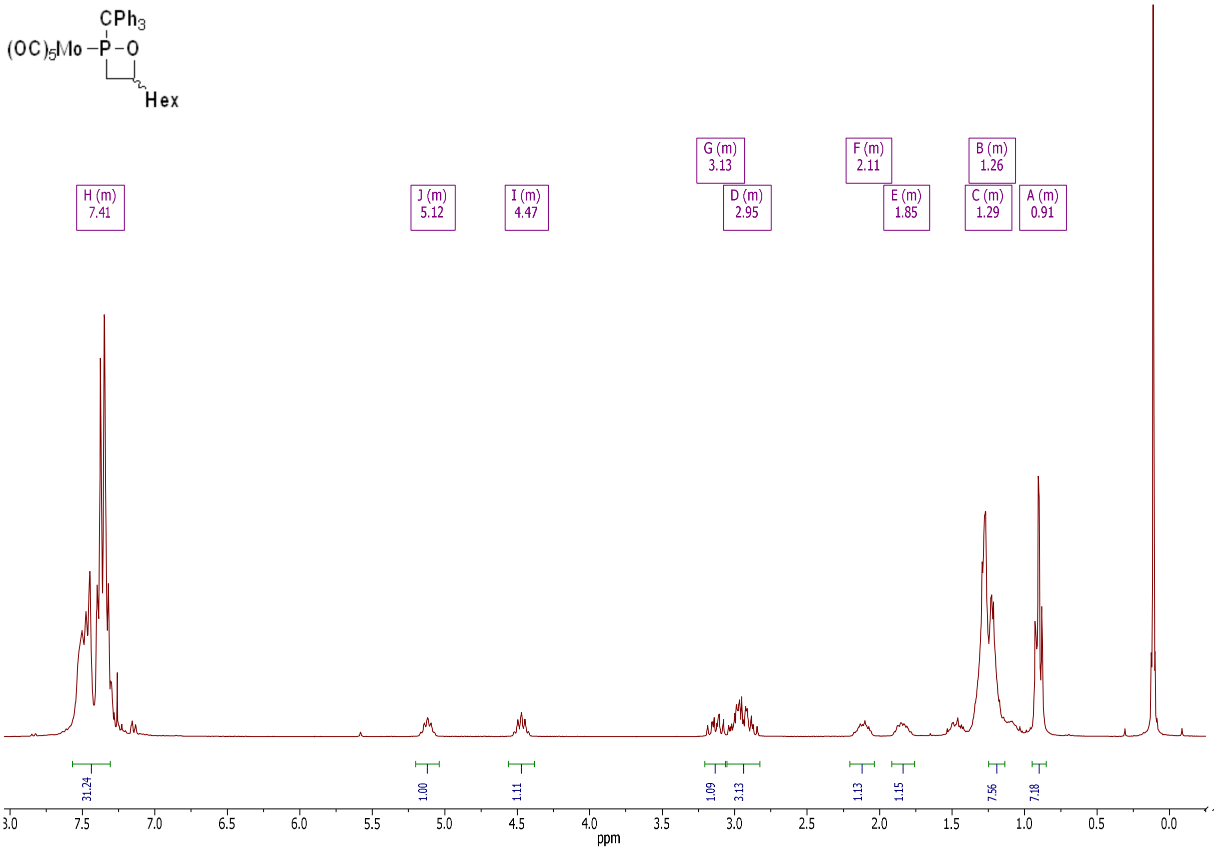

Figure S7: ^{1}H-NMR spectrum of **3d,d′ in** $CDCl_3$.

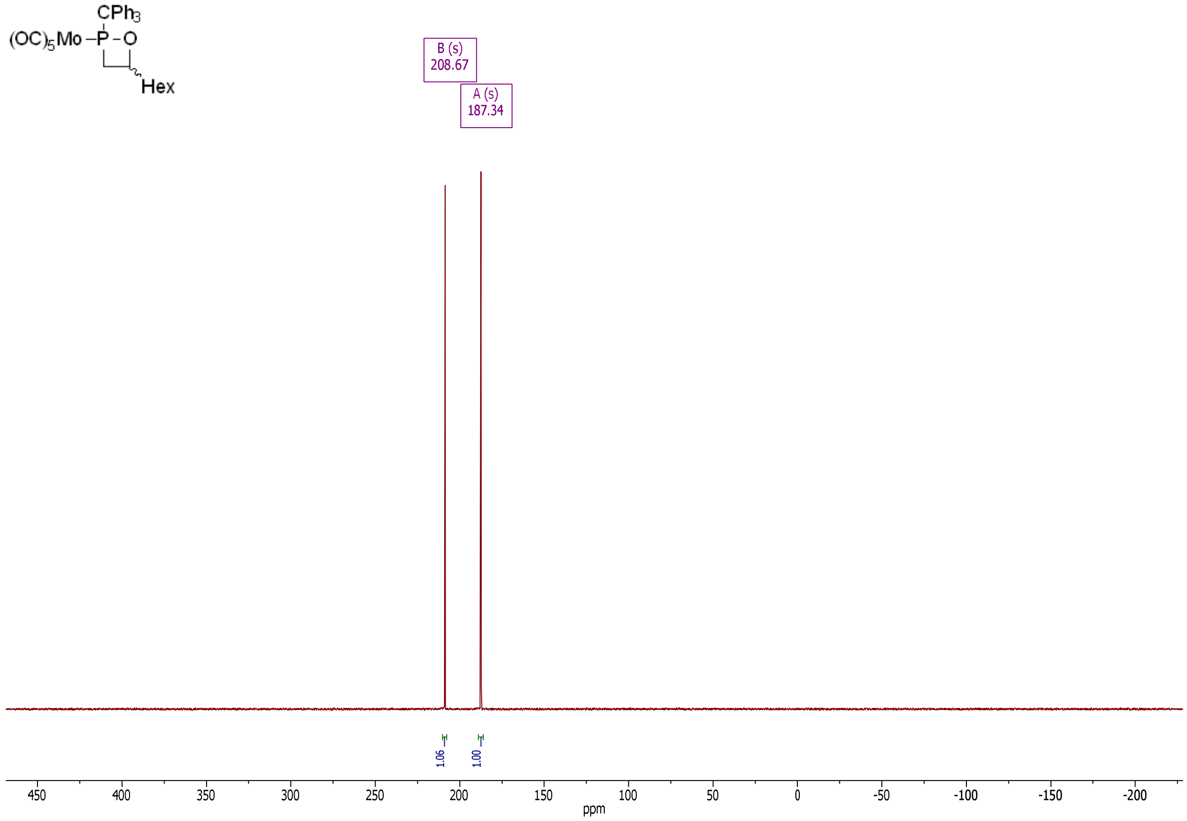

Figure S8: $^{31}P\{^{1}H\}$-NMR spectrum of **3d,d′** in $CDCl_3$.

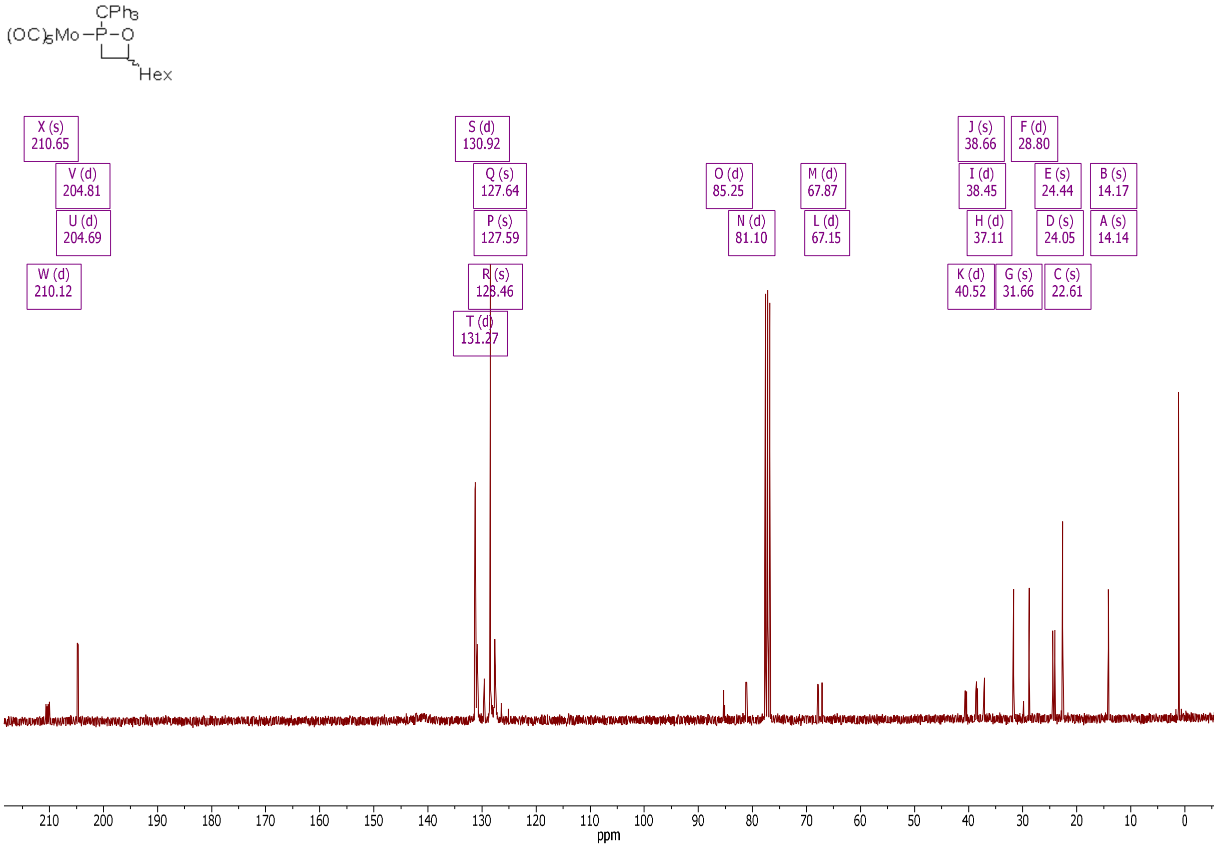

Figure S9: $^{13}C\{^{1}H\}$-NMR spectrum of **3d,d′** in $CDCl_3$.

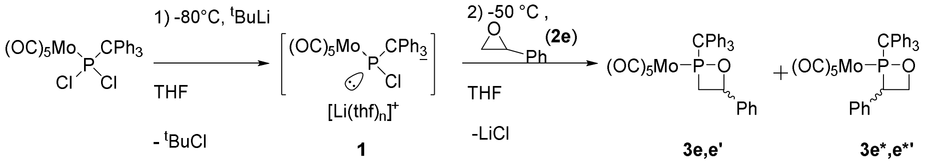

<u>Synthesis of complex **3e,e′** and **3e*,e*′**:</u> 0.17 g (0.30 mmol, 1 eq) of the Mo complex was dissolved in 20 ml of dried THF and cooled to -80°C. 0.18 mL (0.36 mmol, 1.2 eq) of a *tert*-butyllithium solution (1.7 M in *n*-pentane) was slowly added. The solution was kept stirring while slowly warming up. Upon reaching -50°C, 0.11 mL (0.90 mmol, 3.0 eq) styrene oxide (**2e**) were added. The solution was further kept stirring while slowly warming up to ambient temperature. After reaching room temperature, all volatiles were removed in vacuo (0.02 mbar). The crude product was purified by filtration with 250 mL of Et_2O over Al_2O_3 (h = 7 cm, d = 2 cm, r.t.) and removal of the solvent in vacuo (0.02 mbar). To remove impurities of diethyl ether, the product was dissolved in *n*-pentane and dried in vacuo (0.02 mbar). The crude product mixture was obtained as a brown solid.

Yield: 0.18 g, 0.28 mmol, 93% (crude product). **Ratio (3e:3e′:3e*:3e*′)** = 40:45:10:5. **^{31}P NMR** (202.5 MHz, $CDCl_3$) δ (ppm) = **3e,e′: (Isomer 1)** 191.5 (dd, $^{2}J_{P-H}$ = 13.7 Hz, $^{2}J_{P-H}$ = 14.4 Hz); **(Isomer 2)** 210.9 (d, $^{2}J_{P-H}$ = 10.3 Hz). **3e*,e*′: (Isomer 1)** 237.3 (d, $^{2}J_{P-H}$ = 17.7 Hz); **(Isomer 2)** 244.9 (s_{br}). **MS** (LIFDI) m/z (%) = 632.2 (50) $[M]^{\bullet+}$, 386.8 (10) $[M-CPh_3]^+$,243.2 (100) $[CPh_3]^+$.

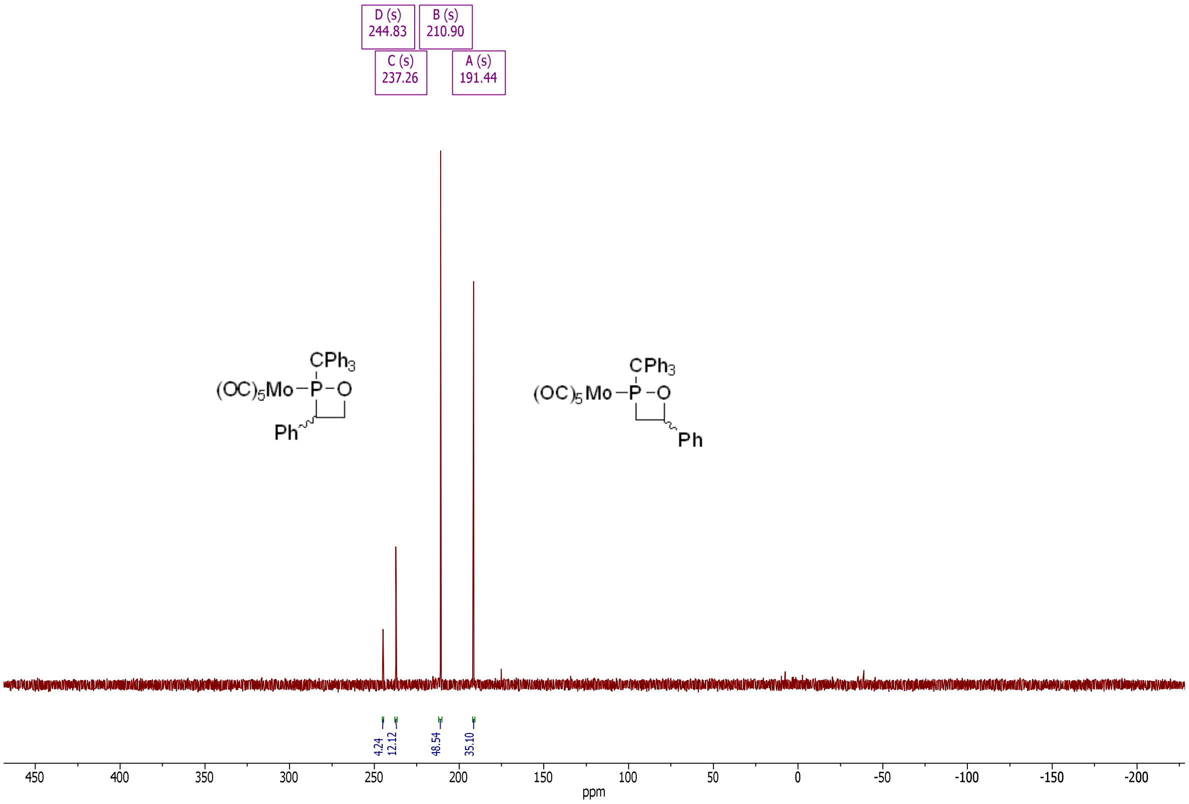

Figure S10: $^{31}P\{^{1}H\}$-NMR spectrum of **3e,e′** and **3e*,e*′** in $CDCl_3$.

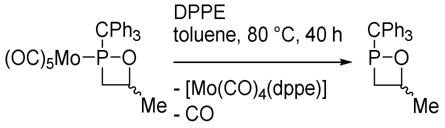

3a,a' **4a,a'**

Synthesis of **4a,a′**: 1705.6 mg (3 mmol, 1 eq) of complexes **3a,a′** and 977.2mg (2.94 mmol, 0.98 eq) 1,2-bis(diphenylphosphino)ethane (DPPE) were dissolved in 20 mL toluene and heated to 80°C for 40 h. Full conversion was proven by ^{31}P NMR measurement. The product was obtained after extraction with *n*-pentane (one time 20 mL, four times 10 mL, ambient temperature). The obtained analytical data are in accordance with the known literature [1].

Yield: 743.0 mg, 2.24 mmol, 75%. Ratio (Isomer 1: Isomer 2) = 39:61. **$^{31}P\{^{1}H\}$ NMR** (202.5 MHz, $CDCl_3$) δ (ppm)= **(Isomer 1)** 163.7 (s); **(Isomer 2)** 199.0 (s).

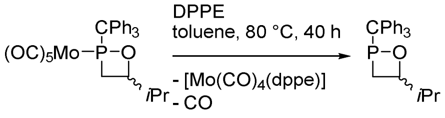

3b,b' **4b,b'**

Synthesis of **4b,b′**: 179.0 mg (0.3 mmol, 1 eq) of complexes **3b,b′** and 115.5 mg (0.27 mmol, 0.9 eq) 1,2-bis(diphenylphosphino)ethane (DPPE) were dissolved in 6 mL toluene and heated to 80°C for 40 h. Full conversion was proven by ^{31}P NMR measurement. The product was obtained after extraction with *n*-pentane (three times 2 mL, ambient temperature).

Yield: 75.5 mg, 0.21 mmol, 70%. Ratio (Isomer 1: Isomer 2) = 34:66. **^{1}H NMR** (300.1 MHz, $CDCl_3$) δ (ppm) = **(Isomer 1)** 0.44 (d, 3H, $^{3}J_{H-H}$ = 6.7 Hz, -C*H*$_3$), 0.79 (d, 3H, $^{3}J_{H-H}$ = 6.5 Hz, -C*H*$_3$), 1.30 (m, 1H, -C*H*), 2.16 (ddd, 1H, $^{2}J_{H-H}$ = 11.7 Hz, $^{3}J_{H-H}$ = 8.9 Hz, $^{3}J_{P-H}$ = 2.6 Hz, -C*H*$_2$), 2.66 (m, 1H, -C*H*$_2$), 4.36 (ddd, 1H, $^{3}J_{H-H}$ = 10.1 Hz, $^{3}J_{H-H}$ = 8.9 Hz, $^{3}J_{H-H}$ = 6.4 Hz, -C*H*), 7.22-7.32 (m, 15H, -C*H*); **(Isomer 2)** 0.83 (d, 3H, $^{3}J_{H-H}$ = 5.0 Hz, -C*H*$_3$), 0.86 (d, 3H, $^{3}J_{H-H}$ = 4.9 Hz, -C*H*$_3$), 1.93 (m, 1H, -C*H*), 2.29 (ddd, 1H, $^{2}J_{H-H}$ = 12.8 Hz, $^{3}J_{H-H}$ = 9.1 Hz, $^{3}J_{P-H}$ = 3.4 Hz, -C*H*$_2$), 2.53 (m, 1H, -C*H*$_2$), 4.13 (dddd, 1H, $^{3}J_{H-H}$ = 8.9 Hz, $^{3}J_{H-H}$ = 7.3 Hz, $^{3}J_{H-H}$ = 5.8 Hz, $^{3}J_{P-H}$ = 1.8 Hz, -C*H*), 7.22-7.32 (m, 15H, -C*H*). **^{13}C NMR** (75.5 MHz, $CDCl_3$) δ (ppm) = **(Isomer 1)** 15.6 (s, 1C, -CH$_3$), 17.6 (s, 1C, -CH$_3$), 31.6 (d, 1C, $^{1}J_{P-C}$ = 12.8 Hz, -CH$_2$), 35.9 (d, 1C, $^{3}J_{P-C}$ = 2.3 Hz, -CH), 63.4 (d, 1C, $^{1}J_{P-C}$ = 52.6 Hz, -CPh$_3$), 86.3 (d, 1C, $^{2}J_{P-C}$ = 4.2 Hz, -CH), 126.8 (s, 1C, $^{5}J_{P-C}$ = 1.5 Hz, *para*-CH), 128.3 (s, 2C, *meta*-CH), 130.3 (d, 2C, $^{3}J_{P-C}$ = 9.5 Hz, *ortho*-CH), 142.7 (d, 1C, $^{2}J_{P-C}$ = 8.7 Hz, *ipso*-*C*); **(Isomer 2)** 14.2 (s, 1C, -CH$_3$), 22.5 (s, 1C, -CH$_3$), 26.9 (d, 1C, $^{1}J_{P-C}$ = 7.5 Hz, -CH$_2$), 35.5 (s$_{br}$, 1C, -CH), 63.1 (d, 1C, $^{1}J_{P-C}$ = 51.0 Hz, -CPh$_3$), 90.8 (d, 1C, $^{2}J_{P-C}$ = 1.9 Hz, -CH), 126.7 (s, 1C, $^{5}J_{P-C}$ = 1.4 Hz, *para*-CH), 128.4 (s, 2C, *meta*-CH), 130.0 (d, 2C, $^{3}J_{P-C}$ = 9.5 Hz, *ortho*-CH), 143.5 (d, 1C, $^{2}J_{P-C}$ = 8.7 Hz, *ipso*-*C*). **^{31}P{^{1}H} NMR** (121.5 MHz, $CDCl_3$) δ (ppm) = **(Isomer 1)** 161.2 (s); **(Isomer 2)** 196.7 (s). **MS** (LIFDI) m/z (%) = 360.1 (95) [M]$^{•+}$, 243.1 (100) [CPh$_3$]$^{+}$. **HRMS** (EI): theor./exp. 360.16430/360.16333 [M]$^{+}$.

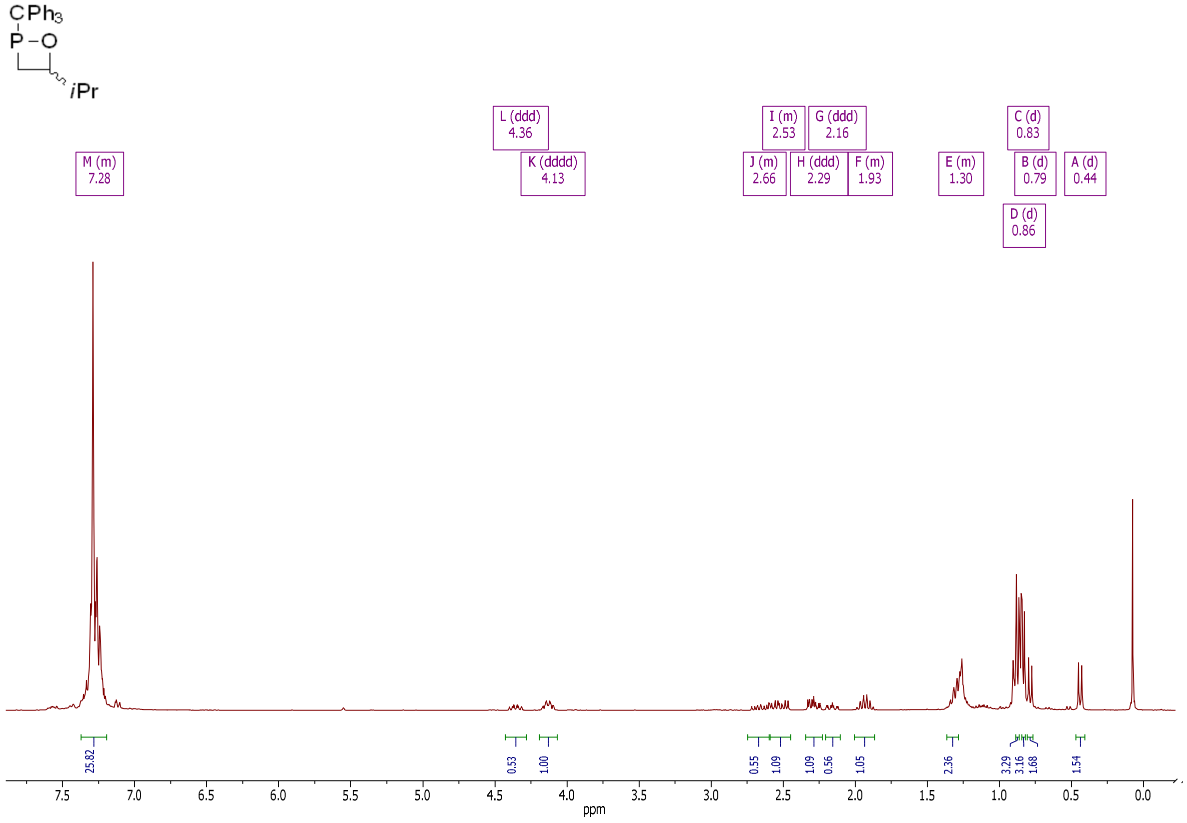

Figure S11: ^{1}H-NMR spectrum of **4b,b'** in $CDCl_3$.

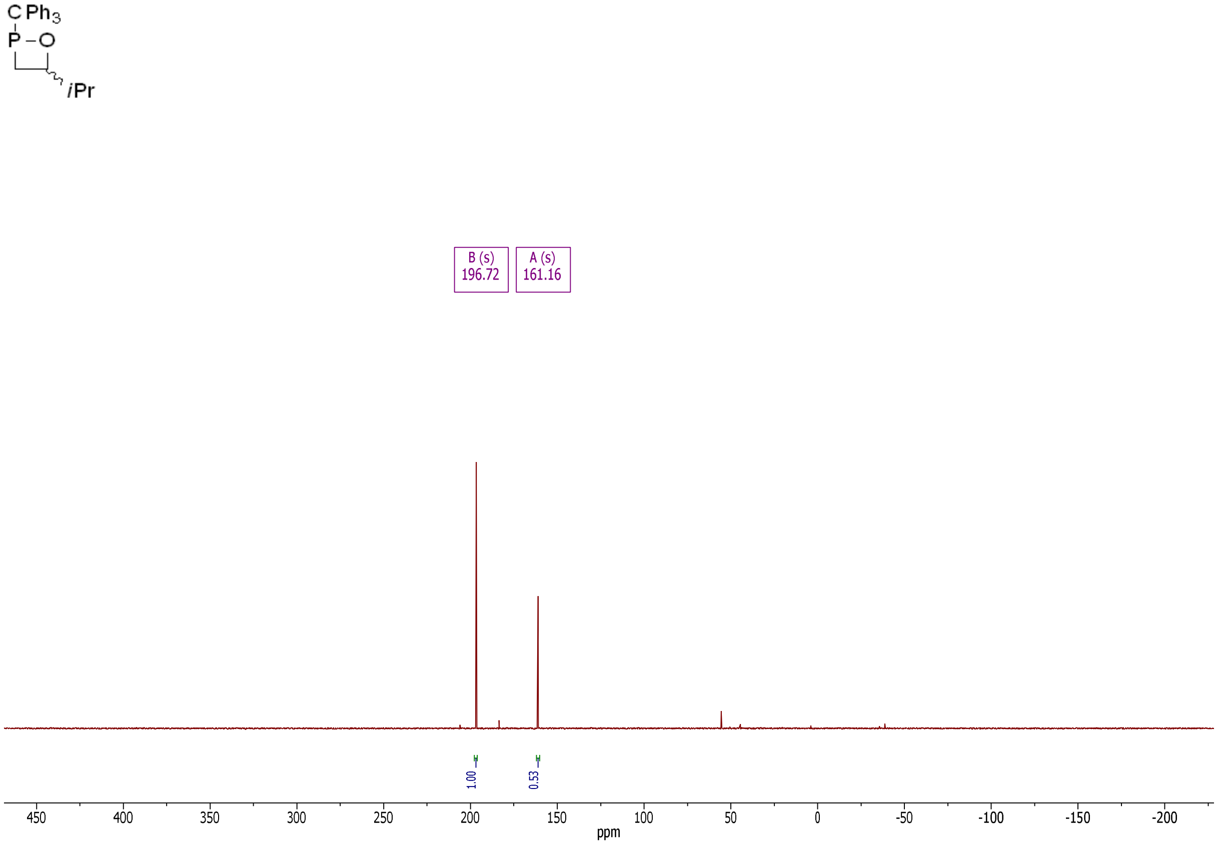

Figure S12: $^{31}P\{^{1}H\}$-NMR spectrum of **4b,b′** in $CDCl_3$.

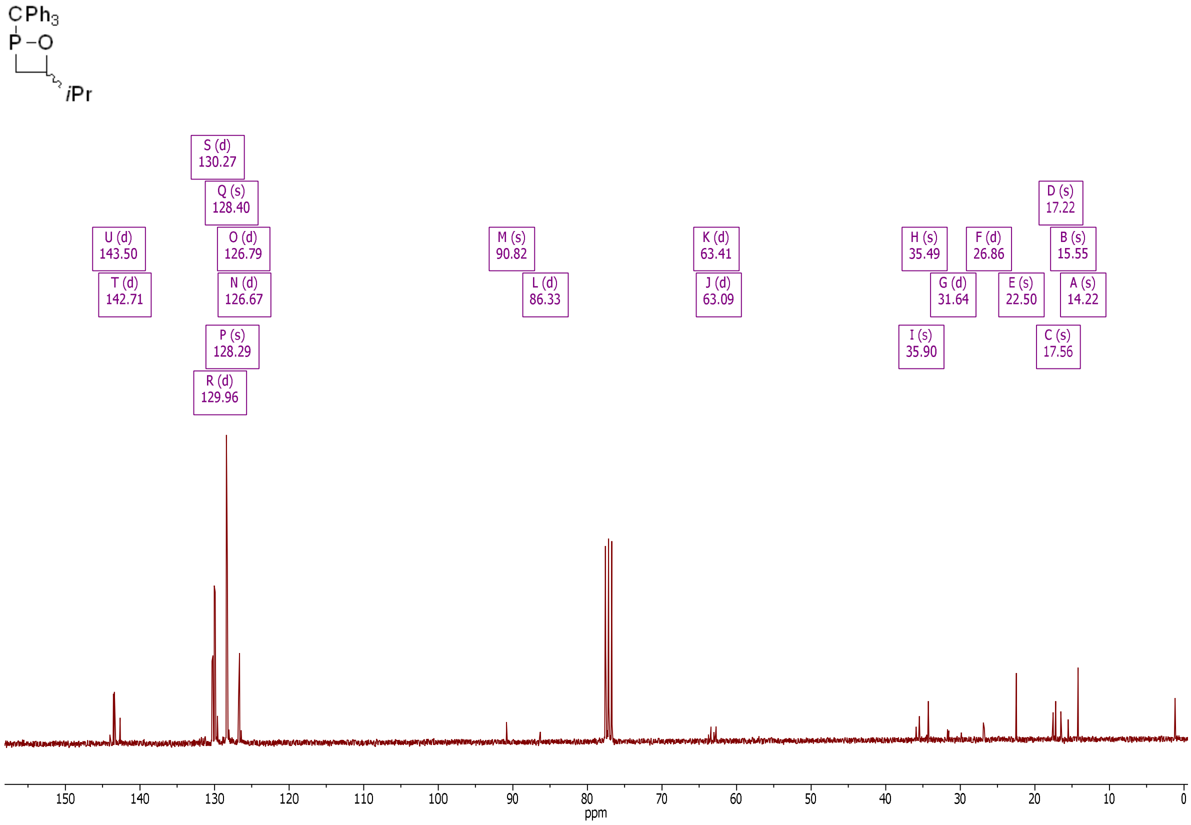

Figure S13: $^{13}C\{^{1}H\}$-NMR spectrum of **4b,b′** in $CDCl_3$.

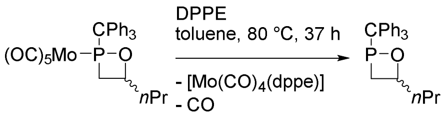

3c,c' **4c,c'**

Synthesis of **4c,c'**: 119.5 mg (0.2 mmol, 1 eq) of complexes **3c,c'** and 71.5 mg (0.18 mmol, 0.9 eq) 1,2-bis(diphenylphosphino)ethane (DPPE) were dissolved in 6 mL toluene and heated to 80°C for 37 h. Full conversion was proven by ^{31}P NMR measurement. The product was obtained after extraction with *n*-pentane at ambient temperature (3 times 3 mL). After evaporation of solvent (atmospheric pressure, ambient temperature), the product was obtained as yellow crystals.

Yield: 68.9 mg, 0.19 mmol, 96%. Ratio (Isomer 1: Isomer 2) = 28:72. **^{1}H NMR** (500.1 MHz, $CDCl_3$) δ (ppm) = **(Isomer 1)** 0.79 (t, 3H, $^{3}J_{H-H}$ = 7.5 Hz, -C*H*3), 1.00 (m, 1H, -C*H*2), 1.06 (m, 1H, -C*H*2), 1.29 (m, 2H, -C*H*2), 2.20 (m, 1H, -C*H*2), 2.68 (m, 1H, -C*H*2), 4.95 (m, 1H, -C*H*), 7.23-7.36 (m, 15H, -C*H*); **(Isomer 2)** 0.86 (t, 3H, $^{3}J_{H-H}$ = 7.4 Hz, -C*H*3), 1.29 (m, 2H, -C*H*2), 1.67 (m, 1H, -C*H*2), 1.84 (m, 1H, -C*H*2), 2.38 (m, 1H, -C*H*2), 2.51 (m, 1H, -C*H*2), 4.45 (m, 1H, -C*H*), 7.23-7.36 (m, 15H, -C*H*). **^{13}C NMR** (125.8 MHz, $CDCl_3$) δ (ppm) = **(Isomer 1)** 13.8 (s, 1C, -CH_3), 17.2 (s, 1C, -CH_2), 32.4 (d, 1C, $^{1}J_{P-C}$ = 12.8 Hz, -CH_2), 40.7 (d, 1C, $^{3}J_{P-C}$ = 2.5 Hz, -CH_2-CH_2), 63.4 (d, 1C, $^{1}J_{P-C}$ = 52.6 Hz, -CPh_3), 81.0 (d, 1C, $^{2}J_{P-C}$ = 4.4 Hz, -CH), 126.8 (s, 1C, $^{5}J_{P-C}$ = 1.6 Hz, *para*-CH), 128.3 (s, 2C, *meta*-CH), 130.3 (d, 2C, $^{3}J_{P-C}$ = 9.3 Hz, *ortho*-CH), 142.8 (d, 1C, $^{2}J_{P-C}$ = 8.7 Hz, *ipso*-*C*); **(Isomer 2)** 13.9 (s, 1C, -CH_3), 17.5 (s, 1C, -CH_2), 29.5 (d, 1C, $^{1}J_{P-C}$ = 7.9 Hz, -CH_2), 41.9 (s, 1C, -CH_2-CH_2), 63.0 (d, 1C, $^{1}J_{P-C}$ = 51.0 Hz, -CPh_3), 86.1 (d, 1C, $^{2}J_{P-C}$ = 2.5 Hz, -CH), 126.7 (s, 1C, $^{5}J_{P-C}$ = 1.4 Hz, *para*-CH), 128.3 (s, 2C, *meta*-CH), 129.9 (d, 2C, $^{3}J_{P-C}$ = 9.8 Hz, *ortho*-CH), 143.5 (d, 1C, $^{2}J_{P-C}$ = 8.5 Hz, *ipso*-*C*). **^{31}NMR** (202.5 MHz, CDCL3) δ (ppm) = (**Isomer 1**) 166.7 (d, $^{2}J_{P-H}$ = 19.6 Hz); (**Isomer 2**) 199.6 (d, $^{2}J_{P-H}$ = 22.4 Hz). **MS** (ESI+) m/z (%) : 243.1 (100) $[CPh_3]^+$, 361.2 (>1) $[M+H]^+$, 377.2 (8) $[M+O+H]^+$, 379.2 (12) $[M+H_2O+H]^+$. **HRMS** (ESI+) : theor./exp. 361.1716/361.1719 $[M+H]^+$.

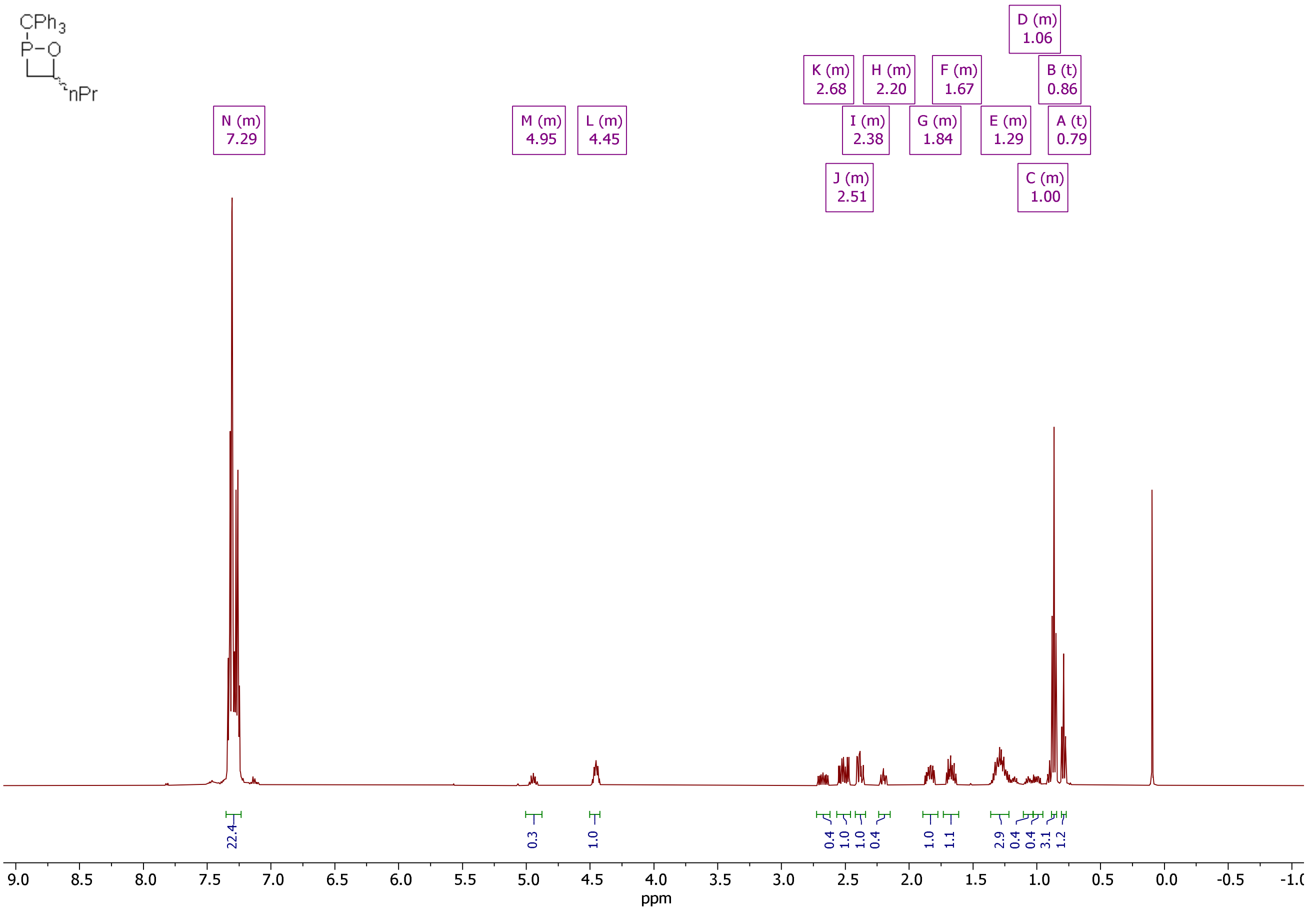

Figure S14: ^{1}H-NMR spectrum of **4c,c'** in $CDCl_3$.

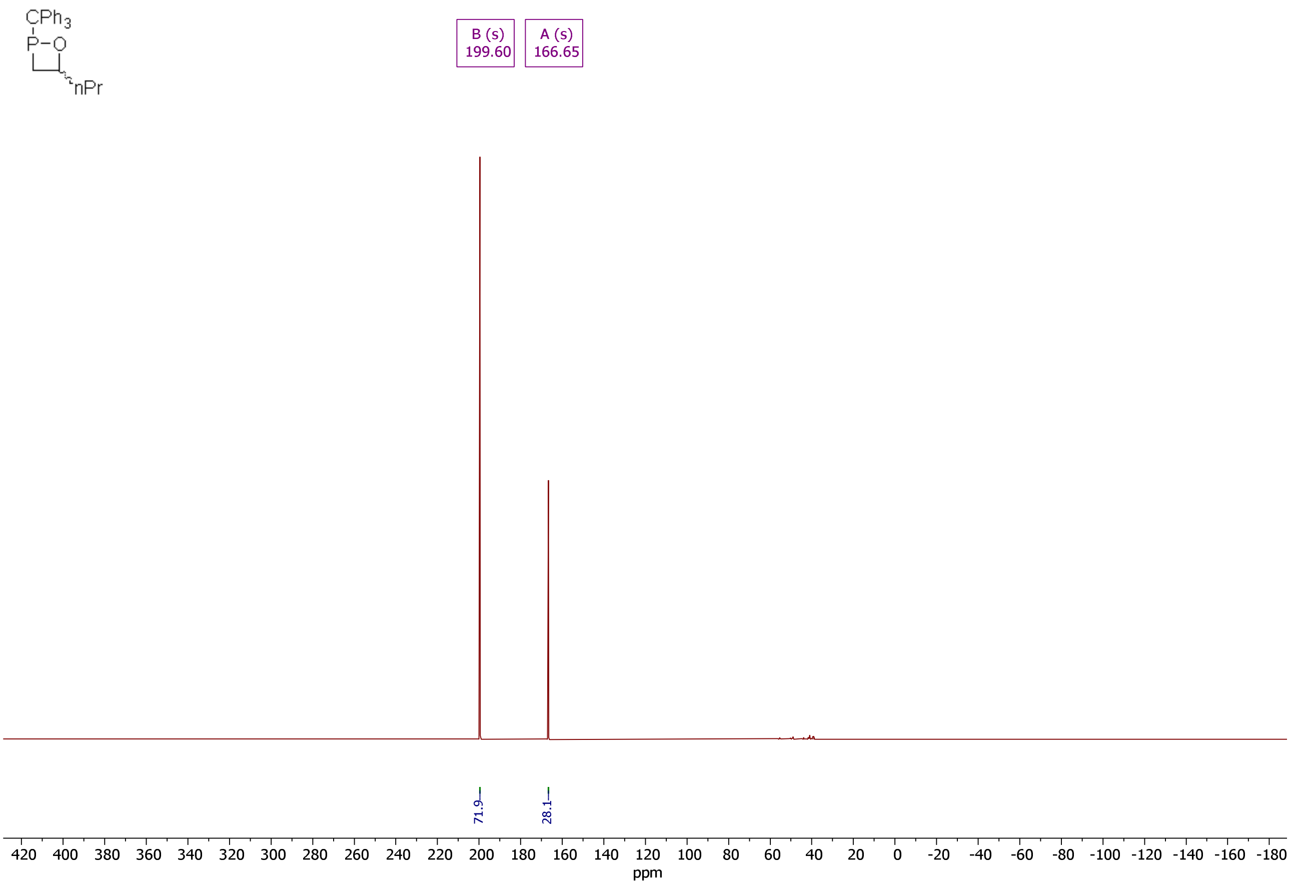

Figure S15: $^{31}P\{^{1}H\}$-NMR spectrum of **4c,c'** in $CDCl_3$.

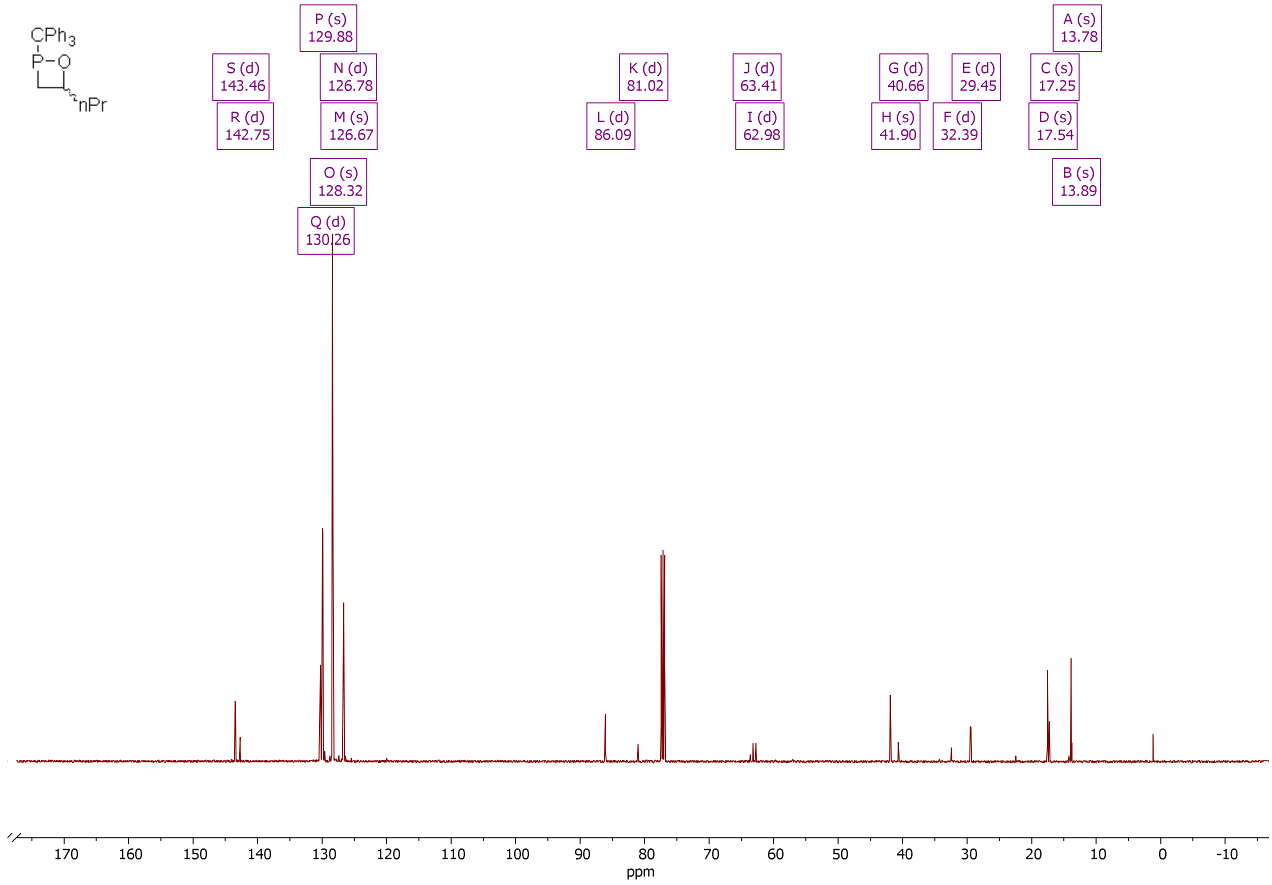

Figure S16: $^{13}C\{^{1}H\}$-NMR spectrum of **4c,c'** in $CDCl_3$.

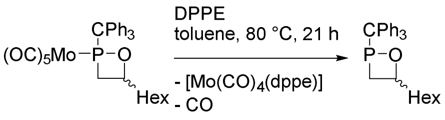

3d,d' **4d,d'**

Synthesis of **4d,d'**: 383.0 mg (0.6 mmol, 1 eq) of complexes **3d,d'** and 231.9 mg (0.58 mmol, 0.97 eq) 1,2-bis(diphenylphosphino)ethane (DPPE) were dissolved in 6 mL toluene and heated to 80°C for 21 h. Full conversion was proven by ^{31}P NMR measurement. The product was obtained after extraction with *n*-pentane (three times 2 mL, ambient temperature).

Ratio (Isomer 1: Isomer 2) = 45:55. **^{1}H NMR** (500.1 MHz, $CDCl_3$) δ (ppm) = **(Isomer 1)** 0.93 (t, 3H, $^{3}J_{H\text{-}H}$ = 7.0 Hz, -C*H*$_3$), 1.08 (m, 1H, -C*H*$_2$), 1.18-1.26 (m, 3H, -C*H*$_2$), 1.28-1.36 (m, 5H, -C*H*$_2$), 2.26 (ddd, 1H, $^{2}J_{H\text{-}H}$ = 11.8 Hz, $^{3}J_{H\text{-}H}$ = 8.9 Hz, $^{2}J_{P\text{-}H}$ = 2.6 Hz,-C*H*$_2$), 2.72 (ddd, 1H, $^{2}J_{P\text{-}H}$ = 19.5 Hz, $^{2}J_{H\text{-}H}$ = 12.0 Hz, $^{3}J_{H\text{-}H}$ = 6.5 Hz, -C*H*$_2$), 4.98 (dddd, 1H, $^{3}J_{H\text{-}H}$ = 8.9 Hz, $^{3}J_{H\text{-}H}$ = 7.7 Hz, $^{3}J_{H\text{-}H}$ = 6.4 Hz, $^{3}J_{H\text{-}H}$ = 6.4 Hz, -C*H*), 7.30-7.38 (m, 15H, -C*H*); **(Isomer 2)** 0.94 (t, 3H, $^{3}J_{H\text{-}H}$ = 7.2 Hz, -C*H*$_3$), 1.08 (m, 1H, -C*H*$_2$), 1.18-1.26 (m, 3H, -C*H*$_2$), 1.28-1.36 (m, 4H, -C*H*$_2$), 1.75 (m, 1H, -C*H*$_2$), 1.90 (m, 1H, -C*H*$_2$), 2.44 (ddd, 1H, $^{2}J_{H\text{-}H}$ = 13.0 Hz, $^{3}J_{H\text{-}H}$ = 8.9 Hz, $^{2}J_{P\text{-}H}$ = 3.0 Hz,-C*H*$_2$), 2.57 (ddd, 1H, $^{2}J_{P\text{-}H}$ = 21.2 Hz, $^{2}J_{H\text{-}H}$ = 13.0 Hz, $^{3}J_{H\text{-}H}$ = 5.6 Hz, -C*H*$_2$), 4.51 (m, 1H, -C*H*), 7.30-7.38 (m, 15H, -C*H*). **^{13}C NMR** (125.8 MHz, $CDCl_3$) δ (ppm) = **(Isomer 1)** 14.2 (s, 1C, -CH$_3$), 22.6 (s, 1C, -CH$_2$), 23.8 (s, 1C, -CH$_2$), 28.9 (s, 1C, -CH$_2$), 31.7 (s, 1C, -CH$_2$), 32.3 (d, 1C, $^{1}J_{P\text{-}C}$ = 13.1 Hz, -CH$_2$), 38.5 (d, 1C, $^{3}J_{P\text{-}C}$ = 2.7 Hz, -CH$_2$), 63.4 (d, 1C, $^{1}J_{P\text{-}C}$ = 52.6 Hz, -CPh$_3$), 81.2 (d, 1C, $^{2}J_{P\text{-}C}$ = 4.4 Hz, -CH), 126.7 (s, 1C, $^{5}J_{P\text{-}C}$ = 1.6 Hz, *para*-CH), 128.3 (s, 2C, *meta*-CH), 130.2 (d, 2C, $^{3}J_{P\text{-}C}$ = 9.5 Hz, *ortho*-CH), 142.7 (d, 1C, $^{2}J_{P\text{-}C}$ = 8.7 Hz, *ipso*-C); **(Isomer 2)** 14.1 (s, 1C, -CH$_3$), 22.6 (s, 1C, -CH$_2$), 24.2 (s, 1C, -CH$_2$), 29.1 (s, 1C, -CH$_2$), 29,4 (d, 1C, $^{1}J_{P\text{-}C}$ = 7.6 Hz, -CH$_2$),31.8 (s, 1C, -CH$_2$), 39.8 (s, 1C, -CH$_2$), 62.9 (d, 1C, $^{1}J_{P\text{-}C}$ = 51.0 Hz, -CPh$_3$), 86.3 (d, 1C, $^{2}J_{P\text{-}C}$ = 2.2 Hz, -CH), 126.6 (s, 1C, $^{5}J_{P\text{-}C}$ = 1.4 Hz, *para*-CH), 128.3 (s, 2C, *meta*-CH), 129.9 (d, 2C, $^{3}J_{P\text{-}C}$ = 9.5 Hz, *ortho*-CH), 143.4 (d, 1C, $^{2}J_{P\text{-}C}$ = 8.7 Hz, *ipso*-C). **^{31}P NMR** (202.5 MHz, $CDCl_3$) δ (ppm) = (**Isomer 1**) 166.4 (d, $^{2}J_{P\text{-}H}$ = 19.5 Hz); (**Isomer 2**) 199.4 (d, $^{2}J_{P\text{-}H}$ = 21.9 Hz). **MS** (EI) m/z (%): 165.1 (32) $[C_{13}H_9]^+$, 243.1 (100) $[CPh_3]^+$, 402.2 (>>1) $[M]^{\bullet+}$. **HRMS** (EI): theor./exp. 402.211253/402.20989 $[M]^+$.

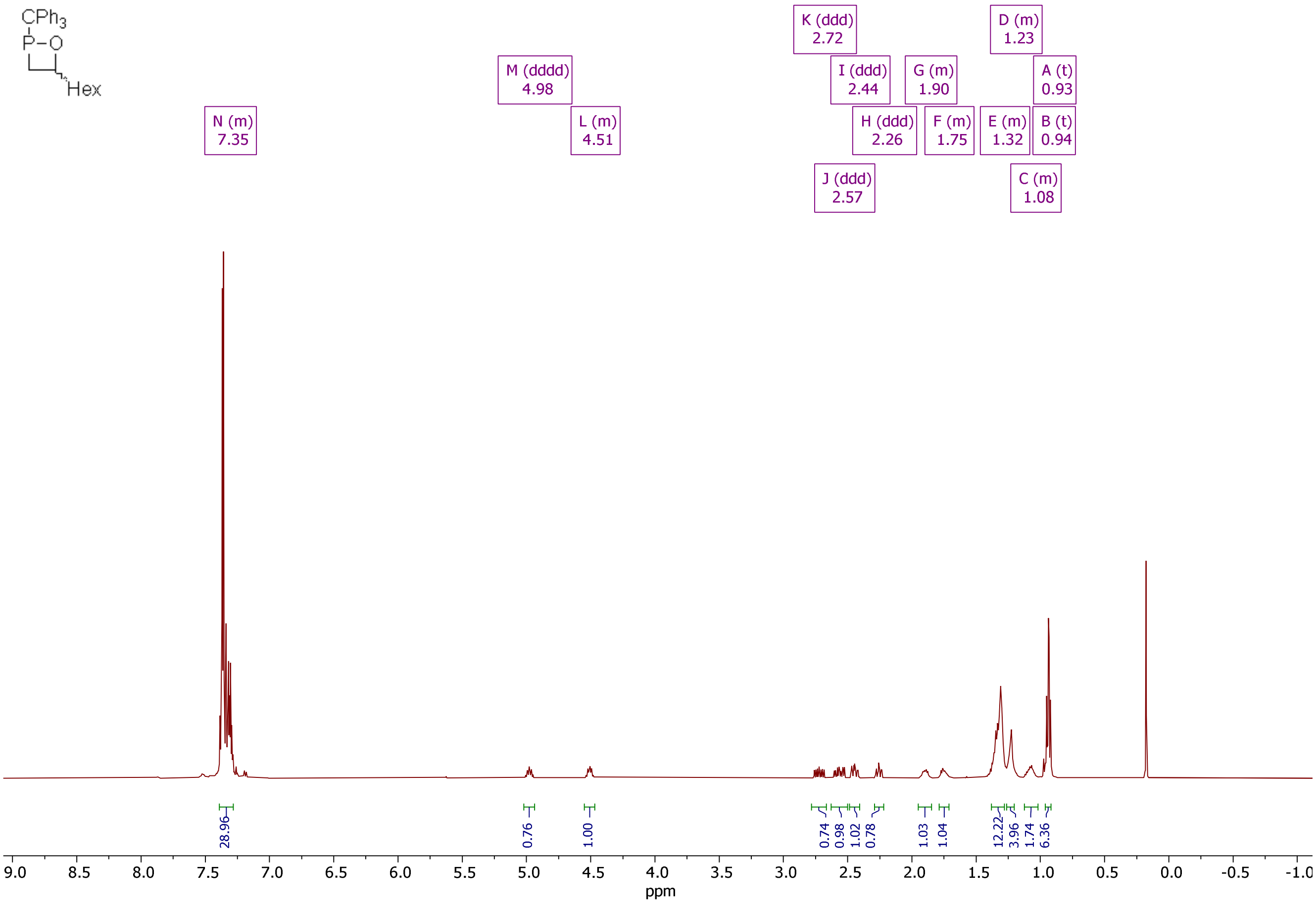

Figure S17: ^{1}H-NMR spectrum of **4d,d'** in $CDCl_3$.

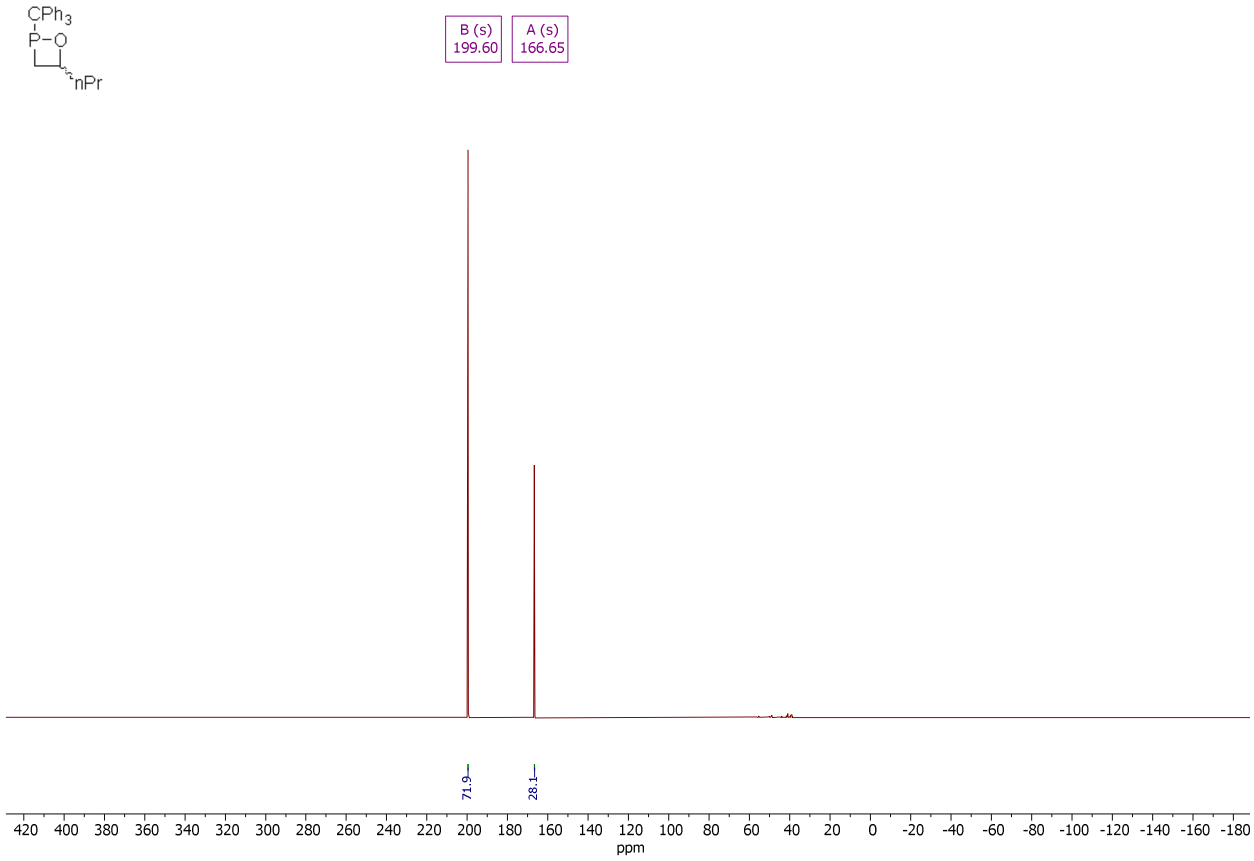

Figure S18: $^{31}P\{^{1}H\}$-NMR spectrum of **4d,d′** in $CDCl_3$.

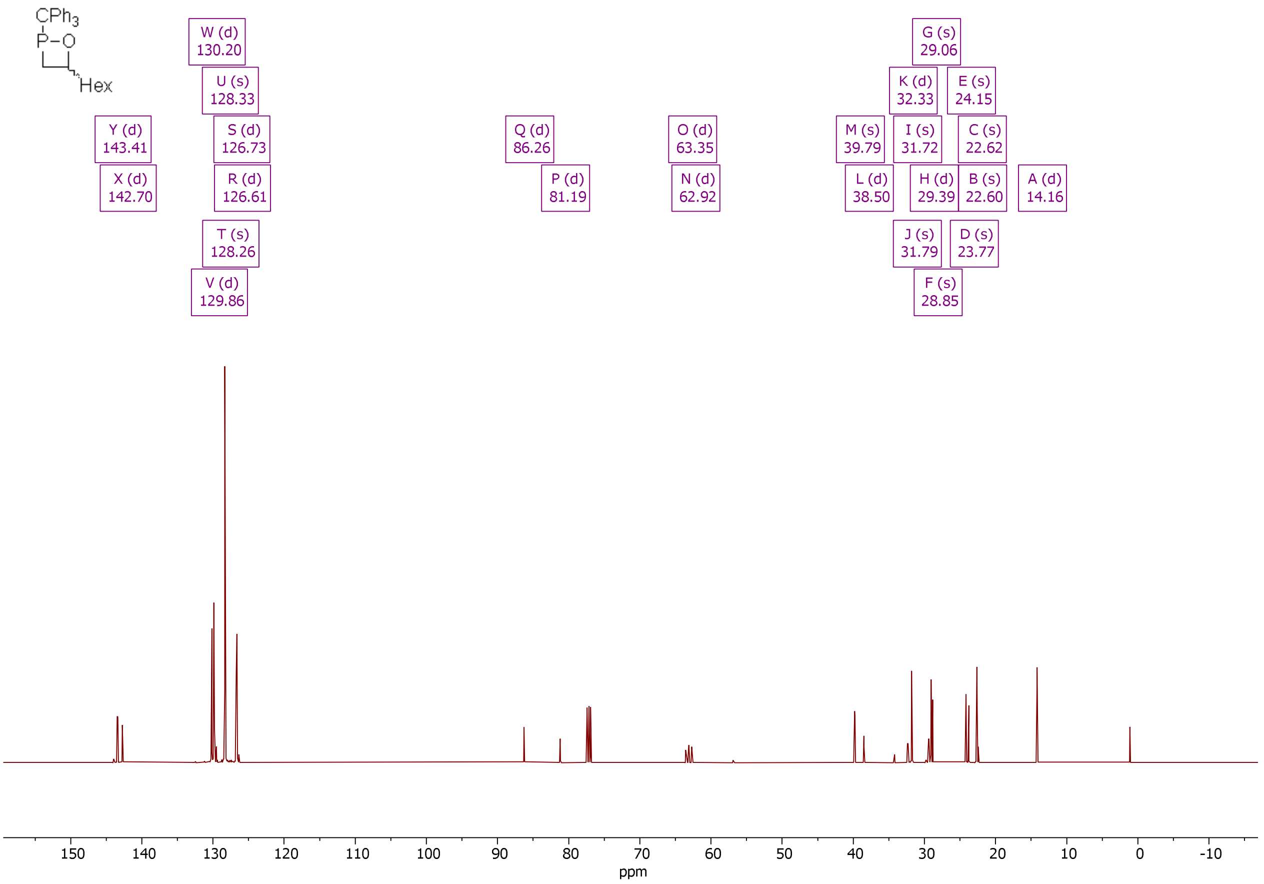

Figure S19: $^{13}C\{^1H\}$-NMR spectrum of **4d,d′** in $CDCl_3$.

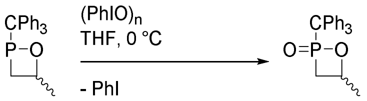

4a,a' **14a,a'**

<u>Synthesis of **14a,a′:**</u> 166.2 mg **4a,a′** (0.5 mmol, 1 eq) was dissolved in 15 mL THF and cooled to 0°C. 132.0 mg (0.6 mmol, 1.2 eq) iodosylbenzene ($(PhIO)_n$) was added to the cooled solution. The reaction mixture was stirred at 0°C for 28 h. The solvent was then removed in vacuo (0.02 mbar) at 0°C. The crude product was washed at 0°C; twice with a mixture of 5 mL *n*-pentane and 1 mL diethyl ether, then twice with 5 mL pure *n*-pentane. After removal of solvent in vacuo (0.02 mbar) at 0°C, the product was obtained as a white solid.

Yield: 86.3 mg, 0.25 mmol, 50%. Ratio (Isomer 1: Isomer 2) = 66:34. **^{1}H NMR** (500 MHz, 298 K, $CDCl_3$): δ / ppm = (**Isomer 1**) 1.52 (d, 3H, $^3J_{H\text{-}H}$= 6.2 Hz, -C*H*$_3$), 2.53 (m, 1H, -C*H*$_2$), 2.75 (ddd, 1H, $^2J_{H\text{-}H}$= 14.5 Hz, $^2J_{P\text{-}H}$= 9.7 Hz, $^3J_{H\text{-}H}$= 7.8 Hz, -C*H*$_2$), 4.25 (m, 1H, -C*H*), 7.28-7.42 (m, 15H, -C*H*); (**Isomer 2**) 1.14 (d, 3H, $^3J_{H\text{-}H}$= 6.3 Hz, -C*H*$_3$), 2.56 (m, 1H, -C*H*$_2$), 2.96 (ddd, 1H, $^2J_{P\text{-}H}$= 16.6 Hz, $^2J_{H\text{-}H}$= 13.9 Hz, $^3J_{H\text{-}H}$= 6.8 Hz, -C*H*$_2$), 5.04 (m, 1H, -C*H*), 7.28-7.42 (m, 15H, -C*H*). **^{1}H{^{31}P} NMR** (500 MHz, 298 K, $CDCl_3$): δ / ppm = (**Isomer 1**)) 1.52 (d, 3H, $^3J_{H\text{-}H}$= 6.1 Hz, -C*H*$_3$), 2.53 (dd, 1H, $^2J_{H\text{-}H}$= 14.5 Hz, $^3J_{H\text{-}H}$= 2.0 Hz, -C*H*$_2$), 2.75 (dd, 1H, $^2J_{H\text{-}H}$= 14.5 Hz, $^3J_{H\text{-}H}$= 7.8 Hz, -C*H*$_2$), 4.25 (dq, 1H, $^3J_{H\text{-}H}$= 7.9 Hz, $^3J_{H\text{-}H}$= 6.1 Hz -C*H*), 7.28-7.42 (m, 15H, -C*H*); (**Isomer 2**) 1.14 (d, 3H, $^3J_{H\text{-}H}$= 6.3 Hz, -C*H*$_3$), 2.56 (dd, 1H, $^2J_{H\text{-}H}$= 14.3 Hz, $^3J_{H\text{-}H}$= 5.2 Hz, -C*H*$_2$), 2.96 (dd, 1H, $^2J_{H\text{-}H}$= 13.9 Hz, $^3J_{H\text{-}H}$= 6.8 Hz, -C*H*$_2$), 5.04 (m, 1H, -C*H*), 7.28-7.42 (m, 15H, -C*H*). **^{13}C NMR** (126 MHz, 298 K, $CDCl_3$): δ / ppm = (**Isomer 1**) 24.7 (d, 1C, $^3J_{P\text{-}C}$= 4.1 Hz, -CH$_3$), 39.5 (d, 1C, $^1J_{P\text{-}C}$= 60.8 Hz, -CH$_2$), 65.6 (d, 1C, $^1J_{P\text{-}C}$= 73.3 Hz, -CPh$_3$), 72.1 (d, 1C, $^2J_{P\text{-}C}$= 20.2 Hz, -CH), 127.7 (d, 3C, $^5J_{P\text{-}C}$= 2.2 Hz, *para*-CH), 128.6 (d, 6C, $^4J_{P\text{-}C}$= 1.4 Hz, *meta*-CH), 130.6 (d, 6C, $^3J_{P\text{-}C}$= 6.6 Hz, *ortho*-CH), 140.5 (d, 3C, $^2J_{P\text{-}C}$= 3.0 Hz, *ipso*-*C*); (**Isomer 2**) 21.2 (d, 1C, $^3J_{C\text{-}H}$= 13.1 Hz, -CH$_3$), 38.9 (d, 1C, $^1J_{P\text{-}C}$= 64.0 Hz, -CH$_2$) , 66.2 (d, 1C, $^1J_{P\text{-}C}$= 71,9 Hz, -CPh$_3$), 74.3 (d, 1C, $^2J_{P\text{-}C}$= 20.2 Hz, -CH), 127.7 (d, 3C, $^5J_{P\text{-}C}$= 2.2 Hz, *para*-CH), 128.5 (d, 6C, $^4J_{P\text{-}C}$= 1.4 Hz, *meta*-CH), 131.1 (d, 6C, $^3J_{P\text{-}C}$= 6.3 Hz, *ortho*-CH), 140.3 (d, 3C, $^2J_{P\text{-}C}$= 3.0 Hz, *ipso*-*C*). **^{31}P{^{1}H} NMR** (202 MHz, 298 K, $CDCl_3$): δ /ppm = (**Isomer 1**) 62.1; (**Isomer 2**) 63.5. **MS** (ESI+) m/z (%) : 243.1 (100) $[CPh_3]^+$, 349.1 (25) $[M+H]^{+\cdot}$, 371.1 (8) $[M+Na]^+$. **HRMS** (APCI) : theor./exp. 349.1352/349.1356 $[M+H]^+$.

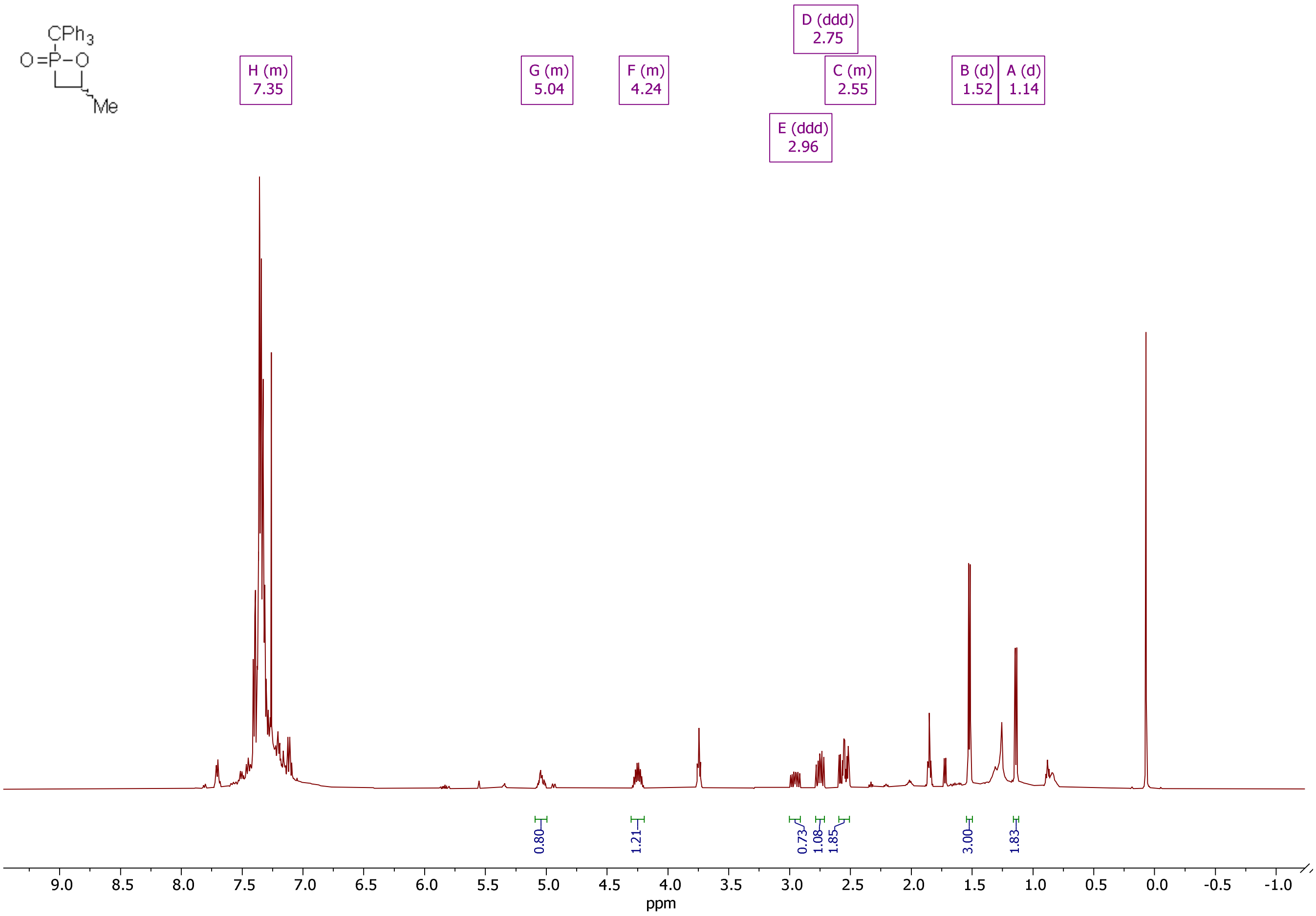

Figure S20: ^{1}H-NMR spectrum of **14a,a′** in $CDCl_3$.

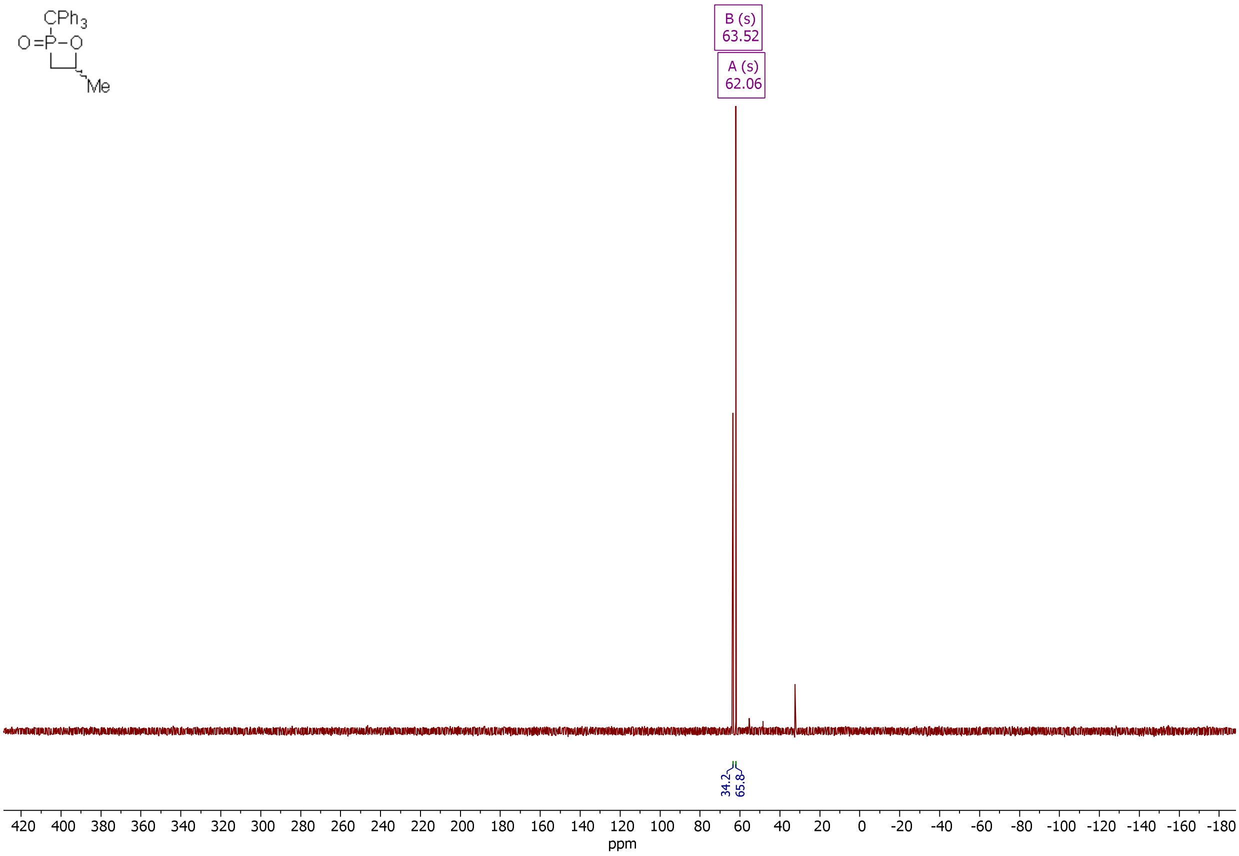

Figure S21: $^{31}P\{^1H\}$-NMR spectrum of **14a,a′** in $CDCl_3$.

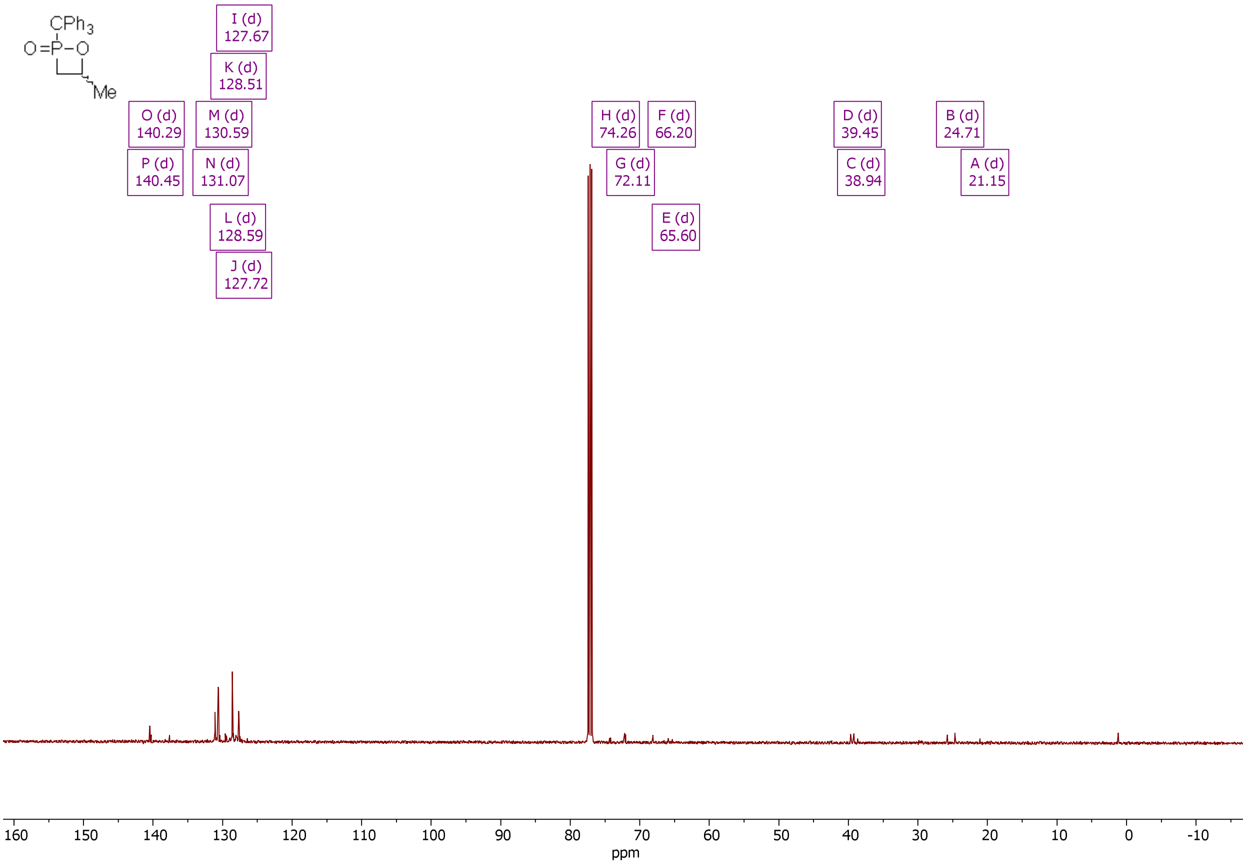

Figure S22: $^{13}C\{^{1}H\}$-NMR spectrum of **14a,a'** in $CDCl_3$.

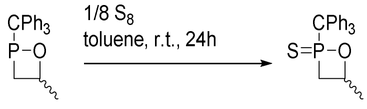

<u>Synthesis of **15a,a'**:</u> 0.2028 mg (0,61 mmol, 1eq) of complexes **4a,a'** was dissolved in 3 mL toluene. To that, 19.6 mg S_8 (0.61 mmol, 1 eq) were transferred with five times 3 mL toluene. The reaction was stirred for 24 h and the solvent was removed in vacuo (0.02 mbar). To help remove further traces of solvent, 10 mL of n-pentane were added. After removal of solvent in vacuo (0.02 mbar), the product was obtained as a brown solid.

Ratio (Isomer 1: Isomer 2) = 40:60. **^{1}H NMR** (500 MHz, 298 K, C_6D_6): δ / ppm = (**Isomer 1**) 0.75 (d, 3H, $^{3}J_{H\text{-}H}$= 6.2 Hz, -C*H*$_3$), 2.50 (m, 2H, -C*H*$_2$), 4.78 (m, 1H, -C*H*), 7.05 (m, 9H, *meta*/*para*-C*H*), 7.62 (m, 6H, *ortho*-C*H*); (**Isomer 2**) 1.21 (d, 3H, $^{3}J_{H\text{-}H}$= 6.1 Hz, -C*H*$_3$), 2.22 (ddd, 1H, $^{2}J_{P\text{-}H}$= 14.4 Hz, $^{2}J_{H\text{-}H}$= 14.4 Hz, $^{3}J_{H\text{-}H}$= 4.9 Hz, -C*H*$_2$), 2.72 (ddd, 1H, $^{2}J_{H\text{-}H}$= 13.6 Hz, $^{2}J_{P\text{-}H}$= 11.7 Hz, $^{3}J_{H\text{-}H}$= 8.3 Hz, -C*H*$_2$), 4.05 (m, 1H, -C*H*), 7.05 (m, 9H, *meta*/*para*-C*H*), 7.62 (m, 6H, *ortho*-C*H*). **^{13}C NMR** (126 MHz, 298 K, C_6D_6): δ / ppm = (**Isomer 1**) 21.4 (d, 1C, $^{3}J_{P\text{-}C}$= 13.5 Hz, -CH$_3$), 44.8 (d, 1C, $^{1}J_{P\text{-}C}$= 51.5 Hz, -CH$_2$), 70.4 (d, 1C, $^{1}J_{P\text{-}C}$= 46.0 Hz, -CPh$_3$), 75.2 (d, 1C, $^{2}J_{P\text{-}C}$= 19.4 Hz, -CH), 127.6 (d, 3C, $^{5}J_{P\text{-}C}$= 2.1 Hz, *para*-CH), 128.3 (d, 6C, $^{4}J_{P\text{-}C}$= 1.1 Hz, *meta*-CH), 131.9 (d, 6C, $^{3}J_{P\text{-}C}$= 6.9 Hz, *ortho*-CH), 141.6 (d, 3C, $^{2}J_{P\text{-}C}$= 1.4 Hz, *ipso*-C); (**Isomer 2**) 24.7 (d, 1C, $^{3}J_{C\text{-}H}$= 3.3 Hz, -CH$_3$), 44.4 (d, 1C, $^{1}J_{P\text{-}C}$= 49.8 Hz, -CH$_2$) , 69.8 (d, 1C, $^{1}J_{P\text{-}C}$= 47.8 Hz, -CPh$_3$), 74.5 (d, 1C, $^{2}J_{P\text{-}C}$= 19.9 Hz, -CH), 127.6 (d, 3C, $^{5}J_{P\text{-}C}$= 2.1 Hz, *para*-CH), 128.3 (d, 6C, $^{4}J_{P\text{-}C}$= 1.0 Hz, *meta*-CH), 131.4 (d, 6C, $^{3}J_{P\text{-}C}$= 6.7 Hz, *ortho*-CH), 141.9 (d, 3C, $^{2}J_{P\text{-}C}$= 1.4 Hz, *ipso*-C). **^{31}P{^{1}H} NMR** (202 MHz, 298 K, C_6D_6): δ /ppm = (**Isomer 1**) 115.8; (**Isomer 2**) 120.0. **MS** (ESI+) m/z (%) : 243.1 (100) [CPh$_3$]$^{+}$, 365.1 (5) [M+H]$^{+}$, 387.1 (7) [M+Na]$^{+}$. **HRMS** (ESI+): theor./exp. 365.1123/365.1115 [M+H]$^{+}$.

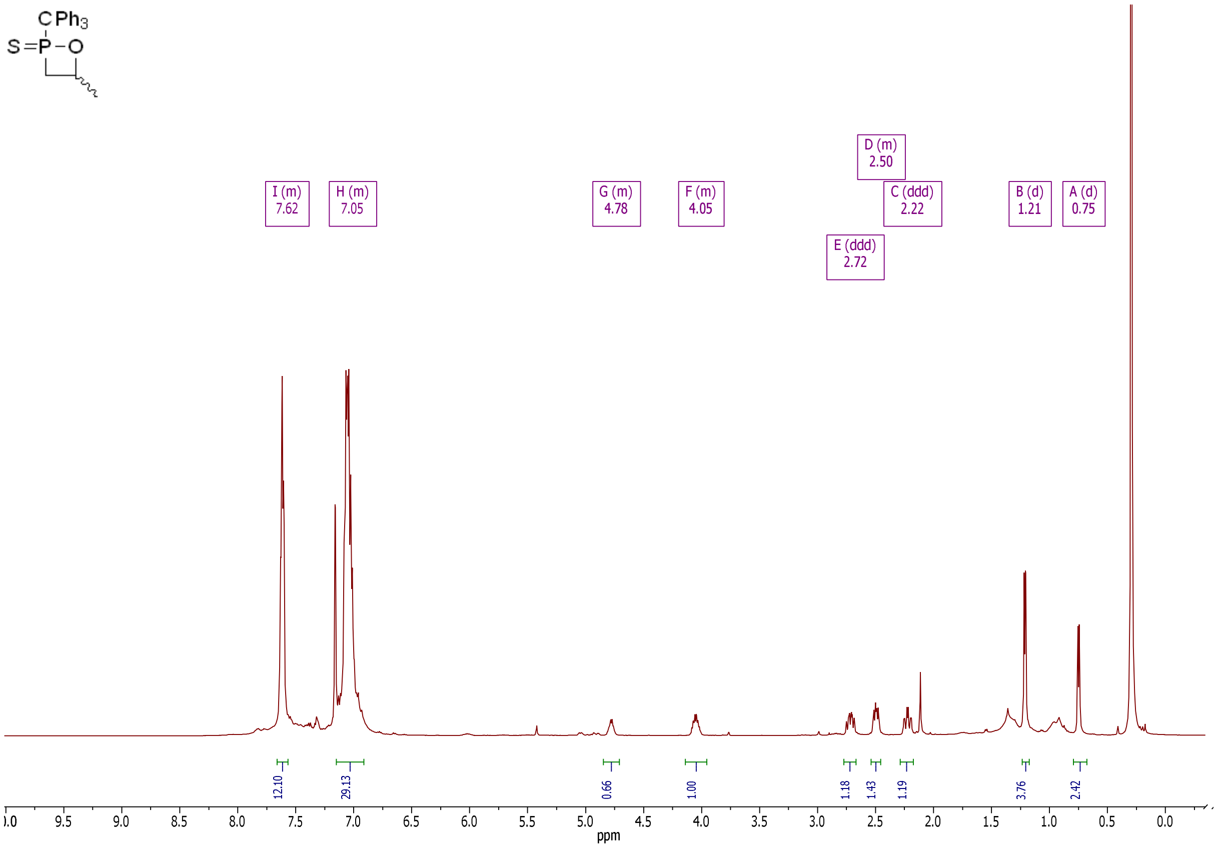

Figure S23: ^{1}H-NMR spectrum **15a,a′** in C_6D_6.

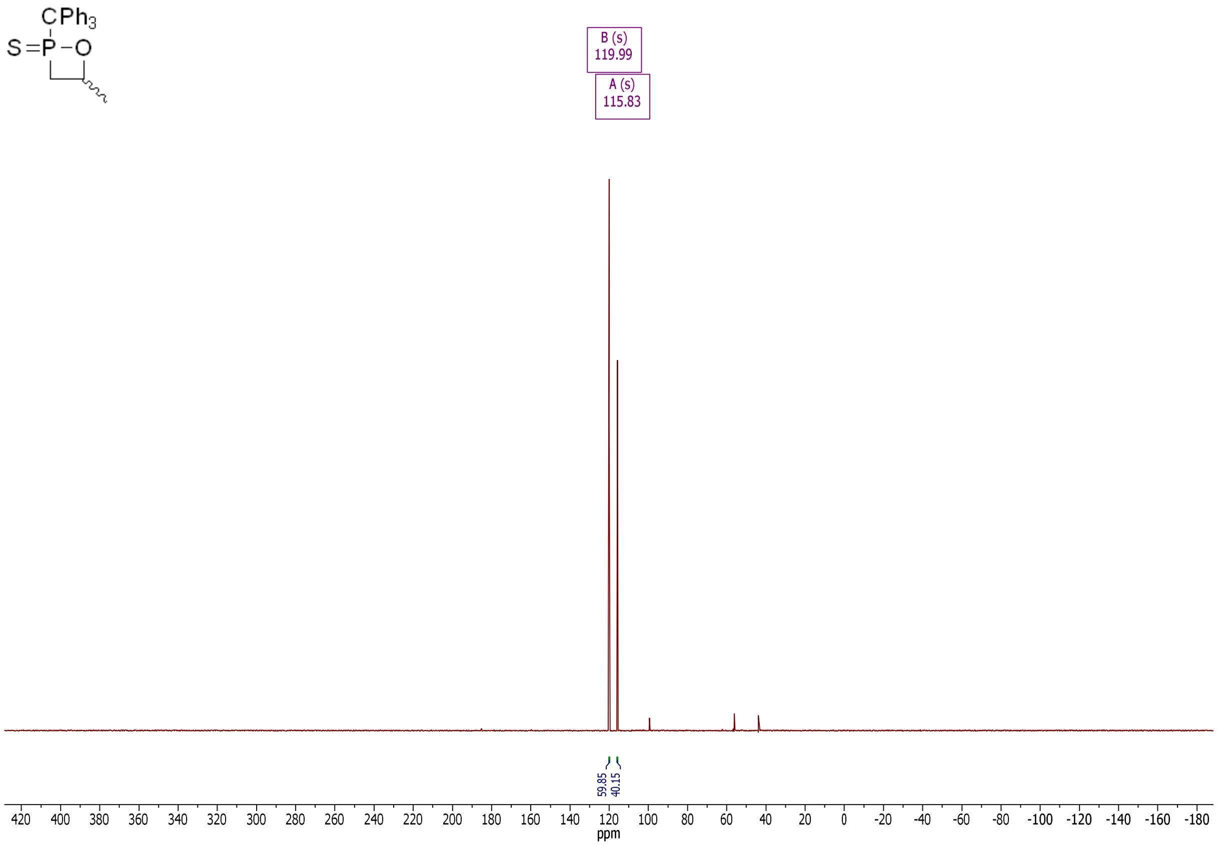

Figure S24: $^{31}P\{^{1}H\}$-NMR spectrum **15a,a′** in C_6D_6.

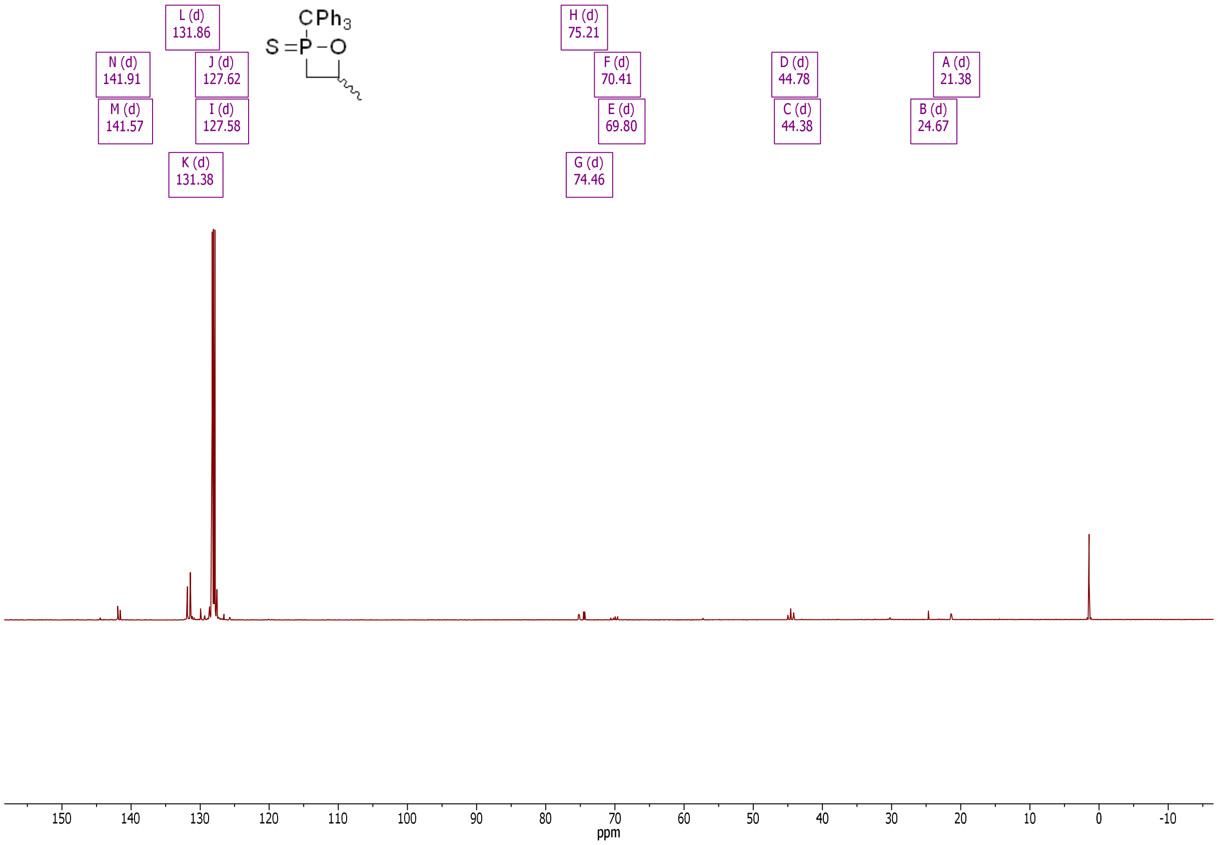

Figure S25: $^{13}C\{^1H\}$-NMR spectrum **15a,a'** in C_6D_6.

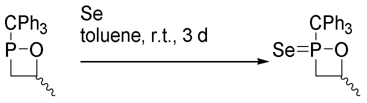

4a,a' **16a,a'**

<u>Synthesis of **16a,a'**:</u> 40.0 mg (0.5 mmol, 1 eq) selenium was placed in a Schlenk-tube. 167.0 mg (0.5 mmol, 1 eq) of 4a,a' was transferred into the selenium with five times 2 mL toluene. The reaction was stirred for 3 d. The reaction solution was filtrated to remove remaining selenium metal. After removal of solvent in vacuo (0.02 mbar), the product was obtained as a yellow solid.

Ratio (Isomer 1: Isomer 2) = 30:70. **^{1}H NMR** (500 MHz, 298 K, C_6D_6): δ / ppm = (**Isomer 1**) 0.75 (d, 3H, $^3J_{H\text{-}H}$= 6.3 Hz, -C*H*$_3$), 2.62 (ddd, 1H, $^2J_{P\text{-}H}$= 12.3 Hz, $^2J_{H\text{-}H}$= 12.3 Hz, $^3J_{H\text{-}H}$= 6.4 Hz, -C*H*$_2$), 2.74 (ddd, 1H, $^2J_{P\text{-}H}$= 13.0 Hz, $^2J_{H\text{-}H}$= 13.0 Hz, $^3J_{H\text{-}H}$= 8.2 Hz, -C*H*$_2$), 4.89 (m, 1H, -C*H*), 7.05 (m, 9H, *meta/para*-C*H*), 7.63 (m, 6H, *ortho*-C*H*); (**Isomer 2**) 1.27 (d, 3H, $^3J_{H\text{-}H}$= 6.2 Hz, -C*H*$_3$), 2.42 (ddd, 1H, $^2J_{P\text{-}H}$= 15.0 Hz, $^2J_{H\text{-}H}$= 13.7 Hz, $^3J_{H\text{-}H}$= 5.1 Hz, -C*H*$_2$), 2.98 (ddd, 1H, $^2J_{H\text{-}H}$= 13.8 Hz, $^2J_{P\text{-}H}$= 12.3 Hz, $^3J_{H\text{-}H}$= 8.3 Hz, -C*H*$_2$), 4.26 (m, 1H, -C*H*), 7.05 (m, 9H, *meta/para*-C*H*), 7.63 (m, 6H, *ortho*-C*H*). **^{13}C NMR** (126 MHz, 298 K, C_6D_6): δ / ppm = (**Isomer 1**) 21.7 (d, 1C, $^3J_{P\text{-}C}$= 12.7 Hz, -CH$_3$), 45.6 (d, 1C, $^1J_{P\text{-}C}$= 44.6 Hz, -CH$_2$), 70.4 (d, 1C, $^1J_{P\text{-}C}$= 33.4 Hz, -CPh$_3$), 76.1 (d, 1C, $^2J_{P\text{-}C}$= 19.4 Hz, -CH), 127.7 (d, 3C, $^5J_{P\text{-}C}$= 2.2 Hz, *para*-CH), 128.3 (d, 6C, $^4J_{P\text{-}C}$= 1.2 Hz, *meta*-CH), 131.8 (d, 6C, $^3J_{P\text{-}C}$= 7.0 Hz, *ortho*-CH), 141.3 (d, 3C, $^2J_{P\text{-}C}$= 0.5 Hz, *ipso*-C); (**Isomer 2**) 24.8 (d, 1C, $^3J_{C\text{-}H}$= 3.1 Hz, -CH$_3$), 44.6 (d, 1C, $^1J_{P\text{-}C}$= 43.5 Hz, -CH$_2$) , 69.8 (d, 1C, $^1J_{P\text{-}C}$= 35.4 Hz, -CPh$_3$), 76.1 (d, 1C, $^2J_{P\text{-}C}$= 19.4 Hz, -CH), 127.7 (d, 3C, $^5J_{P\text{-}C}$= 2.1 Hz, *para*-CH), 128.3 (d, 6C, $^4J_{P\text{-}C}$= 1.1 Hz, *meta*-CH), 131.3 (d, 6C, $^3J_{P\text{-}C}$= 6.9 Hz, *ortho*-CH), 146.7 (d, 3C, $^2J_{P\text{-}C}$= 0.5 Hz, *ipso*-C). **^{31}P{^{1}H} NMR** (202 MHz, 298 K, C_6D_6): δ /ppm = (**Isomer 1**) 115.8 (s_{sat}, $^1J_{Se\text{-}P}$= 839.7 Hz); (**Isomer 2**) 121.5 (s_{sat}, $^1J_{Se\text{-}P}$= 846.4 Hz). **^{77}Se{^{1}H} NMR** (95 MHz, 298 K, C_6D_6): δ /ppm = (**Isomer 1**) -10.7 (d_t, $^1J_{Se\text{-}P}$= 839.8 Hz); (**Isomer 2**) 79.4 (d, $^1J_{Se\text{-}P}$= 845.8 Hz). **MS** (LIFDI) m/z (%) : 243.1 (70) [CPh$_3$]$^+$, 412.1 (10) [M]$^{+\cdot}$. **HRMS** (ESI+): theor./exp. 413.0568/413.0558 [M+H]$^+$.

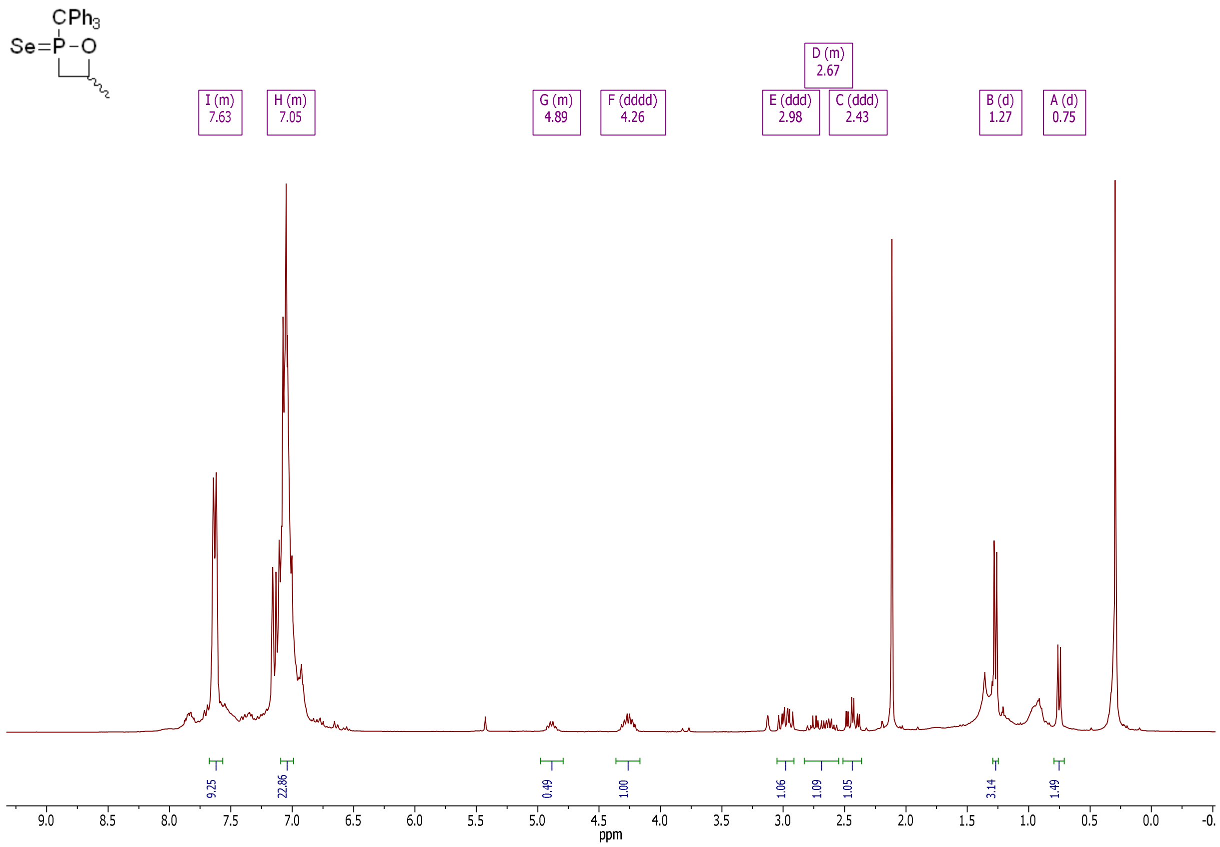

Figure S26: ^{1}H-NMR spectrum **16a,a′** in C_6D_6.

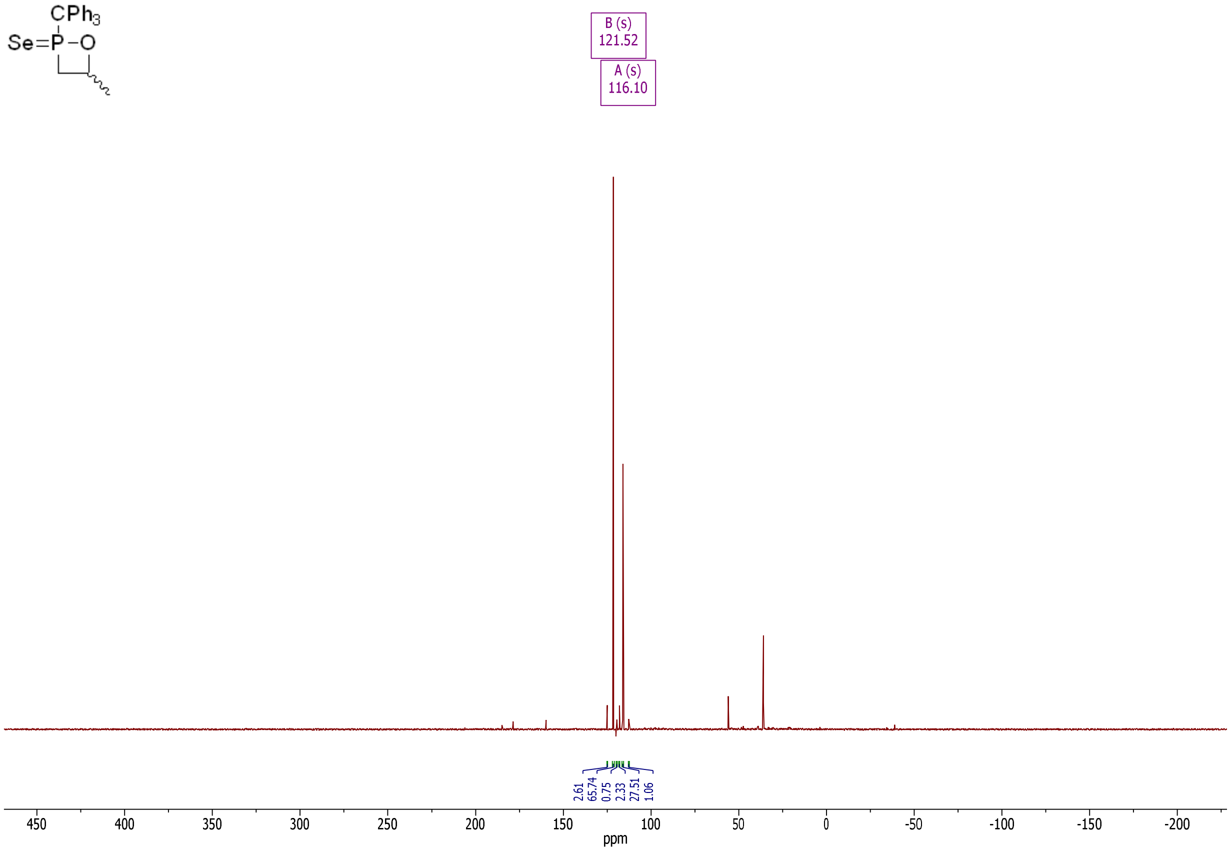

Figure S27: ^{31}P{^{1}H}-NMR spectrum **16a,a′** in C_6D_6.

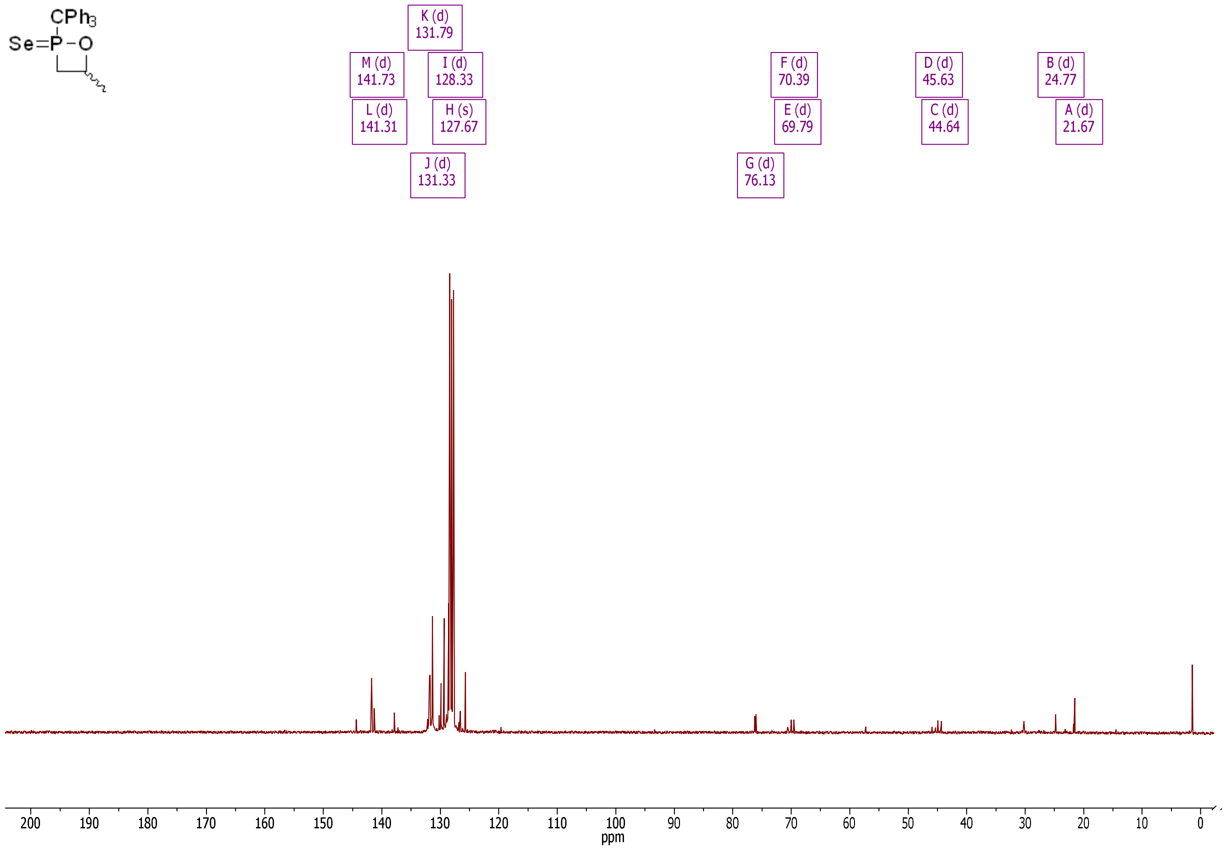

Figure S28: $^{13}C\{^{1}H\}$-NMR spectrum **16a,a′** in C_6D_6.

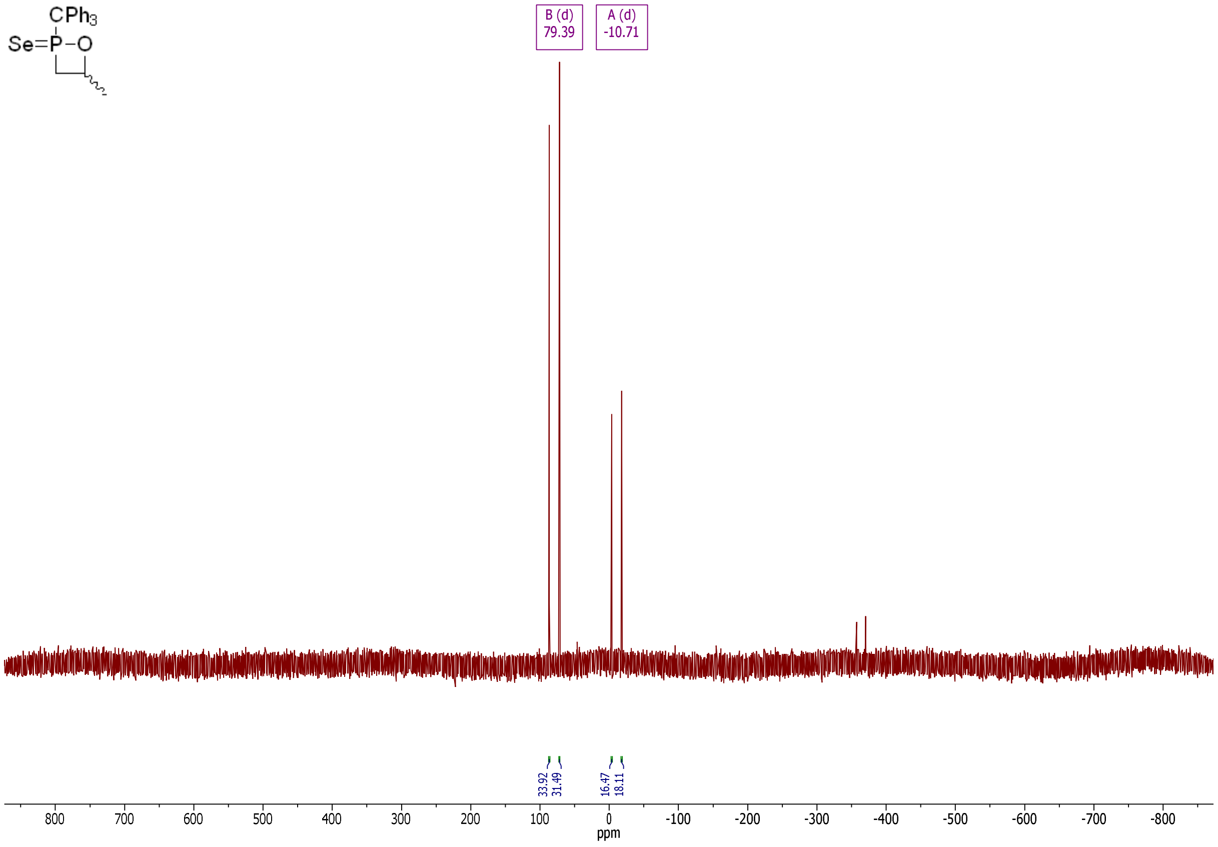

Figure S29: $^{77}Se\{^{1}H\}$-NMR spectrum **16a,a′** in C_6D_6.

X-ray crystallographic analyses of **3b,b′**, **3c,c′**, **3d,d′**, **4b,b′,** and **4c,c′**:

Suitable single crystals were obtained from concentrated diethyl ether (**3b,b′**, **3c,c′**, **3d,d′**) solutions upon slow evaporation of the solvent at 4°C, and *n*-pentane (**4b,b′**, **4c,c′**) solutions upon slow evaporation at ambient temperature. Data were collected on a Bruker X8-KappaApexII diffractometer equipped with a low-temperature device (Cryostream, Oxford Cryosystems) at 100 K using graphite monochromated Mo-Kα radiation (λ = 0.71073 Å). The structures were solved by Patterson methods (SHELXS-971) and refined by fullmatrix least squares on F2 (SHELXL-97,1 SHELXL-20152 or OLEX23). All iso-non-hydrogens were refined anisotropically. The hydrogen atoms were included isotropically using the riding model on the bound atoms. Adsorption corrections were carried out empirically (min./max. transmissions = 0.503179/0.746454 (**3b,b′**), 0.6562/0.7462 (**3c,c′**) 0.6696/0.4461 (**3d,d′**), 0.568294/0.746626 (**4b,b′**), 0.6756/0.7462 (**4c,c′**)). Crystallographic data for the structures reported in this paper have been deposited in the Cambridge Crystallographic Data Centre as supplementary publication no. CCDC 2157231 (**3b,b′**) 2157232 (**3c,c′**), CCDC 2157233 (**3d,d′**), CCDC 1995619 (**4b,b′**), CCDC 2157234 (**4c,c′**). The data can be obtained free of charge from the Cambridge Crystallographic Data Centre via www.ccdc.cam.ac.uk/structures

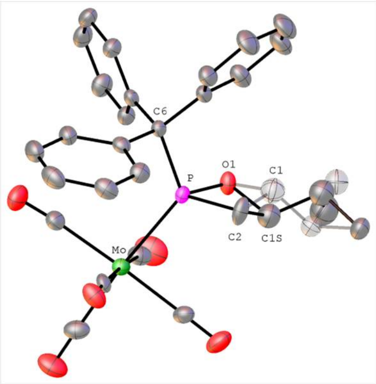

Crystal structure data of complex **3b,b′** ($C_{27}H_{21}MoO_6P$): crystal size 0.28 x 0.19 x 0.1 mm, triclinic, *P*-1̅ , a = 10.0029(5), b = 11.7627(6), c = 12.0605(6) Å, α = 79.599(3), β = 75.602(3), γ = 88.139(3)°, V = 1351.78(12) Å^3, Z = 2, ρ_{calc} = 1.465 g cm^{-3}, 2θ_{max} = 55.986°, collected (independent) reflections = 6495 (6495), R_{int} = 0.0863, μ = 0.585 mm^{-1}, 365 refined parameters, 110 restraints, R_1 (for I ≥ 2σ(I)) = 0.0483, wR_2 (for all data) = 0.1313, max./min. residual electron density = 3.18/-2.23 e · Å^{-3}.

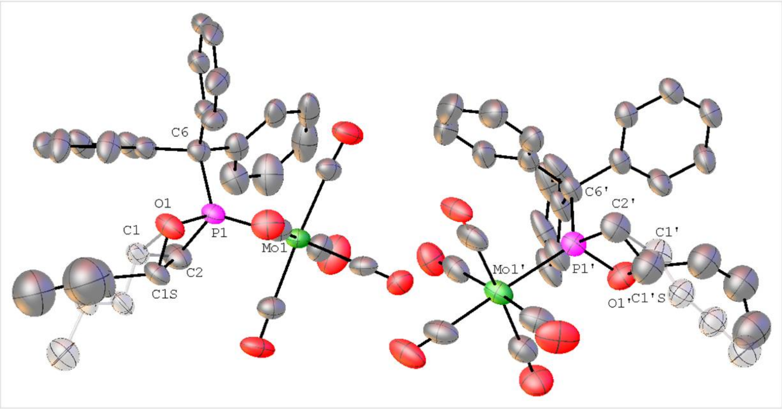

Crystal structure data of complex **3c,c′** ($C_{27}H_{21}MoO_6P$): crystal size 0.24 x 0.09 x 0.05 mm, monoclinic, *P*2$_1$/*n* , a = 18.6012(12), b = 16.2186(11), c = 18.6320(13) Å, α = 90, β = 105.119(3), γ = 90°, V = 5426.4(6) Å^3, Z = 8, ρ_{calc} = 1.460 g cm^{-3}, 2θ_{max} = 56°, collected (independent) reflections = 105226 (13077), R_{int} = 0.0667, μ = 0.583 mm^{-1}, 745 refined parameters, 141 restraints, R_1 (for I ≥ 2σ(I)) = 0.0470, wR_2 (for all data) = 0.1403, max./min. residual electron density = 1.50/-1.59 e · Å^{-3}.

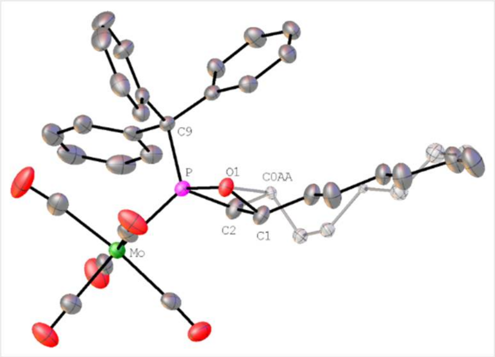

Crystal structure data of complex **3d,d′** ($C_{32}H_{31}MoO_6P$): crystal size 0.22 x 0.18 x 0.16 mm, monoclinic, *P*2$_1$/*n* , a = 11.7880(8), b = 14.8498(10), c = 17.6626(12) Å, α = 90, β = 102.469(3), γ = 90°, V = 3018.9(4) Å^3, Z = 4, ρ_{calc} = 1.405 g cm^{-3}, $2\theta_{max}$ = 55.996°, collected (independent) reflections = 57895 (7274), R_{int} = 0.0498, μ = 0.529 mm^{-1}, 403 refined parameters, 42 restraints, R_1 (for I ≥ 2σ(I)) = 0.0344, wR_2 (for all data) = 0.0698, max./min. residual electron density = 0.43/-0.41 e · Å^{-3}.

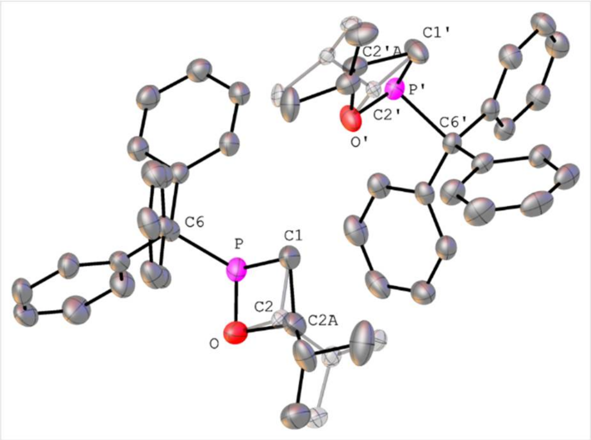

Crystal structure data of complex **4b,b′** ($C_{24}H_{25}OP$): crystal size 0.32 x 0.21 x 0.15 mm, triclinic, *P*-1 , a = 8.7583(9), b = 11.4693(12), c = 20.633(2) Å, α = 74.925(3), β = 78.069(3), γ = 89.940(3)°, V = 1955.0(4) Å^3, Z = 4, ρ_{calc} = 1.225 g cm^{-3}, $2\theta_{max}$ = 54°, collected (independent) reflections = 8458 (8458), R_{int} = 0.0565, μ = 0.150 mm^{-1}, 551 refined parameters, 6 restraints, R_1 (for I ≥ 2σ(I)) = 0.0525, wR_2 (for all data) = 0.1551, max./min. residual electron density = 0.66/-0.48 e · Å^{-3}.

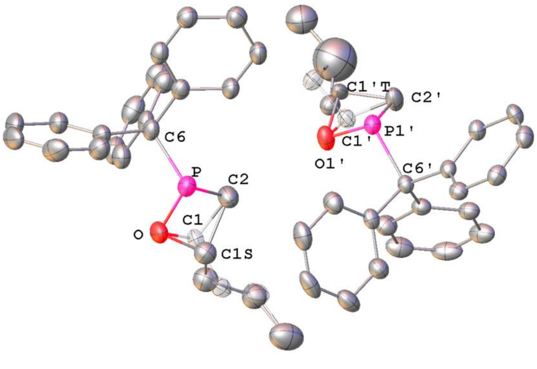

Crystal structure data of complex **4c,c′** ($C_{24}H_{25}OP$): crystal size 0.12 x 0.09 x 0.06 mm, triclinic, *P*-1 , a = 8.7329(5), b = 10.7973(6), c = 21.5282(13) Å, α = 78.245(3), β = 80.427(3), γ = 89.336(2)°, V = 1959.2(2) Å^3, Z = 4, ρ_{calc} = 1.222 g cm^{-3}, $2\theta_{max}$ = 56°, collected (independent)

reflections = 58756 (9467), R_{int} = 0.1209, μ = 0.150 mm^{-1}, 536 refined parameters, 60 restraints, R_1 (for I ≥ 2σ(I)) = 0.0631, wR_2 (for all data) = 0.1985, max./min. residual electron density = 0.59/-0.41 e · $Å^{-3}$.

COMPUTATIONAL PART

Table S1: Phosphorus pyramidalization and P-O bond properties of complexes **9** and **9^{OMe2}**.

	**9**	**9^{OMe2}**
d[a)]	1.903	1.902
∑<P[b)]	307.3	313.0
WBI	0.415	0.411
MBO	0.573	0.569
LBO	0.859	0.832
ρ(r)[c)]	0.0922	0.0942
$-¼\nabla^2(\rho(r))$[c)]	-0.0177	-0.0177
G(r)/ ρ(r)[c)]	0.8490	0.8570

[a)] In Å. [b)] In degrees. [c)] In atomic units.

Table S2: C-O bond properties of **2e**, **2e-Li(DEGDME)** and **9**.

	**2e**		**2e-Li(DEGDME)**		**9**	
	Non-benzylic C-O	Benzylic C-O	Non-benzylic C-O	Benzylic C-O	Non-benzylic C-O	Benzylic C-O
d[a)]	1.416	1.413	1.427	1.437	1.448	1.459
WBI	0.945	0.941	0.908	0.881	0.845	0.822
MBO	0.980	1.000	0.945	0.778	0.868	0.746
LBO	1.415	1.405	1.326	1.278	1.233	1.189
ρ(r)[b)]	0.2600	0.2626	0.2478	0.2435	0.235	0.230
q^{N}_{C} [c)]	-0.118	0.0264	-0.104	0.035	-0.102	0.059

[a)] In Å. [b)] In atomic units. [c)] In e.

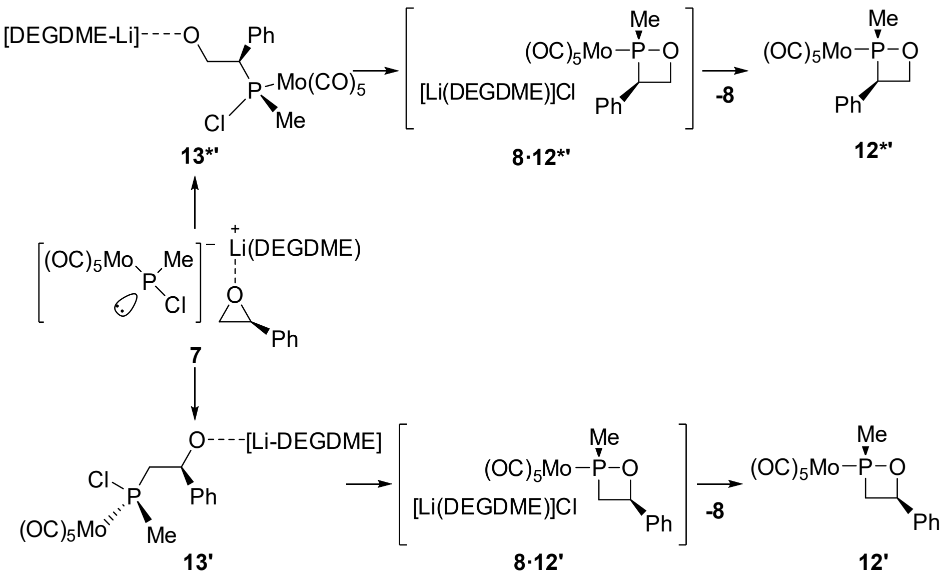

Scheme S1: Mechanistic proposal for the formation of 1,2-oxaphosphetanes **12′** and **12b*′** from the direct nucleophilic attack of the P atom to the epoxide C atoms of **7**.

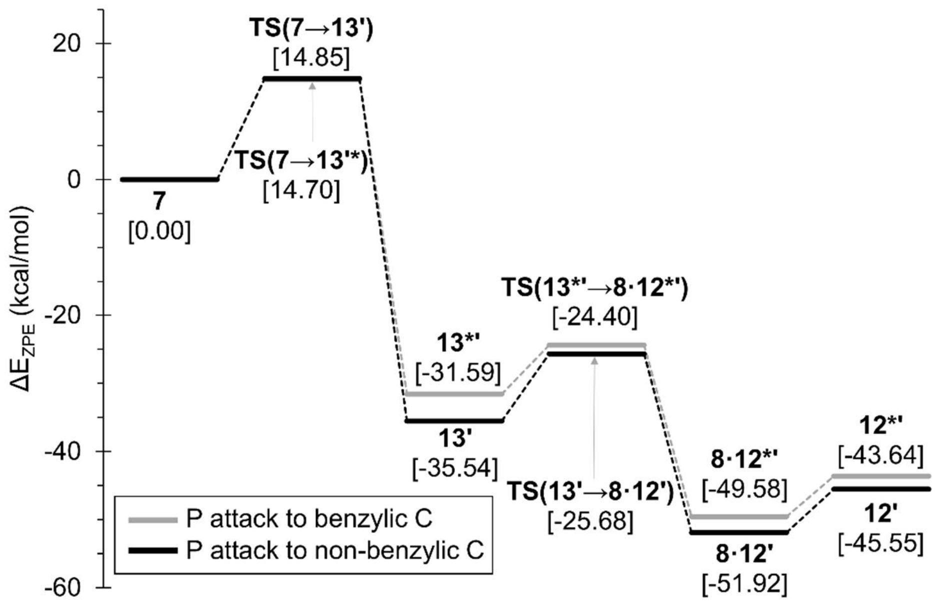

Figure S30: Calculated (CPCM$_{THF}$/CCSD(T)/def2-TZVPP(ecp)) minimum energy profile for the conversion of **7** into **12′** and **12*′**.

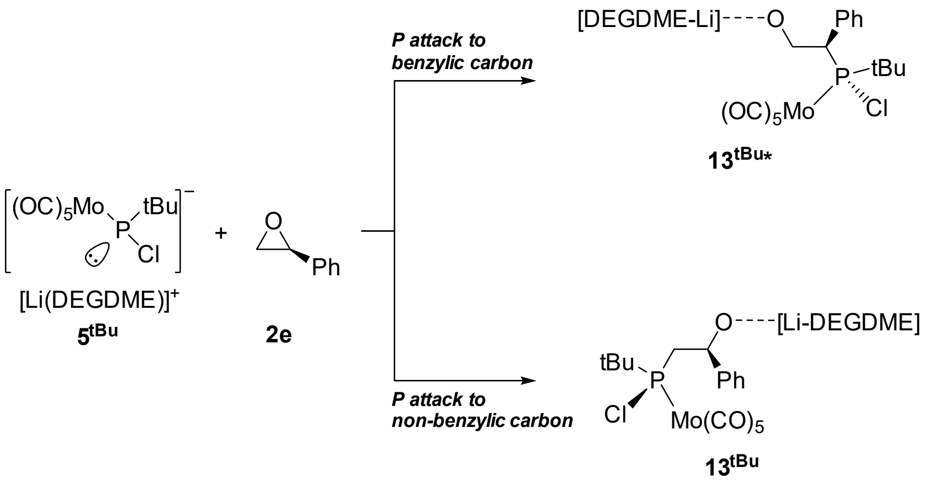

Scheme S2: Nucleophilic attack of the P atom of **5^{tBu}** to C atoms of styrene oxide **2e** giving rise to **13^{tBu}** and **13^{tBu*}**.

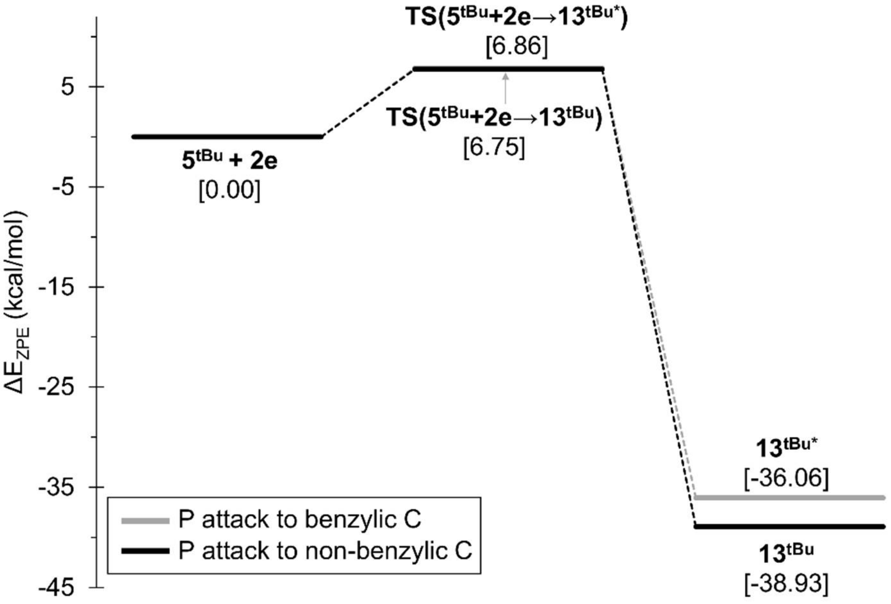

Figure S2: Calculated (CPCM$_{THF}$/CCSD(T)/def2-TZVPP(ecp)) minimum energy profile for the conversion of **5^{tBu}+2e** into **13^{tBu}** and **13^{tBu*}**.

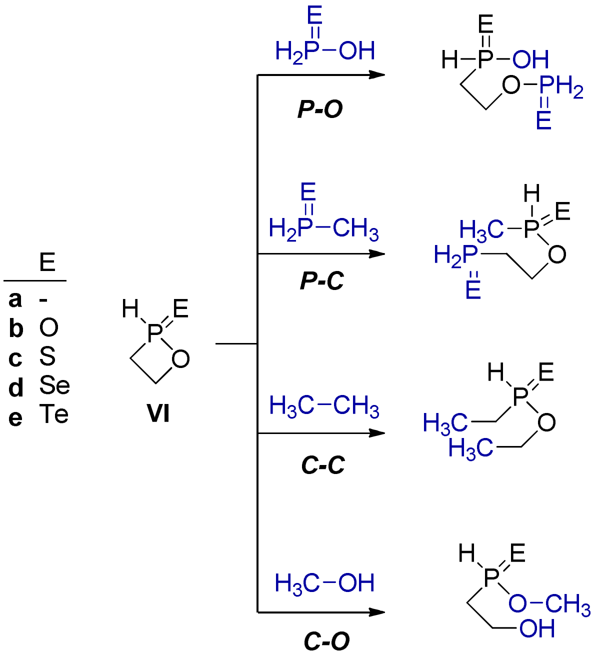

Scheme S3: Homodesmotic reactions for RSE evaluation on derivatives **VIa-e**.

Calculated structures

Cartesian coordinates (in Å) and ZPE (in hartrees) for minima and transition states were computed at PBEh-3c(ecp) level. Electronic energies (in hartrees) are quoted at the (gas-phase) DLPNO-CCSD(T)/def2-TZVPP. Imaginary frequencies are obtained upon frequency calculation (at the optimization level).

2e:

E = -384.175742996004 au
ZPE = 0.14302222 au

O	-1.447779	-1.401226	4.677390
C	-0.543410	-1.699030	3.634030
C	-1.979551	-1.947846	3.484489
H	-0.129174	-0.829318	3.125506
H	-2.582784	-1.275913	2.879993
H	-2.347968	-2.968174	3.552334
C	0.416857	-2.810992	3.851267
C	1.498657	-2.952091	2.986786
C	2.396629	-3.998210	3.144696
C	2.226705	-4.910610	4.177200
C	1.155189	-4.767778	5.049368
C	0.255076	-3.723910	4.890049
H	1.641376	-2.238669	2.183400
H	3.232059	-4.097059	2.463843
H	2.928121	-5.724652	4.305289
H	1.021403	-5.470208	5.861907
H	-0.569600	-3.617711	5.583308

5:

E = -1944.12425575657 au
ZPE = 0.29761021 au

Li	-2.786763	-1.920830	4.613552
O	-3.928631	-0.443625	3.899261
C	-3.057342	0.624271	3.572127
H	-2.633073	0.383682	2.596866
H	-3.596215	1.571444	3.473942
C	-1.953454	0.758061	4.601972
H	-2.319122	1.198900	5.538483
H	-1.176349	1.423116	4.208858
O	-1.439722	-0.537870	4.843322
C	-0.289837	-0.555220	5.664520
H	-0.495618	-0.124720	6.649040
H	0.013536	-1.592635	5.788068
H	0.530321	-0.000404	5.202237
C	-4.930397	-0.158673	4.864447
H	-4.576647	0.571573	5.600920
H	-5.819567	0.263613	4.385876
C	-5.277578	-1.471341	5.531946
H	-6.030826	-1.313597	6.310141
H	-5.689126	-2.168007	4.799489
O	-4.110778	-2.086146	6.063334
C	-3.747042	-1.635780	7.357201
H	-4.544843	-1.838318	8.076096
H	-2.857159	-2.185180	7.657556
H	-3.520411	-0.565783	7.380913
P	-2.423266	-3.664759	2.894850
Cl	-4.531395	-4.073054	2.668109
C	-1.910314	-5.374463	3.366879
H	-0.838571	-5.349167	3.574807
H	-2.420127	-5.702102	4.272711
H	-2.085281	-6.103070	2.575976
Mo	-1.551170	-3.066121	0.578335
C	-2.646567	-4.639962	-0.113711
O	-3.255739	-5.534481	-0.477289
C	-0.484536	-1.558846	1.416831
O	0.128554	-0.714501	1.888389
C	0.029531	-4.307729	0.851512
O	0.922004	-5.006556	1.007476
C	-0.910978	-2.578144	-1.237022
O	-0.545086	-2.305004	-2.293780
C	-3.175419	-1.848936	0.404130
O	-4.084758	-1.163396	0.308861

6:

E = -2328.30867786565 au
ZPE = 0.44177057 au

Li	-3.338853	0.775371	3.791617
O	-3.917104	1.761624	5.495690
C	-2.723888	2.165683	6.145512
H	-2.388200	3.068760	5.633206
H	-2.901521	2.423839	7.194084
C	-1.664722	1.086896	6.048803
H	-1.896196	0.231660	6.697139
H	-0.701872	1.493256	6.379243
O	-1.608925	0.671706	4.698502
C	-0.668131	-0.357813	4.454442
H	-0.882708	-1.246325	5.056072
H	-0.747962	-0.621880	3.402774

H	0.349035	-0.020913	4.671162
C	-4.756831	0.887491	6.236518
H	-4.167866	0.194552	6.847953
H	-5.403804	1.453976	6.914017
C	-5.593498	0.129432	5.229030
H	-6.232265	-0.598231	5.739478
H	-6.242205	0.816653	4.682994
O	-4.759746	-0.493933	4.267715
C	-4.281493	-1.777503	4.625342
H	-5.104570	-2.491962	4.706612
H	-3.607317	-2.096284	3.833244
H	-3.728769	-1.771007	5.570221
C	-0.414791	-3.158947	1.688849
C	-1.092293	-4.000732	0.698774
H	0.391666	-2.518708	1.332917
H	-0.774776	-3.969801	-0.339559
H	-1.542275	-4.937637	1.017018
C	-0.317241	-3.539838	3.119697
C	0.891559	-3.378812	3.788995
C	1.005537	-3.711299	5.132159
C	-0.094001	-4.202073	5.822008
C	-1.304493	-4.364010	5.158442
C	-1.416832	-4.035701	3.816076
H	1.750047	-2.981937	3.259590
H	1.951930	-3.579337	5.640324
H	-0.010032	-4.456454	6.870528
H	-2.166540	-4.745825	5.690369
H	-2.368463	-4.157097	3.313679
P	-3.042597	-0.069147	1.359266
O	-1.697562	-2.824245	1.198846
Mo	-4.836243	-1.025918	-0.173220
C	-6.245116	-1.762928	-1.358964
O	-7.060483	-2.180389	-2.057761
C	-3.368327	-1.783175	-1.352201
O	-2.543480	-2.207176	-2.022561
C	-4.756384	0.728330	-1.198345
O	-4.689622	1.719146	-1.764273
C	-4.807261	-2.726758	0.938660
O	-4.813964	-3.689468	1.557245
C	-6.219800	-0.235049	1.094624
O	-7.002997	0.205179	1.801426
C	-1.571330	0.462267	0.373144
H	-0.885945	1.057974	0.976376
H	-1.050060	-0.446066	0.067618
H	-1.831837	1.019663	-0.525791
Cl	-3.739238	2.019645	1.793606

TS(**6**→**7**):

E = -2328.30701717938 au
ZPE = 0.44191363 au
ν = -36.83 cm^{-1}

Li	-2.784568	-0.132564	3.589107
O	-4.042047	1.122138	4.528786
C	-3.230318	2.191113	4.980728
H	-3.054746	2.822824	4.109499
H	-3.739490	2.794715	5.738513
C	-1.915306	1.680597	5.533065
H	-2.049779	1.166145	6.493821
H	-1.245622	2.530035	5.709759
O	-1.362017	0.790098	4.579637
C	-0.095749	0.282255	4.947925
H	-0.146505	-0.286791	5.881050
H	0.243254	-0.377011	4.151268
H	0.632887	1.088234	5.066929
C	-4.769832	0.417040	5.521015
H	-4.267577	0.462567	6.493461
H	-5.767595	0.848369	5.648800
C	-4.881790	-1.013985	5.040526
H	-5.398688	-1.628144	5.785005
H	-5.463673	-1.056251	4.117519
O	-3.598335	-1.539586	4.739814
C	-2.899981	-2.032089	5.870014
H	-3.440838	-2.865725	6.324600
H	-1.931833	-2.393012	5.531345
H	-2.742433	-1.262978	6.632463
C	-0.632792	-2.761333	2.172929
C	-1.065857	-3.207837	0.847342
H	0.143054	-1.996593	2.210242
H	-0.606358	-2.777472	-0.038220
H	-1.452336	-4.216002	0.723616
C	-0.698578	-3.642599	3.364440
C	0.361824	-3.654420	4.265043
C	0.327832	-4.473660	5.386097
C	-0.771531	-5.288017	5.616662
C	-1.836069	-5.275370	4.723029
C	-1.805060	-4.454367	3.607120
H	1.222670	-3.019111	4.090559
H	1.159384	-4.472276	6.078734
H	-0.802176	-5.926513	6.489892
H	-2.700686	-5.900978	4.903220
H	-2.653410	-4.432864	2.935505
P	-3.089687	0.409780	1.187151
O	-1.863260	-2.332033	1.621543
Mo	-5.035160	-0.655179	-0.083144
C	-6.573139	-1.431344	-1.062322
O	-7.465711	-1.872125	-1.643297
C	-3.702093	-1.643598	-1.245608
O	-2.944658	-2.204855	-1.895700
C	-4.938694	0.944607	-1.338987
O	-4.864617	1.838037	-2.047244
C	-5.025414	-2.225182	1.204875
O	-5.053064	-3.124743	1.911994
C	-6.247663	0.374282	1.187167
O	-6.920745	0.941393	1.917185
C	-1.625374	0.621955	0.078199
H	-0.861814	1.236030	0.556048
H	-1.198086	-0.362842	-0.109779
H	-1.888124	1.066742	-0.880958
Cl	-3.582680	2.519211	1.290950

7: E = -2328.31348210103 au
ZPE = 0.44179168 au

Li	-2.531153	-0.216078	3.483560
O	-3.935140	1.169287	4.272346
C	-3.181847	2.298999	4.661093
H	-2.950339	2.841076	3.743881
H	-3.757064	2.968943	5.308962
C	-1.906771	1.883952	5.361240
H	-2.110297	1.450414	6.349859
H	-1.277143	2.767674	5.518999
O	-1.249936	0.936001	4.541012
C	-0.009720	0.515603	5.071013
H	-0.136544	-0.001430	6.027502
H	0.447168	-0.166079	4.358128
H	0.662618	1.364991	5.220917
C	-4.737593	0.582692	5.277554
H	-4.292279	0.704786	6.271771
H	-5.729256	1.047003	5.307783
C	-4.864145	-0.882716	4.926123
H	-5.439197	-1.411289	5.694165
H	-5.396594	-0.995772	3.980042
O	-3.585682	-1.463420	4.745581
C	-2.953879	-1.851291	5.950149
H	-3.546120	-2.605550	6.476082
H	-1.990959	-2.287301	5.694275
H	-2.786248	-1.008825	6.628536
C	-0.254873	-2.660680	2.836572
C	-0.984416	-2.387278	1.596505
H	0.751368	-2.253833	2.916656
H	-0.500194	-1.818543	0.809368
H	-1.753233	-3.079482	1.265886
C	-0.501523	-3.867678	3.662532
C	0.582787	-4.508190	4.253214
C	0.396771	-5.654985	5.013517
C	-0.879981	-6.165537	5.196646
C	-1.967751	-5.524169	4.615921
C	-1.782052	-4.382712	3.852305
H	1.581658	-4.109729	4.119219
H	1.249284	-6.144707	5.465481
H	-1.029120	-7.056993	5.791631
H	-2.966824	-5.914328	4.760089
H	-2.641679	-3.887723	3.419566
P	-3.203362	0.468061	1.047381
O	-1.274655	-1.669962	2.783574
Mo	-5.116389	-0.818955	-0.069132
C	-6.633688	-1.746762	-0.947104
O	-7.514872	-2.273773	-1.468973
C	-3.769175	-1.747488	-1.263016
O	-2.998246	-2.277506	-1.923612
C	-5.205625	0.742209	-1.381448
O	-5.236260	1.614014	-2.117708
C	-4.934118	-2.331441	1.259560
O	-4.847936	-3.208371	1.993454
C	-6.347826	0.166079	1.221450
O	-7.031278	0.709762	1.958709
C	-1.875969	0.787669	-0.205005
H	-1.122113	1.467594	0.192814
H	-1.383133	-0.158347	-0.435180
H	-2.261318	1.195990	-1.138760
Cl	-3.893231	2.507159	1.146136

8: E = -929.358852882432 au
ZPE = 0.21375606 au

Li	-2.777667	-1.687658	4.595615
O	-4.278749	-0.485899	3.937650
C	-4.161780	0.718219	4.675844
H	-3.345913	1.278126	4.216392
H	-5.067761	1.327747	4.602811
C	-3.843837	0.437471	6.130524
H	-4.710685	0.026220	6.664706
H	-3.573923	1.377211	6.626537
O	-2.771276	-0.481825	6.158337
C	-2.317553	-0.796627	7.458866
H	-3.113720	-1.240693	8.064341
H	-1.505340	-1.513624	7.360391
H	-1.943102	0.093013	7.972258
C	-5.509901	-1.179799	4.074321
H	-5.960900	-1.000925	5.056484
H	-6.228449	-0.843357	3.320137
C	-5.212608	-2.651134	3.882696
H	-6.127897	-3.239606	4.004257
H	-4.834370	-2.829635	2.874097
O	-4.199518	-3.081458	4.777453
C	-4.662158	-3.428724	6.069273
H	-5.384537	-4.247453	6.016889
H	-3.801495	-3.758310	6.647698
H	-5.129847	-2.587744	6.590384
Cl	-0.917226	-2.148299	3.571062

9: E = -1398.92232106566 au
ZPE = 0.22735479 au

C	0.452157	-1.944143	3.016723
C	0.496768	-1.820524	1.558874

H	1.091234	-1.268116	3.577781
H	1.173475	-1.099571	1.116212
H	0.203580	-2.651029	0.927319
C	0.043299	-3.173590	3.727249
C	0.710671	-3.511549	4.899527
C	0.382619	-4.675363	5.580491
C	-0.624734	-5.498450	5.099331
C	-1.302138	-5.156429	3.934850
C	-0.969500	-4.000997	3.246838
H	1.490928	-2.865403	5.282877
H	0.909674	-4.932829	6.489346
H	-0.886792	-6.403223	5.631541
H	-2.094005	-5.791873	3.561627
H	-1.514895	-3.747870	2.346195
P	-1.130855	0.564854	2.507945
O	-0.612059	-1.253771	2.296946
Mo	-2.579297	0.893765	0.462715
C	-3.778276	1.184250	-1.107438
O	-4.459806	1.349394	-2.016497
C	-1.050568	0.309700	-0.735537
O	-0.183235	-0.019018	-1.405364
C	-1.913825	2.812703	0.356388
O	-1.532940	3.888270	0.293712
C	-3.184770	-1.043772	0.622569
O	-3.517431	-2.133411	0.709651
C	-4.056876	1.462203	1.747209
O	-4.883907	1.775688	2.469038
C	0.528572	1.313774	2.202175
H	1.278329	1.002280	2.931477
H	0.926288	1.196964	1.194244
H	0.375104	2.384903	2.362475

TS(**9**→**10**+**11**): E = -1398.88955074816 au

ZPE = 0.22391314 au

ν = -403.82 cm^{-1}

C	-0.062947	-2.258891	2.383416
C	-0.383191	-2.704977	1.059618
H	0.774821	-1.565013	2.429808
H	-0.089075	-2.121585	0.199397
H	-0.986228	-3.587346	0.897508
C	-0.037882	-3.234365	3.508589
C	1.072586	-3.281826	4.343822
C	1.128777	-4.197153	5.386203
C	0.067743	-5.063405	5.604539
C	-1.048711	-5.013124	4.777631
C	-1.103023	-4.104388	3.733187
H	1.900968	-2.602958	4.179572
H	1.998709	-4.228322	6.028626
H	0.106981	-5.775325	6.418443
H	-1.880214	-5.683818	4.948974
H	-1.982103	-4.073887	3.101162
P	-1.574467	0.157078	2.428054
O	-1.368493	-1.505973	2.393973
Mo	-2.412480	1.133641	0.275921
C	-3.009727	1.859363	-1.485486
O	-3.331425	2.266474	-2.507978
C	-0.555641	0.800872	-0.473620
O	0.495309	0.616573	-0.882919
C	-1.818710	2.982844	0.886140
O	-1.483488	4.018923	1.228864
C	-2.991562	-0.744944	-0.253777
O	-3.305005	-1.803737	-0.541272
C	-4.260581	1.466571	1.090902
O	-5.293695	1.656190	1.534149
C	0.057395	0.805333	2.960421
H	0.403215	0.355101	3.891239
H	0.837133	0.706879	2.201322
H	-0.078202	1.873011	3.142054

10: E = -309.065088722863 au

ZPE = 0.1371165 au

C	0.340866	-2.982784	4.388844
C	-0.757329	-2.240034	4.262000
H	1.281076	-2.475525	4.582952
H	-0.707778	-1.163763	4.351137
H	-1.736462	-2.658970	4.067421
C	0.436408	-4.444115	4.295000
C	1.685406	-5.047903	4.451071
C	1.831704	-6.425631	4.371304
C	0.726004	-7.228354	4.133067
C	-0.525031	-6.641020	3.975725
C	-0.668761	-5.266387	4.055593
H	2.554804	-4.428326	4.637547
H	2.810457	-6.870427	4.495770
H	0.834669	-8.303146	4.069896
H	-1.393413	-7.259744	3.789783
H	-1.653172	-4.833671	3.929768

11: E = -1089.93862942533 au

ZPE = 0.08555665 au

O	-1.259907	-2.196738	6.329296
P	-0.197407	-2.643336	7.271612
Mo	1.457939	-1.361287	8.389359
C	-0.325201	-4.436550	7.523721
H	-0.657342	-4.933201	6.612787
H	0.623455	-4.854160	7.856677
H	-1.069450	-4.619373	8.303957
C	1.332556	-2.607523	10.001174
O	1.263977	-3.302780	10.899943
C	0.004202	-0.148783	9.180108
O	-0.798528	0.527713	9.615558
C	2.886719	-0.262493	9.348182
O	3.682454	0.351168	9.880639
C	1.554808	-0.114373	6.765119
O	1.602857	0.578480	5.865496
C	2.924555	-2.548102	7.599224
O	3.742677	-3.208641	7.164609

TS(**9**→**12***):

E = -1398.90543164361 au

ZPE = 0.22608919 au

ν = -206.84 cm^{-1}

C	0.642336	-1.813787	3.527319
C	1.594506	-1.588812	2.402347
H	0.489306	-0.969467	4.193611
H	2.382765	-0.891708	2.701557
H	2.056981	-2.507259	2.039660
C	-0.170397	-2.920711	3.710778
C	-1.165422	-2.858994	4.721876
C	-1.998659	-3.928522	4.938537
C	-1.847911	-5.076641	4.160814
C	-0.873069	-5.164816	3.163707
C	-0.043080	-4.098668	2.929182
H	-1.261280	-1.958770	5.315164
H	-2.761960	-3.885675	5.701858
H	-2.503141	-5.920463	4.332772
H	-0.783579	-6.067150	2.576296
H	0.702270	-4.157334	2.148760
P	0.152425	0.551937	1.723905
O	0.733579	-1.075634	1.420349
Mo	-2.368593	0.501085	1.231723
C	-4.329436	0.508838	0.918797
O	-5.465649	0.512969	0.728206
C	-1.940561	0.400892	-0.751356
O	-1.684879	0.329699	-1.864001
C	-2.283655	2.531286	1.151097
O	-2.226350	3.673024	1.103397
C	-2.339918	-1.525186	1.289220
O	-2.331783	-2.669634	1.308442
C	-2.588806	0.552824	3.244657
O	-2.674206	0.562516	4.387205
C	1.032988	1.377809	0.322213
H	2.112532	1.386351	0.481228
H	0.830989	0.921468	-0.647849
H	0.698445	2.416349	0.277016

12*:

E = -1399.02885330913 au

ZPE = 0.22915547 au

C	0.741327	-1.598983	2.862927
C	1.590034	-2.187013	1.716664
H	1.332448	-1.087913	3.623398
H	2.647972	-1.933597	1.811329
H	1.499582	-3.266819	1.591742
C	-0.279938	-2.482537	3.499021
C	-0.600808	-2.305041	4.842700
C	-1.573241	-3.084733	5.451043
C	-2.241719	-4.058390	4.722564
C	-1.928170	-4.246726	3.383705
C	-0.957046	-3.464634	2.776805
H	-0.082818	-1.548101	5.419975
H	-1.806210	-2.932735	6.496977
H	-3.000188	-4.668491	5.195265
H	-2.441421	-5.004873	2.806527
H	-0.726481	-3.635268	1.732292
P	0.119312	-0.429664	1.547688
O	0.990885	-1.495521	0.599772
Mo	-2.190529	0.039987	0.856903
C	-4.041110	0.459045	0.197796
O	-5.089335	0.703787	-0.188688
C	-1.419967	0.892939	-0.819541
O	-0.999896	1.380192	-1.762135
C	-2.104423	1.835848	1.811300
O	-2.062931	2.843468	2.345070
C	-2.247213	-1.770724	-0.083416
O	-2.289223	-2.776792	-0.617628
C	-3.023640	-0.790186	2.539282
O	-3.558348	-1.206295	3.453791
C	1.221118	1.010441	1.735543
H	2.251960	0.716532	1.933414
H	1.191141	1.624646	0.835847
H	0.865662	1.615970	2.570554

TS(**7**→**13**):

E = -2328.28977241722 au

ZPE = 0.44125351 au

ν = -603.07 cm^{-1}

Li	-2.487688	0.089422	4.645669	C	0.840216	-3.380479	5.327050
O	-3.372452	1.873106	4.910587	C	-0.133129	-4.091801	6.015851
C	-2.535030	2.583386	5.808790	C	-1.460702	-3.998819	5.620503
H	-1.708014	2.971324	5.212163	C	-1.813315	-3.194585	4.545946
H	-3.053286	3.437391	6.254953	H	1.249331	-2.036952	3.709192
C	-2.009320	1.672269	6.898848	H	1.878070	-3.451518	5.625324
H	-2.797635	1.395989	7.611370	H	0.142315	-4.722189	6.851098
H	-1.231351	2.198923	7.463311	H	-2.223041	-4.562288	6.142746
O	-1.489393	0.513034	6.274895	H	-2.850938	-3.151690	4.242510
C	-0.927391	-0.417502	7.181109	P	-2.769930	-3.965074	1.054944
H	-1.666330	-0.756504	7.913282	C	-2.496581	-1.582665	2.091030
H	-0.580364	-1.273420	6.606457	H	-2.626530	-1.178699	1.099089
H	-0.079101	0.018531	7.714994	H	-3.379669	-1.815882	2.662905
C	-4.708646	1.654478	5.343806	C	-1.040421	-4.612798	1.008288
H	-4.767940	1.606356	6.436401	H	-0.357200	-3.866371	0.601810
H	-5.357571	2.472652	5.016848	H	-0.735571	-4.824000	2.034856
C	-5.168247	0.351672	4.725777	H	-0.943562	-5.530647	0.429080
H	-6.189122	0.120879	5.045901	Cl	-2.982005	-3.631374	-1.053209
H	-5.173248	0.431239	3.636591	Mo	-4.405553	-5.815655	1.699703
O	-4.273570	-0.700290	5.055192	C	-5.657083	-7.287336	2.152322
C	-4.534058	-1.322151	6.300832	O	-6.373781	-8.152613	2.403058
H	-5.526109	-1.779336	6.306439	C	-3.576643	-6.856448	0.153844
H	-3.789045	-2.102620	6.441511	O	-3.095861	-7.418060	-0.716093
H	-4.465849	-0.623442	7.139760	C	-3.009154	-6.559925	2.971525
O	-1.737590	-0.325979	3.008787	O	-2.227504	-6.981362	3.692816
C	-1.181590	-1.578256	2.698949	C	-5.149091	-4.660748	3.186690
H	-0.348290	-1.519029	1.993390	O	-5.577169	-4.007729	4.026144
C	-0.845295	-2.476901	3.848084	C	-5.749468	-4.979793	0.416789
C	0.483653	-2.581155	4.249820	O	-6.508427	-4.504996	-0.292682

13:

E = -2328.36921192351 au

ZPE = 0.44346975 au

Li	-2.722496	0.076085	4.671249	H	-4.106698	-1.605916	6.907658
O	-3.156098	2.074195	4.963425	H	-4.234403	0.036144	7.554211
C	-2.035906	2.610279	5.644652	O	-2.222007	-0.550052	3.118205
H	-1.258900	2.747355	4.890933	C	-1.856836	-1.772024	2.669579
H	-2.256392	3.591242	6.077621	H	-0.902102	-1.749103	2.103323
C	-1.546594	1.668968	6.726567	C	-1.634251	-2.746436	3.819810
H	-2.239892	1.634341	7.577957	C	-0.360352	-3.208337	4.134663
H	-0.585792	2.034304	7.109238	C	-0.140175	-4.010467	5.248781
O	-1.414754	0.388566	6.149172	C	-1.200083	-4.365245	6.069132
C	-0.907608	-0.590137	7.031847	C	-2.478407	-3.907587	5.769572
H	-1.555205	-0.716237	7.905450	C	-2.687576	-3.099461	4.662372
H	-0.859329	-1.531954	6.489428	H	0.476969	-2.927752	3.504431
H	0.096236	-0.327353	7.378023	H	0.860727	-4.356164	5.474849
C	-4.400357	2.186980	5.635752	H	-1.034498	-4.993166	6.935138
H	-4.264988	2.215046	6.722367	H	-3.312863	-4.180135	6.404203
H	-4.911532	3.111999	5.349540	H	-3.683612	-2.726997	4.455454
C	-5.239219	0.993035	5.233355	P	-2.622624	-3.923440	0.869891
H	-6.207957	1.027563	5.743620	C	-2.922129	-2.288498	1.657377
H	-5.429825	1.014575	4.158341	H	-2.981351	-1.549582	0.855869
O	-4.558149	-0.220383	5.498750	H	-3.904544	-2.332048	2.133556
C	-4.659036	-0.670913	6.834896	C	-0.828343	-3.961747	0.541421
H	-5.701762	-0.855715	7.107668	H	-0.477531	-3.025743	0.110323

H	-0.300670	-4.147196	1.476651
H	-0.604874	-4.778681	-0.142222
Cl	-3.273111	-3.526264	-1.087703
Mo	-3.642560	-6.072987	1.639947
C	-4.386851	-7.844544	2.177152
O	-4.806964	-8.866714	2.480110
C	-2.621006	-6.976657	0.127789
O	-2.067711	-7.499774	-0.721974
C	-2.061745	-6.374864	2.898837
O	-1.181668	-6.601492	3.585965
C	-4.755794	-5.213508	3.116399
O	-5.444888	-4.792765	3.922710
C	-5.221151	-5.779500	0.382979
O	-6.115031	-5.631566	-0.308665

TS(**13**→**8·12**):

E = -2328.35201775373 au
ZPE = 0.44251143 au
ν = -105.76 cm^{-1}

Li	-4.205578	-1.375421	5.526522
O	-4.878467	0.068966	6.896730
C	-5.303865	-0.658267	8.033885
H	-4.397792	-0.993839	8.539175
H	-5.859160	-0.025089	8.733860
C	-6.153967	-1.849136	7.640067
H	-7.154590	-1.541838	7.308043
H	-6.291197	-2.493042	8.517254
O	-5.487840	-2.536401	6.601912
C	-6.169175	-3.699454	6.183909
H	-7.167181	-3.464009	5.800276
H	-5.588620	-4.161276	5.388390
H	-6.271205	-4.416048	7.003877
C	-5.867145	0.896342	6.304783
H	-6.866379	0.459448	6.407723
H	-5.891093	1.878601	6.788533
C	-5.503388	1.046696	4.844964
H	-6.263910	1.643651	4.329854
H	-4.551160	1.570302	4.747538
O	-5.336635	-0.219958	4.237928
C	-6.527048	-0.811217	3.759552
H	-6.999996	-0.182815	2.999448
H	-6.257517	-1.762842	3.304326
H	-7.256148	-1.000318	4.553950
O	-2.964644	-2.232521	4.460455
C	-3.064769	-3.433349	3.774278
H	-4.024410	-3.940309	3.972605
C	-2.972530	-3.296299	2.268940
C	-2.683541	-4.397781	1.467105
C	-2.645253	-4.283741	0.084952
C	-2.906006	-3.061773	-0.520926
C	-3.212856	-1.962958	0.268955
C	-3.248208	-2.079971	1.652255
H	-2.480577	-5.360764	1.922468
H	-2.409004	-5.150111	-0.520073
H	-2.872986	-2.969054	-1.598956
H	-3.424722	-1.006804	-0.193594
H	-3.494760	-1.220460	2.259927
P	-1.112718	-2.763189	5.228022
C	-1.931810	-4.219118	4.433754
H	-1.236556	-4.775880	3.800036
H	-2.297857	-4.901531	5.204178
C	0.026324	-3.769984	6.314194
H	-0.523327	-4.443431	6.973651
H	0.657789	-4.378665	5.662293
H	0.670313	-3.135005	6.920845
Mo	0.357679	-1.066427	4.031342
Cl	-2.040019	-1.941161	7.028069
C	0.113054	-2.002812	2.229260
O	0.078310	-2.513629	1.210406
C	1.491363	0.299722	3.150864
O	2.147311	1.097727	2.645001
C	0.663823	-0.189860	5.838045
O	0.856405	0.306221	6.848961
C	2.010225	-2.205226	4.319750
O	2.969771	-2.811282	4.462323
C	-1.280963	0.122714	3.750389
O	-2.146332	0.850611	3.601722

8·12:

E = -2328.39641730517 au
ZPE = 0.44328979 au

Li	-3.819822	-1.237593	5.715444
O	-5.137553	0.290194	6.583581
C	-5.674455	-0.336025	7.726939
H	-4.871459	-0.353418	8.465266
H	-6.509838	0.231617	8.151789
C	-6.127101	-1.749321	7.428080
H	-7.013212	-1.762153	6.778692
H	-6.411947	-2.236701	8.368710
O	-5.057703	-2.434950	6.809285
C	-5.335982	-3.796574	6.573419
H	-6.153230	-3.925774	5.856192
H	-4.436763	-4.257573	6.170834
H	-5.602246	-4.314375	7.499628
C	-6.063133	0.786717	5.639314
H	-7.006070	0.228380	5.660383
H	-6.306373	1.835667	5.842389
C	-5.398192	0.662306	4.285598
H	-6.071237	1.014360	3.495737
H	-4.498451	1.280439	4.253466
O	-4.986077	-0.671185	4.057459
C	-6.015088	-1.506329	3.570208
H	-6.362625	-1.166312	2.590279

H	-5.609595	-2.508434	3.457228
H	-6.874782	-1.554621	4.246340
O	-2.563273	-2.551717	4.638895
C	-2.732698	-3.672512	3.739411
H	-3.388451	-4.415090	4.203374
C	-3.295360	-3.268435	2.410736
C	-4.042668	-4.194062	1.688341
C	-4.546731	-3.872486	0.436500
C	-4.313445	-2.614201	-0.102012
C	-3.571795	-1.686360	0.616791
C	-3.063309	-2.010667	1.865862
H	-4.237655	-5.173965	2.109402
H	-5.127608	-4.601190	-0.113831
H	-4.710344	-2.356765	-1.075376
H	-3.390297	-0.701969	0.205162
H	-2.505625	-1.269363	2.421295
P	-0.905591	-2.785957	4.899261
C	-1.268864	-4.152240	3.710842
H	-0.783797	-4.060886	2.741840
H	-1.112433	-5.158065	4.096833
C	-0.767023	-3.669938	6.483370
H	-1.579984	-4.384813	6.613262
H	0.178481	-4.215802	6.472428
H	-0.768386	-2.963787	7.308185
Mo	0.805071	-1.092539	4.292561
Cl	-2.016935	-0.566177	6.935226
C	0.543680	-1.485920	2.324159
O	0.432999	-1.700269	1.206196
C	2.226606	0.226637	3.814898
O	3.047201	0.978087	3.538512
C	1.118047	-0.696472	6.285820
O	1.374396	-0.476204	7.372553
C	2.190753	-2.565952	4.381957
O	2.976633	-3.396029	4.420507
C	-0.632545	0.374571	4.181817
O	-1.406653	1.199130	4.052954

12:

E = -1399.02923269544 au

ZPE = 0.22859063 au

O	-2.364065	-2.316063	4.195066
C	-2.675051	-3.503531	3.428105
H	-3.404315	-4.113382	3.969625
C	-3.206342	-3.167309	2.069130
C	-4.243998	-3.918704	1.528882
C	-4.713512	-3.657650	0.248204
C	-4.153535	-2.632639	-0.500333
C	-3.121711	-1.872239	0.036928
C	-2.649619	-2.138880	1.312684
H	-4.691598	-4.714487	2.112660
H	-5.521785	-4.250347	-0.159743
H	-4.521017	-2.422727	-1.496251
H	-2.684072	-1.067776	-0.539569
H	-1.848702	-1.532769	1.717323
P	-0.823983	-2.806555	4.625073
C	-1.270893	-4.153837	3.457104
H	-0.744094	-4.113677	2.505531
H	-1.229187	-5.167669	3.849720
C	-1.025327	-3.563596	6.268740
H	-1.861472	-4.262138	6.297849
H	-0.109546	-4.099023	6.523135
H	-1.182057	-2.789228	7.019213
Mo	1.019203	-1.201031	4.338744
C	1.213007	-1.781730	2.396130
O	1.323112	-2.102659	1.307002
C	2.525536	0.110416	4.105515
O	3.380449	0.857989	3.973182
C	0.812135	-0.624593	6.283777
O	0.702693	-0.292410	7.369058
C	2.333971	-2.649993	4.906635
O	3.070024	-3.460523	5.226768
C	-0.332375	0.225460	3.778747
O	-1.083708	1.021476	3.463287

TS(**7**→**13***):

E = -2328.29057803004 au

ZPE = 0.44096569 au

ν = -547.63 cm^{-1}

Li	-3.471792	-0.080260	4.792739
O	-5.342090	0.681595	4.661645
C	-5.226682	2.042259	5.042997
H	-4.838485	2.571237	4.171252
H	-6.199500	2.477651	5.290651
C	-4.279122	2.200294	6.214058
H	-4.719257	1.818661	7.144721
H	-4.069627	3.265156	6.367046
O	-3.095441	1.486838	5.909064
C	-2.110559	1.565007	6.922178
H	-2.488664	1.183202	7.875343
H	-1.262850	0.959423	6.609792
H	-1.775945	2.595669	7.065867
C	-6.209825	-0.117611	5.453317
H	-6.266391	0.256351	6.481280
H	-7.225129	-0.104367	5.045091
C	-5.661215	-1.528043	5.427071
H	-6.281224	-2.185780	6.044590
H	-5.676397	-1.920037	4.407892
O	-4.307059	-1.547072	5.852136
C	-4.136042	-1.608997	7.256351
H	-4.575805	-2.524079	7.660639
H	-3.066645	-1.615942	7.457303
H	-4.580274	-0.752468	7.772292

O	-2.626146	-0.532866	3.224191
C	-2.339485	-1.857745	2.874354
H	-2.519122	-2.121943	1.830466
H	-2.735649	-2.628707	3.538217
C	-0.450524	-1.211322	4.518021
C	0.544016	-0.261760	4.755956
C	1.165938	-0.181748	5.992070
C	0.797177	-1.046329	7.014529
C	-0.203597	-1.984320	6.792105
C	-0.825868	-2.066166	5.556119
H	0.842464	0.407817	3.958389
H	1.941268	0.555093	6.156384
H	1.285868	-0.990634	7.978270
H	-0.493954	-2.665138	7.581835
H	-1.590589	-2.814700	5.398488
P	0.640413	-3.292442	2.673311
C	-1.042376	-1.293404	3.174650
H	-0.547843	-0.752955	2.379362
C	2.266834	-2.741626	3.352734
H	2.499376	-1.726627	3.028541
H	2.190001	-2.732966	4.441356
H	3.089557	-3.398921	3.072998
Mo	0.281022	-5.773480	3.186759
C	2.101153	-6.025704	2.298619
O	3.119181	-6.152959	1.798076
C	1.156326	-5.468276	4.992496
O	1.635404	-5.290915	6.016314
C	0.111509	-7.717754	3.532808
O	0.034764	-8.849705	3.728121
C	-1.529616	-5.455701	4.030915
O	-2.562717	-5.315493	4.509019
C	-0.634670	-5.930983	1.373980
O	-1.164518	-6.012756	0.365344
Cl	1.164323	-3.152285	0.596416

13*: E = -2328.36621330987 au
ZPE = 0.44348988 au

Li	-3.555740	-0.348422	4.735584
O	-5.446802	0.522797	4.610368
C	-5.246900	1.908560	4.818281
H	-4.866093	2.306096	3.876102
H	-6.185515	2.425865	5.042045
C	-4.245838	2.159438	5.927678
H	-4.664527	1.913992	6.913211
H	-3.988328	3.225467	5.943941
O	-3.111840	1.362132	5.665216
C	-2.074050	1.509387	6.610800
H	-2.412675	1.259021	7.621348
H	-1.272737	0.829504	6.329919
H	-1.686404	2.532306	6.617526
C	-6.307286	-0.124867	5.532757
H	-6.311026	0.386366	6.501598
H	-7.338488	-0.125848	5.164267
C	-5.817180	-1.549272	5.683310
H	-6.449139	-2.087942	6.397943
H	-5.881257	-2.070393	4.725901
O	-4.455503	-1.582143	6.071080
C	-4.238659	-1.415493	7.457024
H	-4.715318	-2.217785	8.027522
H	-3.164710	-1.454752	7.627155
H	-4.611000	-0.457558	7.833588
O	-2.707771	-0.873127	3.324564
C	-1.942897	-1.889225	2.879866
H	-1.986164	-2.028918	1.780493
H	-2.228258	-2.883360	3.286148
C	-0.139292	-1.476930	4.672205
C	0.857184	-0.578942	5.055472
C	1.214010	-0.425249	6.387472
C	0.573422	-1.169417	7.368792
C	-0.437068	-2.048341	7.004593
C	-0.794697	-2.194735	5.671329
H	1.362351	0.009689	4.298655
H	1.991670	0.277655	6.657253
H	0.852007	-1.057966	8.408579
H	-0.950468	-2.626581	7.762545
H	-1.605213	-2.860934	5.414011
P	0.593040	-3.107977	2.629817
C	-0.443699	-1.684170	3.212465
H	-0.075498	-0.817986	2.651814
C	2.307209	-2.542313	2.904414
H	2.447139	-1.517392	2.564297
H	2.543428	-2.601597	3.965882
H	2.993362	-3.192803	2.365021
Mo	0.215228	-5.519781	3.193023
C	1.893677	-5.883533	2.095818
O	2.823158	-6.130653	1.482756
C	1.310295	-5.229068	4.886634
O	1.911921	-5.068290	5.842206
C	0.117797	-7.479338	3.555608
O	0.079778	-8.606191	3.756599
C	-1.501974	-5.344207	4.278005
O	-2.468143	-5.359459	4.885698
C	-0.938371	-5.717703	1.517320
O	-1.594063	-5.830891	0.593099
Cl	0.507812	-2.813460	0.559912

TS(**13*→8·12***): E = -2328.35205466299 au
ZPE = 0.44309915 au
ν = -83.13 cm^{-1}

Li	-3.220404	0.108493	4.285040
O	-4.454066	1.778147	4.588675

C	-3.825943	2.535109	5.606406
H	-2.971369	3.026189	5.140099
H	-4.487072	3.316549	5.995148
C	-3.359941	1.648597	6.743147
H	-4.202722	1.284911	7.346314
H	-2.717452	2.235296	7.410772
O	-2.655482	0.560084	6.184305
C	-2.099992	-0.300631	7.154397
H	-2.873988	-0.747631	7.786670
H	-1.571005	-1.096310	6.634174
H	-1.390298	0.229864	7.795585
C	-5.786407	1.378521	4.864974
H	-5.942286	1.227323	5.938658
H	-6.496966	2.145595	4.539646
C	-6.031671	0.092730	4.107175
H	-7.036093	-0.285443	4.326657
H	-5.974533	0.272527	3.032410
O	-5.036951	-0.866864	4.411918
C	-5.275606	-1.609147	5.590732
H	-6.206815	-2.177649	5.514457
H	-4.449993	-2.308607	5.709696
H	-5.327555	-0.980478	6.485217
O	-2.107588	-1.054751	3.365827
C	-1.379884	-2.147244	3.826632
H	-1.508837	-3.048394	3.211669
H	-1.634584	-2.437590	4.854636
C	1.209559	-2.442790	3.412709
C	2.410959	-2.250854	4.094674
C	3.530757	-3.015903	3.803249
C	3.467326	-3.995139	2.820889
C	2.276417	-4.201229	2.137714
C	1.159951	-3.432762	2.431894
H	2.469291	-1.489093	4.863453
H	4.452687	-2.848708	4.345230
H	4.338546	-4.594540	2.590875
H	2.215101	-4.962499	1.370865
H	0.241646	-3.611891	1.889040
P	-0.478807	-0.320304	2.414496
C	0.029333	-1.581135	3.713615
H	0.239259	-0.982962	4.608544
C	1.183530	0.527793	2.308541
H	1.553363	0.837940	3.287474
H	1.895890	-0.184279	1.886796
H	1.149099	1.400497	1.659819
Mo	-1.260193	-0.789062	0.038934
C	-0.654973	1.088689	-0.459812
O	-0.344932	2.141481	-0.776336
C	0.567789	-1.538164	-0.450460
O	1.589428	-1.952145	-0.750113
C	-1.890754	-1.038767	-1.825479
O	-2.255168	-1.174907	-2.907282
C	-1.963060	-2.650008	0.465435
O	-2.403676	-3.691566	0.633100
C	-3.057698	-0.032580	0.631268
O	-4.063168	0.392294	0.963627
Cl	-1.339842	1.575212	3.132938

8·12*:

E = -2328.39221809176 au

ZPE = 0.44351813 au

Li	-3.079846	0.692138	4.150937
O	-4.974119	1.655578	4.478661
C	-4.708907	2.607398	5.486770
H	-4.131101	3.398922	5.007557
H	-5.628847	3.052127	5.881615
C	-3.909207	2.001256	6.620742
H	-4.509166	1.290942	7.206172
H	-3.599423	2.800346	7.305410
O	-2.787973	1.350634	6.061001
C	-1.910935	0.815039	7.025493
H	-2.386507	0.019179	7.608397
H	-1.050414	0.404504	6.501777
H	-1.559573	1.587466	7.715977
C	-6.026519	0.747795	4.732025
H	-6.141518	0.547973	5.803494
H	-6.982774	1.146711	4.375572
C	-5.689621	-0.522498	3.982842
H	-6.467070	-1.276571	4.150601
H	-5.647148	-0.323140	2.910529
O	-4.413155	-1.007851	4.356249
C	-4.417492	-1.730382	5.570288
H	-5.107436	-2.578117	5.519503
H	-3.414003	-2.115154	5.737067
H	-4.693426	-1.114835	6.432065
O	-1.455130	-0.500078	3.844566
C	-1.023702	-1.773725	4.365995
H	-1.652934	-2.571162	3.966225
H	-1.084182	-1.788128	5.455063
C	0.956741	-2.997240	3.152407
C	2.153790	-2.894147	2.444055
C	2.708568	-3.996449	1.815623
C	2.081470	-5.233666	1.899957
C	0.904320	-5.353146	2.621549
C	0.345492	-4.243006	3.242229
H	2.661983	-1.938552	2.389217
H	3.633113	-3.890736	1.263025
H	2.512130	-6.097581	1.410878
H	0.412352	-6.313809	2.702402
H	-0.575917	-4.366067	3.796882
P	-0.154227	-0.309178	2.780555
C	0.401935	-1.775450	3.806648
H	1.108219	-1.414368	4.559956
C	0.830840	1.067035	3.440984
H	0.870985	1.042169	4.529890
H	1.844535	0.965098	3.047751
H	0.411150	2.014131	3.113806
Mo	-0.487245	-0.464354	0.323707
C	-0.350176	1.577351	0.192307
O	-0.218914	2.696556	0.027825
C	1.511205	-0.574898	0.012072

O	2.633845	-0.626630	-0.201189
C	-0.782401	-0.522900	-1.648523
O	-0.953678	-0.553021	-2.781831
C	-0.606914	-2.495467	0.324983
O	-0.700101	-3.628685	0.220042
C	-2.515192	-0.366230	0.637149
O	-3.645872	-0.367080	0.769601
Cl	-2.297749	2.205769	2.640178

12*: E = -1399.02878809581 au

ZPE = 0.22915944 au

O	-1.177374	-0.575497	3.702100
C	-0.457020	-1.428313	4.617675
H	-1.042841	-2.317598	4.853739
H	-0.231061	-0.901642	5.547159
C	0.965898	-3.049742	3.164967
C	2.243645	-3.568243	2.969009
C	2.425277	-4.805956	2.370162
C	1.327586	-5.547647	1.957238
C	0.049208	-5.042820	2.150320
C	-0.130058	-3.804104	2.747485
H	3.106800	-2.996542	3.289355
H	3.426426	-5.192016	2.228784
H	1.466715	-6.514053	1.490695
H	-0.814194	-5.614016	1.834690
H	-1.138132	-3.435397	2.892789
P	0.114157	-0.365730	2.662045
C	0.806288	-1.696155	3.773578
H	1.729846	-1.384894	4.262507
C	0.944671	1.138451	3.271329
H	1.019722	1.154605	4.358688
H	1.951480	1.175663	2.852687
H	0.406631	2.025144	2.936877
Mo	-0.369016	-0.453416	0.255716
C	-1.494818	1.219686	0.510550
O	-2.125310	2.162203	0.638940
C	1.266612	0.702232	-0.108504
O	2.181705	1.350283	-0.319523
C	-0.830882	-0.446423	-1.699404
O	-1.100912	-0.433025	-2.810641
C	0.769664	-2.131273	-0.064115
O	1.400490	-3.041790	-0.325624
C	-1.999103	-1.615603	0.652805
O	-2.916017	-2.259786	0.861730

TS(**7**→**13′**): E = -2328.28885221403 au

ZPE = 0.4408262 au

ν = -604.96 cm^{-1}

Li	-3.081879	-0.161716	5.045852
O	-4.118504	1.517864	5.409338
C	-3.160459	2.563943	5.403322
H	-2.946046	2.773042	4.354284
H	-3.559079	3.479080	5.851348
C	-1.893401	2.149516	6.122392
H	-2.041340	2.097036	7.209016
H	-1.117066	2.900907	5.937667
O	-1.505129	0.883366	5.623234
C	-0.283114	0.418212	6.166167
H	-0.345250	0.314623	7.253647
H	-0.071672	-0.554921	5.729091
H	0.536123	1.100569	5.925353
C	-4.852910	1.352998	6.614294
H	-4.263048	1.660411	7.484419
H	-5.758808	1.966822	6.601060
C	-5.223206	-0.111310	6.707992
H	-5.763090	-0.305420	7.640072
H	-5.878724	-0.388630	5.880151
O	-4.066274	-0.929137	6.602690
C	-3.367942	-1.115891	7.821653
H	-4.004340	-1.606870	8.561884
H	-2.511758	-1.755609	7.616058
H	-3.002603	-0.175793	8.245080
O	-3.091203	-0.872994	3.341094
C	-2.058265	-1.641028	2.789243
H	-1.534742	-1.162260	1.957495
C	-1.103389	-2.296669	3.735613
C	0.250444	-2.348438	3.426844
C	1.150017	-2.968549	4.284814
C	0.702037	-3.533356	5.469637
C	-0.650567	-3.484625	5.787079
C	-1.547030	-2.873295	4.924677
H	0.608036	-1.901341	2.506801
H	2.200353	-3.005141	4.027760
H	1.400737	-4.013562	6.142107
H	-1.007644	-3.933266	6.705327
H	-2.601367	-2.868932	5.178012
P	-2.431101	-4.477866	1.220258
C	-3.284743	-2.328026	2.442431
H	-3.833222	-2.046751	1.557179
H	-3.741285	-3.018816	3.135322
Cl	-4.226746	-5.458876	0.566601
C	-2.056825	-5.610926	2.631619
H	-1.152152	-5.243348	3.119681
H	-2.861188	-5.594682	3.368197
H	-1.884160	-6.640601	2.319337
Mo	-0.696039	-4.776727	-0.627326
C	-1.237262	-6.742053	-0.595601
O	-1.555436	-7.837934	-0.553698
C	-0.272849	-2.798756	-0.539905
O	-0.033727	-1.679011	-0.491958

C	0.718952	-5.133533	0.783552
O	1.523244	-5.331608	1.572864
C	0.628291	-5.055939	-2.077922
O	1.394606	-5.234180	-2.918561
C	-2.178767	-4.384250	-1.968258
O	-3.007853	-4.154338	-2.719812

13′:

E = -2328.37172022569 au

ZPE = 0.44338588 au

Li	-3.014909	-0.216515	4.890119
O	-3.971960	1.575506	5.203398
C	-2.947171	2.553390	5.210657
H	-2.671189	2.711137	4.166862
H	-3.302224	3.509186	5.609133
C	-1.741779	2.084632	5.999994
H	-1.943823	2.072017	7.079667
H	-0.914947	2.786419	5.836340
O	-1.410692	0.789069	5.548956
C	-0.257998	0.250261	6.162520
H	-0.380506	0.174965	7.247787
H	-0.099527	-0.745552	5.754493
H	0.623007	0.863926	5.953619
C	-4.754585	1.490298	6.383582
H	-4.184763	1.807432	7.263654
H	-5.631402	2.142341	6.313799
C	-5.197793	0.049872	6.525307
H	-5.790289	-0.070986	7.438710
H	-5.829358	-0.232670	5.680504
O	-4.086198	-0.828748	6.510134
C	-3.429050	-0.967469	7.754328
H	-4.107131	-1.369824	8.511976
H	-2.606120	-1.665933	7.613733
H	-3.018946	-0.023081	8.125070
O	-3.048911	-1.039263	3.345772
C	-2.280997	-2.018710	2.818567
H	-1.677285	-1.672240	1.955269
C	-1.282587	-2.588817	3.816339
C	0.080682	-2.608109	3.545653
C	0.987426	-3.136858	4.459075
C	0.536629	-3.645587	5.667801
C	-0.824302	-3.616941	5.957807
C	-1.721773	-3.090838	5.042004
H	0.442731	-2.202295	2.607486
H	2.044630	-3.145329	4.226076
H	1.237576	-4.058398	6.381875
H	-1.183735	-4.013215	6.899613
H	-2.780400	-3.083174	5.276095
P	-2.338168	-4.579413	1.471227
C	-3.181013	-3.157856	2.264707
H	-3.834575	-2.731973	1.498856
H	-3.817709	-3.551247	3.062646
Cl	-4.043343	-5.668168	0.909504
C	-1.771087	-5.663238	2.824016
H	-0.860408	-5.255004	3.259870
H	-2.526835	-5.754646	3.602184
H	-1.546754	-6.648120	2.416742
Mo	-0.820346	-4.399090	-0.503025
C	-1.079868	-6.411223	-0.715956
O	-1.208091	-7.535260	-0.856541
C	-0.512281	-2.386176	-0.393153
O	-0.293961	-1.266305	-0.411168
C	0.793828	-4.729895	0.694782
O	1.708651	-4.921396	1.348913
C	0.360307	-4.413278	-2.114305
O	1.034469	-4.434530	-3.039862
C	-2.464347	-4.047101	-1.663965
O	-3.376005	-3.843541	-2.316304

TS(**13′**→**8**·**12′**):

E = -2328.35592368699 au

ZPE = 0.44331711 au

ν = -105.78 cm^{-1}

Li	-3.315027	-1.493761	5.226597
O	-4.929168	-0.283179	5.426676
C	-4.618316	0.910444	4.734859
H	-4.684682	0.671719	3.673141
H	-5.347449	1.700874	4.941035
C	-3.223598	1.396330	5.077560
H	-3.187337	1.844629	6.079696
H	-2.933658	2.178936	4.365817
O	-2.349807	0.290781	5.009728
C	-1.008305	0.624681	5.296306
H	-0.915134	1.088012	6.284041
H	-0.423488	-0.291923	5.279351
H	-0.604007	1.314555	4.549741
C	-5.262605	-0.130636	6.796511
H	-4.755201	0.735128	7.235770
H	-6.339106	0.029553	6.916147
C	-4.852539	-1.404471	7.503451
H	-5.045129	-1.314969	8.577974
H	-5.437002	-2.244907	7.127819
O	-3.491232	-1.708982	7.247088
C	-2.576564	-1.047855	8.099526
H	-2.730967	-1.343632	9.140963
H	-1.573289	-1.342850	7.797420
H	-2.649858	0.042075	8.035497
O	-2.893687	-2.629842	3.819538
C	-1.673126	-2.995625	3.238401
H	-1.453970	-2.411655	2.332763
C	-0.512001	-2.844974	4.186577
C	0.698637	-2.321253	3.747031
C	1.770499	-2.175615	4.619328

C	1.639373	-2.548414	5.949413
C	0.434179	-3.077837	6.396744
C	-0.632068	-3.227641	5.521940
H	0.802446	-2.011562	2.713337
H	2.704206	-1.761605	4.260683
H	2.469385	-2.429249	6.633662
H	0.325726	-3.377388	7.431710
H	-1.564212	-3.647872	5.882869
P	-3.845230	-4.177188	3.109542
C	-2.019288	-4.435101	2.872585
H	-1.638935	-5.154133	3.603263
H	-1.743581	-4.790056	1.877877
Cl	-4.741771	-4.118259	5.137123
C	-4.275121	-5.997425	3.057477
H	-3.989349	-6.365790	2.067971
H	-3.726378	-6.569320	3.807431
H	-5.339595	-6.172715	3.199554
Mo	-5.226839	-3.007303	1.317691
C	-6.501280	-4.582193	1.171220
O	-7.248628	-5.438608	1.048583
C	-3.995907	-1.383372	1.411006
O	-3.344544	-0.445409	1.405365
C	-4.003038	-3.865642	-0.051217
O	-3.305616	-4.357392	-0.813005
C	-6.267576	-2.087489	-0.098208
O	-6.866764	-1.554267	-0.922806
C	-6.407773	-2.227288	2.792831
O	-7.089605	-1.804909	3.603088

8·12′:

E = -2328.39805777522 au

ZPE = 0.44362513 au

Li	-4.001626	-1.552812	5.311215
O	-5.207710	-0.419942	6.705680
C	-5.776554	0.600348	5.916076
H	-6.518210	0.106913	5.287228
H	-6.294082	1.350540	6.524708
C	-4.737064	1.281415	5.049202
H	-4.081952	1.938618	5.637166
H	-5.250952	1.915073	4.316079
O	-3.972314	0.281782	4.411412
C	-3.055685	0.801492	3.477224
H	-2.324600	1.462599	3.954513
H	-2.528123	-0.033760	3.023908
H	-3.565084	1.361163	2.686809
C	-4.452098	-0.009618	7.825782
H	-3.945854	0.946512	7.649929
H	-5.092049	0.121186	8.705873
C	-3.443184	-1.108484	8.078516
H	-2.807433	-0.852690	8.933417
H	-3.960094	-2.040656	8.316745
O	-2.672012	-1.353984	6.917806
C	-1.569376	-0.484393	6.771231
H	-0.882089	-0.579096	7.617023
H	-1.035621	-0.767938	5.867244
H	-1.867684	0.564849	6.680911
O	-2.731899	-2.589522	4.022256
C	-1.319857	-2.677922	3.737368
H	-0.932373	-1.676043	3.526885
C	-0.529025	-3.290139	4.857739
C	0.858799	-3.172289	4.818441
C	1.645296	-3.746004	5.804710
C	1.049764	-4.439839	6.851319
C	-0.331609	-4.551781	6.899142
C	-1.120450	-3.979672	5.908689
H	1.329720	-2.626496	4.008412
H	2.722194	-3.648007	5.759627
H	1.660177	-4.886253	7.625473
H	-0.803822	-5.085293	7.714100
H	-2.196179	-4.067425	5.971097
P	-3.239192	-3.554873	2.726294
C	-1.410556	-3.528735	2.455042
H	-0.917585	-4.497777	2.509376
H	-1.088002	-3.012651	1.553436
Cl	-5.653832	-3.027031	4.743975
C	-3.507146	-5.231426	3.382839
H	-3.422518	-5.919152	2.538621
H	-2.757195	-5.504325	4.124033
H	-4.498736	-5.313191	3.817159
Mo	-4.724651	-2.787900	0.888745
C	-6.144755	-4.112646	1.547750
O	-6.944752	-4.849836	1.882898
C	-3.312946	-1.457846	0.304717
O	-2.520190	-0.704911	-0.031154
C	-3.856229	-4.230489	-0.234401
O	-3.374833	-5.036731	-0.887214
C	-5.859760	-2.305857	-0.680792
O	-6.504826	-2.044227	-1.591836
C	-5.669938	-1.306154	1.962769
O	-6.247641	-0.455944	2.449533

12′:

E = -1399.02760632043 au

ZPE = 0.22842044 au

O	-3.121137	-3.254507	2.956142
C	-1.749846	-2.830804	3.087673
H	-1.670047	-1.782224	2.786319
C	-1.219292	-2.988441	4.485423
C	0.107636	-2.641383	4.734366
C	0.643444	-2.766762	6.005892
C	-0.143921	-3.238135	7.050276
C	-1.466203	-3.577518	6.809479
C	-2.004636	-3.452115	5.533357
H	0.729516	-2.271583	3.926724

H	1.676076	-2.495551	6.183073
H	0.272346	-3.336925	8.044218
H	-2.088015	-3.941553	7.617121
H	-3.039513	-3.716942	5.363797
P	-2.801195	-4.500458	1.882441
C	-1.119359	-3.776303	2.035626
H	-0.343518	-4.445101	2.403199
H	-0.769719	-3.270263	1.138138
C	-2.760449	-6.005771	2.902077
H	-2.301734	-6.804417	2.316934
H	-2.186421	-5.866774	3.817440
H	-3.776042	-6.310863	3.153680
Mo	-4.150315	-4.519392	-0.169615
C	-4.271780	-6.552198	-0.120547
O	-4.341100	-7.690441	-0.094739
C	-2.448079	-4.648320	-1.283913
O	-1.491322	-4.720338	-1.900329
C	-5.269667	-4.472863	-1.840547
O	-5.906004	-4.442302	-2.790040
C	-5.828594	-4.395738	0.984949
O	-6.767398	-4.329770	1.628277
C	-4.000689	-2.484557	-0.166627
O	-3.910219	-1.348309	-0.157347

TS(**7**→**13***′**):

E = -2328.28903087312 au
ZPE = 0.44076126 au
ν = -548.04 cm^{-1}

Li	-3.211853	-0.546018	4.771485
O	-4.984295	0.425530	4.915666
C	-4.658663	1.779207	5.183308
H	-4.370834	2.217838	4.226687
H	-5.521826	2.336834	5.559305
C	-3.512412	1.878286	6.168513
H	-3.824883	1.598965	7.183346
H	-3.163374	2.916436	6.211123
O	-2.485871	1.013515	5.721080
C	-1.332524	1.032301	6.540504
H	-1.570020	0.744559	7.569131
H	-0.620545	0.320539	6.129352
H	-0.874944	2.025033	6.551253
C	-5.779547	-0.229628	5.893113
H	-5.604161	0.184950	6.891740
H	-6.844045	-0.105213	5.671268
C	-5.412773	-1.697351	5.850704
H	-5.979690	-2.250043	6.606718
H	-5.661430	-2.119745	4.875012
O	-4.014433	-1.872283	6.025761
C	-3.592045	-1.886812	7.377749
H	-4.059732	-2.711165	7.921781
H	-2.513419	-2.030166	7.383044
H	-3.819796	-0.952667	7.899749
O	-2.645533	-1.046217	3.103932
C	-2.436669	-2.339124	2.613376
H	-2.777136	-2.517907	1.590959
H	-2.736858	-3.161147	3.265117
C	-0.309423	-1.833700	3.995622
C	0.830850	-1.033519	4.081301
C	1.617936	-1.038187	5.221454
C	1.271116	-1.838534	6.303013
C	0.133753	-2.632179	6.229812
C	-0.651802	-2.633831	5.086227
H	1.109627	-0.413730	3.237387
H	2.501873	-0.415515	5.266747
H	1.883254	-1.845155	7.195246
H	-0.139312	-3.264378	7.065162
H	-1.522039	-3.274932	5.044022
P	0.360791	-3.804983	1.753711
C	-1.098139	-1.806637	2.756369
H	-0.723608	-1.203047	1.942290
Mo	0.465015	-4.590226	-0.666171
C	0.532131	-5.228884	-2.544803
O	0.579571	-5.612644	-3.629088
C	2.382423	-3.937122	-0.775728
O	3.462938	-3.565015	-0.836344
C	1.115921	-6.366603	0.088580
O	1.486320	-7.346790	0.543031
C	-1.468182	-5.196922	-0.457121
O	-2.556524	-5.524568	-0.338850
C	-0.189348	-2.753638	-1.225991
O	-0.560324	-1.712365	-1.523351
Cl	-0.636127	-5.342378	2.866884
C	1.998200	-3.948224	2.588201
H	2.654045	-3.185644	2.163441
H	1.901893	-3.748884	3.656199
H	2.467737	-4.921201	2.444510

13*′**:

E = -2328.36542264907 au
ZPE = 0.44339182 au

Li	-3.184735	-0.813302	4.619205
O	-5.002297	0.216137	4.650520
C	-4.662039	1.589289	4.692987
H	-4.367571	1.860823	3.678005
H	-5.519042	2.212599	4.968355
C	-3.514941	1.843110	5.650082
H	-3.830207	1.734825	6.696900
H	-3.165687	2.875311	5.525030
O	-2.495612	0.916097	5.348143
C	-1.345451	1.043457	6.156555
H	-1.586417	0.929074	7.218441
H	-0.649764	0.258992	5.867124

H	-0.865059	2.015661	6.012228
C	-5.780087	-0.263521	5.734386
H	-5.605470	0.323512	6.642757
H	-6.848614	-0.192280	5.505441
C	-5.401039	-1.712548	5.954294
H	-5.970202	-2.124093	6.795060
H	-5.646129	-2.301486	5.068086
O	-4.006009	-1.851571	6.158212
C	-3.578250	-1.588598	7.479129
H	-4.040225	-2.283332	8.186406
H	-2.499122	-1.726038	7.506064
H	-3.803615	-0.567657	7.802897
O	-2.571249	-1.499907	3.162480
C	-1.942626	-2.571000	2.647931
H	-2.123885	-2.711772	1.562081
H	-2.251215	-3.537090	3.099257
C	0.074995	-2.274465	4.211228
C	1.142823	-1.416862	4.471385
C	1.636245	-1.250470	5.758124
C	1.058675	-1.937765	6.816332
C	-0.016811	-2.782246	6.573170
C	-0.505785	-2.948302	5.284837
H	1.597125	-0.870787	3.652343
H	2.468094	-0.580035	5.932398
H	1.438082	-1.812326	7.822165
H	-0.476772	-3.321030	7.392244
H	-1.348900	-3.605698	5.120746
P	0.459164	-3.880436	1.950073
C	-0.397737	-2.476714	2.801327
H	-0.060553	-1.623143	2.196264
Mo	0.105420	-4.246895	-0.489099
C	-0.143676	-4.593728	-2.442070
O	-0.282763	-4.796855	-3.560421
C	1.968358	-3.524691	-0.864082
O	3.012609	-3.122801	-1.089756
C	0.885242	-6.114994	-0.244155
O	1.328478	-7.157976	-0.119124
C	-1.764494	-4.981788	-0.100454
O	-2.803475	-5.404256	0.099109
C	-0.647263	-2.366528	-0.738716
O	-1.051892	-1.314177	-0.910025
Cl	-0.143168	-5.532801	3.069495
C	2.199853	-3.788858	2.479329
H	2.635697	-2.869130	2.087389
H	2.300233	-3.793892	3.562510
H	2.743242	-4.633650	2.058732

TS(**13*′**→**8**·**12*′**):

E = -2328.35398673776 au

ZPE = 0.443419 au

ν = -95.51 cm^{-1}

Li	-2.828395	-1.613158	4.357293
O	-4.479335	-0.425352	3.930743
C	-4.283802	0.798817	4.610108
H	-3.529313	1.344349	4.040552
H	-5.194465	1.407110	4.623110
C	-3.799600	0.572122	6.026497
H	-4.598431	0.184967	6.672417
H	-3.480077	1.531823	6.450413
O	-2.726699	-0.345074	5.975832
C	-2.137383	-0.572531	7.239825
H	-2.852124	-1.018850	7.938585
H	-1.303137	-1.254991	7.101386
H	-1.763351	0.359829	7.673134
C	-5.657253	-1.138219	4.267347
H	-5.901835	-1.030440	5.330137
H	-6.514403	-0.765970	3.695865
C	-5.397680	-2.588706	3.927259
H	-6.269330	-3.197679	4.188862
H	-5.229148	-2.696833	2.854781
O	-4.228386	-3.052015	4.578353
C	-4.446357	-3.568357	5.876775
H	-5.136898	-4.415494	5.848944
H	-3.487053	-3.912172	6.256213
H	-4.845440	-2.816687	6.565286
O	-1.570925	-1.651300	3.042124
C	-0.763827	-0.818468	2.278491
H	-0.870878	0.243737	2.536276
H	-0.957088	-0.901924	1.197624
C	1.383420	-0.644655	3.680105
C	2.774964	-0.585616	3.620969
C	3.509888	0.057948	4.607136
C	2.861591	0.659736	5.676262
C	1.475045	0.612365	5.744900
C	0.743382	-0.034737	4.760045
H	3.291128	-1.048015	2.787591
H	4.589452	0.092417	4.535640
H	3.429936	1.164950	6.446344
H	0.958507	1.081809	6.572848
H	-0.335341	-0.057204	4.835471
P	-0.055489	-3.040793	3.137153
C	0.622438	-1.364815	2.608269
H	1.281522	-1.517190	1.747038
Mo	-1.078481	-4.636259	1.433619
C	-1.878744	-5.859585	0.095752
O	-2.340389	-6.569494	-0.682683
C	0.351063	-6.036423	1.758665
O	1.127405	-6.865739	1.893043
C	-2.196553	-5.441486	2.942815
O	-2.807187	-5.912992	3.782666
C	-2.572984	-3.296833	1.021786
O	-3.435487	-2.618000	0.711724
C	0.094334	-3.769143	0.024829
O	0.762012	-3.277559	-0.763142
Cl	-0.789951	-3.217186	5.173482
C	1.564919	-3.786393	3.682984
H	2.215472	-3.840386	2.805892
H	2.070185	-3.196710	4.447855
H	1.418145	-4.795313	4.064583

8·12*′: E = -2328.3945100861 au

ZPE = 0.44381509 au

Li	-3.130025	-2.226130	4.912617
O	-3.937303	-0.092859	4.577622
C	-3.775409	0.598705	5.795247
H	-2.725077	0.893890	5.842250
H	-4.375984	1.514949	5.830170
C	-4.116567	-0.276033	6.981894
H	-5.198532	-0.449691	7.058631
H	-3.810941	0.240308	7.900746
O	-3.435663	-1.501267	6.833475
C	-3.624369	-2.366889	7.935535
H	-4.685671	-2.591301	8.086336
H	-3.094590	-3.289315	7.715309
H	-3.229447	-1.923908	8.855137
C	-5.268137	-0.235380	4.122638
H	-5.965492	-0.392675	4.953913
H	-5.598641	0.663180	3.588325
C	-5.284196	-1.426883	3.192552
H	-6.294773	-1.583184	2.799617
H	-4.623947	-1.243702	2.341609
O	-4.799334	-2.578738	3.850007
C	-5.778160	-3.342074	4.524785
H	-6.528036	-3.724414	3.826167
H	-5.259148	-4.178668	4.987048
H	-6.290606	-2.770599	5.305912
O	-1.609404	-1.749728	3.747881
C	-1.063577	-0.485441	3.311788
H	-0.881675	0.164844	4.168596
H	-1.755040	0.015202	2.635944
C	1.551700	-0.430541	2.921055
C	2.591506	-0.688491	2.026278
C	3.874672	-0.223213	2.265881
C	4.143172	0.522517	3.406490
C	3.116683	0.794650	4.298768
C	1.833403	0.320502	4.060120
H	2.391273	-1.259202	1.126723
H	4.664133	-0.438363	1.557654
H	5.142504	0.892245	3.594728
H	3.311511	1.379863	5.188061
H	1.055125	0.551330	4.776175
P	-0.252477	-2.672363	3.361209
C	0.207200	-1.009309	2.629935
H	0.064945	-1.057887	1.549247
Mo	-0.350558	-4.673330	1.913597
C	-0.357920	-6.293142	0.741617
O	-0.353359	-7.219149	0.066433
C	1.649878	-4.514452	1.652260
O	2.779908	-4.440572	1.491054
C	-0.036251	-5.860445	3.560074
O	0.178523	-6.555501	4.435296
C	-2.393309	-4.851901	2.154651
O	-3.520542	-4.987603	2.197162
C	-0.671435	-3.504638	0.292469
O	-0.850379	-2.858206	-0.634169
Cl	-2.334155	-4.343023	5.364647
C	0.716890	-2.764852	4.895159
H	1.747904	-3.011364	4.636697
H	0.709990	-1.829024	5.452329
H	0.302130	-3.554732	5.516788

12*′: E = -1399.02514827122 au

ZPE = 0.22900558 au

O	-1.996301	-2.032245	3.245313
C	-1.397983	-0.718293	3.294285
H	-1.412344	-0.326868	4.313043
H	-1.940921	-0.027253	2.649472
C	1.247636	-0.608932	3.459672
C	2.454965	-0.662826	2.761263
C	3.645728	-0.284054	3.360579
C	3.651567	0.167436	4.674067
C	2.457284	0.234932	5.376003
C	1.266305	-0.151912	4.775702
H	2.462840	-1.002941	1.732299
H	4.569038	-0.335773	2.798549
H	4.578570	0.468701	5.143808
H	2.447337	0.591481	6.397712
H	0.351599	-0.083232	5.350108
P	-0.557777	-2.844171	3.000799
C	0.006971	-1.081225	2.777786
H	0.077157	-0.883831	1.705868
Mo	-0.548292	-4.573321	1.261884
C	-0.605350	-5.985467	-0.171052
O	-0.642270	-6.787361	-0.984825
C	0.856756	-5.636334	2.284728
O	1.643804	-6.231956	2.856084
C	-2.023966	-5.540099	2.289094
O	-2.844626	-6.085836	2.861677
C	-1.952621	-3.465835	0.277328
O	-2.733220	-2.842562	-0.270843
C	0.926552	-3.593664	0.253853
O	1.749629	-3.043705	-0.313280
C	0.008628	-3.314919	4.663619
H	1.075122	-3.541319	4.624413
H	-0.152489	-2.532814	5.404225
H	-0.516197	-4.217033	4.977888

5^{tBu}:

E = -2061.84800838543 au
ZPE = 0.38472026 au

Li	-2.985788	-1.804356	4.473179
O	-3.933196	-0.176745	3.835875
C	-2.942282	0.819414	3.649376
H	-2.483028	0.615459	2.681254
H	-3.379507	1.821354	3.604526
C	-1.896706	0.755563	4.744649
H	-2.277912	1.144611	5.697810
H	-1.039844	1.375314	4.457599
O	-1.518551	-0.600196	4.887705
C	-0.419316	-0.805919	5.751029
H	-0.638536	-0.463739	6.766826
H	-0.212221	-1.874072	5.774654
H	0.469003	-0.284176	5.386730
C	-4.958618	0.127547	4.771100
H	-4.586425	0.777151	5.570985
H	-5.784596	0.651771	4.280591
C	-5.443960	-1.191480	5.331846
H	-6.224552	-1.021406	6.080029
H	-5.869183	-1.805870	4.535831
O	-4.361232	-1.933120	5.877569
C	-4.023451	-1.590278	7.210293
H	-4.873629	-1.748541	7.878403
H	-3.209459	-2.242468	7.520581
H	-3.692871	-0.551848	7.306619
P	-2.593483	-3.623374	2.863740
Cl	-4.693903	-3.993000	2.526628
C	-2.099770	-5.269911	3.621550
Mo	-1.654055	-3.044051	0.549002
C	-2.879060	-4.466467	-0.247970
O	-3.558548	-5.271933	-0.686634
C	-0.455722	-1.723696	1.516925
O	0.227028	-0.990621	2.070365
C	-0.124739	-4.376511	0.510079
O	0.755970	-5.102843	0.420293
C	-1.056984	-2.400553	-1.226251
O	-0.720294	-2.022878	-2.260876
C	-3.180833	-1.693677	0.503509
O	-4.025623	-0.926691	0.446566
C	-0.635183	-5.080767	4.025327
H	-0.515187	-4.258832	4.735966
H	-0.264142	-5.987457	4.509868
H	0.011791	-4.878835	3.171941
C	-2.925515	-5.524626	4.882895
H	-2.573089	-6.430434	5.384634
H	-2.841940	-4.698130	5.591633
H	-3.982016	-5.662953	4.654879
C	-2.244543	-6.447998	2.667010
H	-1.938210	-7.376447	3.157883
H	-3.278969	-6.571200	2.344077
H	-1.629059	-6.325848	1.776520

TS(**5^{tBu}+2e→13^{tBu}**):

E = -2446.01353410206 au
ZPE = 0.52828332 au
ν = -600.04 cm^{-1}

Li	-2.829312	-0.147340	4.854951
O	-4.148563	1.241184	5.488072
C	-3.374781	2.385376	5.810567
H	-3.152127	2.879340	4.863583
H	-3.935910	3.090822	6.430781
C	-2.088687	1.993832	6.508571
H	-2.272635	1.643535	7.532828
H	-1.437006	2.872373	6.578629
O	-1.482030	0.969152	5.744760
C	-0.230909	0.545435	6.252470
H	-0.325862	0.154694	7.270054
H	0.141415	-0.244079	5.603541
H	0.488296	1.368640	6.258526
C	-4.913817	0.688323	6.549701
H	-4.436715	0.869171	7.518900
H	-5.908513	1.143285	6.585777
C	-5.034981	-0.796162	6.281947
H	-5.595015	-1.280762	7.088004
H	-5.578045	-0.968627	5.350456
O	-3.752462	-1.383748	6.120246
C	-3.113352	-1.737557	7.334339
H	-3.707058	-2.470731	7.885789
H	-2.152700	-2.182347	7.082468
H	-2.935372	-0.874670	7.982833
O	-2.747160	-0.495662	3.050122
C	-1.847921	-1.367504	2.412698
H	-1.449925	-0.975490	1.473573
C	-0.757499	-1.928194	3.270282
C	0.558888	-1.557404	3.014480
C	1.591983	-1.983784	3.838617
C	1.316352	-2.786978	4.935084
C	0.006795	-3.170697	5.193112
C	-1.022015	-2.746997	4.366254
H	0.779306	-0.925286	2.162200
H	2.610120	-1.685831	3.624083
H	2.118100	-3.121760	5.580357
H	-0.212669	-3.815475	6.034648
H	-2.029995	-3.084780	4.571254
P	-2.827263	-4.333185	1.132579
C	-3.143845	-2.005679	2.309655
H	-3.802725	-1.730976	1.501945
H	-3.539905	-2.607227	3.112756
Mo	-0.728650	-5.221400	-0.066399
C	-1.359375	-7.112490	0.357185
O	-1.705250	-8.173591	0.599235
C	-0.241652	-3.286747	-0.430347
O	0.030977	-2.196158	-0.647525
C	0.179670	-5.164224	1.765195

O	0.741877	-5.197932	2.756728
C	0.976295	-5.859779	-0.836692
O	1.970148	-6.239403	-1.279424
C	-1.529725	-5.368793	-1.920278
O	-1.910720	-5.483420	-2.994910
C	-4.490359	-4.713396	0.329643
Cl	-3.003645	-5.557285	2.892776
C	-5.628738	-4.382614	1.296984
H	-6.591115	-4.504382	0.791707
H	-5.628171	-5.040895	2.165182
H	-5.577663	-3.353141	1.655391
C	-4.634881	-6.155237	-0.140163
H	-3.889083	-6.414419	-0.890582
H	-4.534535	-6.854841	0.690410
H	-5.621663	-6.311700	-0.586997
C	-4.568881	-3.754303	-0.861974
H	-4.526937	-2.708997	-0.547836
H	-3.767016	-3.914716	-1.582131
H	-5.514787	-3.896999	-1.390745

13^{tBu}:

E = -2446.08894221172 au

ZPE = 0.53089434 au

Li	-2.750373	-0.157542	4.676466
O	-4.012735	1.381686	5.242502
C	-3.166099	2.467097	5.576194
H	-2.844327	2.903829	4.629424
H	-3.701010	3.241647	6.135099
C	-1.960658	2.001340	6.367616
H	-2.236267	1.706627	7.389428
H	-1.247233	2.829930	6.453603
O	-1.396647	0.908223	5.677217
C	-0.230341	0.386331	6.278422
H	-0.425149	0.044201	7.300002
H	0.099767	-0.459858	5.679693
H	0.567418	1.134171	6.308441
C	-4.863371	0.919258	6.279302
H	-4.430621	1.113680	7.266702
H	-5.829938	1.432564	6.243480
C	-5.065050	-0.566132	6.068616
H	-5.709947	-0.970667	6.856425
H	-5.559432	-0.744583	5.111334
O	-3.824985	-1.247870	6.015148
C	-3.268145	-1.556112	7.277192
H	-3.936409	-2.203141	7.852415
H	-2.333396	-2.085141	7.101032
H	-3.052364	-0.665208	7.875094
O	-2.538082	-0.622009	3.004108
C	-2.001118	-1.745090	2.461400
H	-1.531489	-1.548459	1.477250
C	-0.894033	-2.287713	3.352958
C	0.422453	-1.893900	3.122174
C	1.445879	-2.250899	3.988995
C	1.167177	-3.013523	5.114726
C	-0.141049	-3.408022	5.362516
C	-1.160028	-3.043226	4.493139
H	0.649709	-1.294568	2.247413
H	2.461421	-1.935346	3.784277
H	1.961633	-3.299497	5.792274
H	-0.369151	-4.006063	6.236521
H	-2.172408	-3.359844	4.710863
P	-2.796388	-4.328542	1.278445
C	-3.132429	-2.757091	2.181002
H	-3.854327	-2.222473	1.558063
H	-3.658896	-3.004282	3.105736
Mo	-0.712075	-4.792560	-0.103228
C	-1.124810	-6.780121	0.074583
O	-1.304374	-7.904375	0.144242
C	-0.211880	-2.837017	-0.408164
O	0.131857	-1.782763	-0.678593
C	0.325460	-4.934905	1.667105
O	0.954125	-5.129703	2.594262
C	0.969444	-5.273217	-1.041541
O	1.943384	-5.557794	-1.576177
C	-1.617406	-4.782009	-1.917431
O	-2.048058	-4.807029	-2.976200
C	-4.487992	-4.731670	0.561051
Cl	-2.727420	-5.700754	2.863293
C	-5.591267	-4.639818	1.615264
H	-6.549704	-4.864484	1.141947
H	-5.449617	-5.356947	2.422884
H	-5.672591	-3.644251	2.050515
C	-4.470687	-6.137775	-0.033152
H	-3.748236	-6.235966	-0.842488
H	-4.252775	-6.898231	0.716661
H	-5.455155	-6.361279	-0.449694
C	-4.759944	-3.709848	-0.548300
H	-4.879531	-2.696033	-0.166260
H	-3.979205	-3.690263	-1.307255
H	-5.692502	-3.975601	-1.050107

TS(**5^{tBu}+2e→13^{tBu*}**):

E = -2446.01341330735 au

ZPE = 0.52833851 au

ν = -543.35 cm^{-1}

Li	-3.475746	-0.108040	4.787849
O	-5.393845	0.449207	4.458414
C	-5.437248	1.842222	4.718188
H	-5.033703	2.330499	3.829832
H	-6.463097	2.197027	4.856138
C	-4.602797	2.195119	5.932048

H	-5.076612	1.854341	6.862001
H	-4.505357	3.284805	5.997962
O	-3.338041	1.579020	5.777887
C	-2.446186	1.842496	6.844307
H	-2.858345	1.506597	7.800616
H	-1.523473	1.301527	6.647305
H	-2.222526	2.910185	6.914750
C	-6.240724	-0.362285	5.259747
H	-6.406164	0.087401	6.244793
H	-7.220558	-0.481248	4.786973
C	-5.565580	-1.710150	5.394114
H	-6.167392	-2.372439	6.024510
H	-5.469085	-2.183209	4.414520
O	-4.250566	-1.563394	5.908410
C	-4.179385	-1.495583	7.321017
H	-4.562276	-2.413837	7.773329
H	-3.131631	-1.382078	7.591940
H	-4.737250	-0.647832	7.730016
O	-2.473538	-0.602499	3.330731
C	-2.074287	-1.923689	3.108038
H	-2.175022	-2.274890	2.080049
H	-2.452552	-2.672316	3.808975
C	-0.347902	-0.997767	4.791628
C	0.509031	0.081057	5.007748
C	1.014257	0.344831	6.272126
C	0.656225	-0.459489	7.345455
C	-0.217876	-1.521328	7.144297
C	-0.717288	-1.787953	5.879392
H	0.786807	0.716871	4.175829
H	1.685585	1.180836	6.418447
H	1.051858	-0.259670	8.332555

H	-0.501904	-2.153960	7.975506
H	-1.379395	-2.630761	5.738981
P	0.868093	-3.256657	2.847831
C	-0.829316	-1.258292	3.426893
H	-0.331937	-0.745044	2.615280
C	2.685182	-2.847216	3.130831
Mo	0.210072	-5.757028	2.992942
C	1.342682	-6.055297	1.318107
O	1.960549	-6.242788	0.377539
C	1.754943	-6.341866	4.174537
O	2.590988	-6.744489	4.844772
C	-0.402743	-7.635189	2.967294
O	-0.757639	-8.730641	2.938620
C	-0.896413	-5.415497	4.653297
O	-1.539238	-5.274780	5.591794
C	-1.371600	-5.248353	1.816420
O	-2.275506	-5.009240	1.159041
Cl	0.730215	-2.831962	0.741108
C	2.862826	-2.933335	4.650408
H	3.899532	-2.706534	4.912862
H	2.224910	-2.219834	5.174870
H	2.639799	-3.926564	5.040308
C	3.650550	-3.793066	2.427580
H	3.536149	-4.821619	2.766610
H	3.507142	-3.775684	1.346618
H	4.683918	-3.493176	2.627970
C	2.974592	-1.415159	2.680046
H	3.996902	-1.139961	2.954887
H	2.882797	-1.303873	1.600087
H	2.303486	-0.698083	3.152234

13^{tBu*}: E = -2446.08458108058 au

ZPE = 0.53111145 au

Li	-3.325948	-0.148546	4.613675
O	-5.384452	0.160351	4.426227
C	-5.613280	1.492804	4.845558
H	-5.349155	2.129759	3.999675
H	-6.668073	1.670865	5.078426
C	-4.757105	1.844620	6.044831
H	-5.097036	1.322062	6.949435
H	-4.838869	2.920090	6.243261
O	-3.427878	1.479865	5.739412
C	-2.503893	1.748009	6.773151
H	-2.779485	1.236222	7.700845
H	-1.531425	1.384185	6.448199
H	-2.434695	2.820831	6.974711
C	-6.055512	-0.848322	5.164211
H	-6.271684	-0.516659	6.185779
H	-7.013208	-1.096603	4.694983
C	-5.159125	-2.068299	5.180610
H	-5.636544	-2.876740	5.744949
H	-4.994944	-2.428235	4.162494
O	-3.885777	-1.750107	5.709317
C	-3.812456	-1.779076	7.118668
H	-4.042393	-2.776912	7.502300

H	-2.792599	-1.523313	7.398071
H	-4.490115	-1.062139	7.593310
O	-2.274517	-0.356172	3.232272
C	-1.630232	-1.512456	2.972671
H	-1.697476	-1.824663	1.910530
H	-2.008503	-2.385255	3.546721
C	0.148801	-0.976245	4.704578
C	0.814890	0.223134	4.949994
C	1.112011	0.629443	6.243528
C	0.735775	-0.156579	7.324136
C	0.038661	-1.335133	7.095508
C	-0.258223	-1.733630	5.800211
H	1.115055	0.845456	4.115207
H	1.637762	1.561752	6.405632
H	0.973543	0.151342	8.334042
H	-0.270346	-1.952889	7.929486
H	-0.803300	-2.652217	5.646361
P	0.757880	-3.010859	2.856954
C	-0.108314	-1.414335	3.290672
H	0.324569	-0.668086	2.617666
C	2.593352	-2.782061	3.215844
Mo	-0.336446	-5.312392	3.171112

C	0.853304	-5.969078	1.646249
O	1.465632	-6.404902	0.789391
C	0.995286	-5.975847	4.558702
O	1.697157	-6.421698	5.341539
C	-1.144505	-7.122014	3.186754
O	-1.605903	-8.171936	3.183267
C	-1.644251	-4.864298	4.667905
O	-2.419868	-4.740859	5.496935
C	-1.745068	-4.748680	1.798175
O	-2.554057	-4.504792	1.034261
Cl	0.776730	-2.748039	0.770981
C	2.817952	-2.844108	4.731504
H	3.890708	-2.933137	4.916529
H	2.469425	-1.950459	5.242744
H	2.337989	-3.703885	5.197271
C	3.358239	-3.933303	2.560591
H	3.077255	-4.902768	2.970826
H	3.226754	-3.962351	1.479507
H	4.424274	-3.798538	2.755759
C	3.121807	-1.455095	2.674673
H	4.184339	-1.376680	2.915929
H	3.026834	-1.381533	1.592248
H	2.626557	-0.595832	3.123213

VIa: E = -495.081498512037 au

ZPE = 0.0713693 au

P	-0.772337	0.185134	0.555155
H	-1.077327	1.357766	-0.209769
O	0.597575	-0.366922	-0.254153
C	-1.522166	-1.013577	-0.663643
H	-2.348317	-0.673795	-1.283652
H	-1.786680	-1.963178	-0.199824
C	-0.127545	-1.008404	-1.307127
H	0.295237	-1.990944	-1.530774
H	-0.105124	-0.405457	-2.221943

VIb: E = -570.270231850458 au

ZPE = 0.07684182 au

P	-0.746726	0.151892	0.498048
H	-1.116963	1.460598	0.110782
O	0.575361	-0.194193	-0.405056
C	-1.517662	-1.010466	-0.678904
H	-2.327532	-0.617885	-1.290779
H	-1.828403	-1.947460	-0.220658
C	-0.128173	-1.044515	-1.336879
H	0.331611	-2.032493	-1.366655
H	-0.110450	-0.613513	-2.338406
O	-0.718200	-0.045810	1.955510

VIc: E = -892.858332409022 au

ZPE = 0.07484519 au

P	-0.746203	0.154697	0.497266
H	-1.112873	1.447904	0.068374
O	0.585372	-0.220889	-0.398806
C	-1.514821	-1.014157	-0.685973
H	-2.332340	-0.623663	-1.288379
H	-1.816660	-1.947606	-0.215626
C	-0.127607	-1.043879	-1.346927
H	0.325014	-2.034045	-1.401400
H	-0.111159	-0.590511	-2.339145
S	-0.734628	-0.059124	2.390182

VId: E = -2895.24277440812 au

ZPE = 0.0741301 au

P	-0.744849	0.160423	0.480915
H	-1.115255	1.450045	0.041902
O	0.587377	-0.222078	-0.414443
C	-1.514028	-1.015553	-0.698099
H	-2.332646	-0.625742	-1.299406
H	-1.815495	-1.947006	-0.223827
C	-0.127242	-1.046567	-1.360228
H	0.324778	-2.037005	-1.413036
H	-0.111968	-0.594534	-2.353151
Se	-0.736577	-0.053256	2.518941

VIe: E = -762.558310321743 au

ZPE = 0.07359119 au

P	-0.742786	0.171326	0.450788
H	-1.123880	1.455296	0.007995

O	0.589362	-0.218912	-0.444551
C	-1.513292	-1.015527	-0.719871
H	-2.332186	-0.629122	-1.322744
H	-1.815035	-1.941805	-0.236174
C	-0.126262	-1.052472	-1.381929
H	0.325441	-2.043380	-1.425414
H	-0.111749	-0.608641	-2.378609
Te	-0.735520	-0.048036	2.73007

References

1. Kyri, A.W.; Gleim, F.; García Alcaraz, A.; Schnakenburg, G.; Espinosa Ferao, A.; Streubel, R. "Low-coordinate" 1,2-oxaphosphetanes – a new opportunity in coordination and main group chemistry. *Chem. Commun.* **2018**, *54*, 7123–7126, doi:10.1039/C8CC02963F.
